# Lack of the PGA exopolysaccharide in *Salmonella* as an adaptive trait for survival in the host

**DOI:** 10.1371/journal.pgen.1006816

**Published:** 2017-05-24

**Authors:** Maite Echeverz, Begoña García, Amaia Sabalza, Jaione Valle, Toni Gabaldón, Cristina Solano, Iñigo Lasa

**Affiliations:** 1 Navarrabiomed-Universidad Pública de Navarra-Departamento de Salud, IDISNA, Pamplona, Spain; 2 Centre for Genomic Regulation (CRG), Barcelona Institute of Science and Technology, Barcelona, Spain; Universidad de Sevilla, SPAIN

## Abstract

Many bacteria build biofilm matrices using a conserved exopolysaccharide named PGA or PNAG (poly-β-1,6-*N*-acetyl-D-glucosamine). Interestingly, while *E*. *coli* and other members of the family *Enterobacteriaceae* encode the *pgaABCD* operon responsible for PGA synthesis, *Salmonella* lacks it. The evolutionary force driving this difference remains to be determined. Here, we report that *Salmonella* lost the *pgaABCD* operon after the divergence of *Salmonella* and *Citrobacter* clades, and previous to the diversification of the currently sequenced *Salmonella* strains. Reconstitution of the PGA machinery endows *Salmonella* with the capacity to produce PGA in a cyclic dimeric GMP (c-di-GMP) dependent manner. Outside the host, the PGA polysaccharide does not seem to provide any significant benefit to *Salmonella*: resistance against chlorine treatment, ultraviolet light irradiation, heavy metal stress and phage infection remained the same as in a strain producing cellulose, the main biofilm exopolysaccharide naturally produced by *Salmonella*. In contrast, PGA production proved to be deleterious to *Salmonella* survival inside the host, since it increased susceptibility to bile salts and oxidative stress, and hindered the capacity of *S*. Enteritidis to survive inside macrophages and to colonize extraintestinal organs, including the gallbladder. Altogether, our observations indicate that PGA is an antivirulence factor whose loss may have been a necessary event during *Salmonella* speciation to permit survival inside the host.

## Author summary

During bacterial evolution, specific traits that optimize the organism’s fitness are selected. The production of exopolysaccharides is widespread among bacteria in which they play a protective shielding role as main constituents of biofilms. In contrast to closely related siblings, *Salmonella* has lost the capacity to produce the exopolysaccharide PGA. Our study reveals that *Salmonella* lost *pga* genes, and that the driving force for such a loss may have been the detrimental impact that PGA has during *Salmonella* invasion of internal organs where it augments the susceptibility to bile salts and oxygen radicals, reducing bacterial survival inside macrophages and rendering *Salmonella* avirulent. These results suggest that gene-loss has played an important role during *Salmonella* evolution.

## Introduction

*Escherichia coli* and *Salmonella enterica* are the two core species of the family *Enterobacteriaceae*, that constitutes a diverse group of bacteria that generally inhabit the gastrointestinal tract of animals. Although these two species are closely related, *E*. *coli* comprises commensal bacteria that do not normally cause disease, with the exception of certain pathogenic strains, whereas all members of *S*. *enterica* are considered pathogenic. Hence, an intriguing issue regarding bacterial evolution is the identification of determinants that make *Salmonella* able to establish parasitic interactions but enable *E*. *coli* to establish beneficial interactions with the human host. In this regard, it is believed that a combination of different genetic factors accounts for such a difference in virulence: first, *Salmonella* harbor virulence genes that are not present in *E*. *coli*; second, *Salmonella* may have lost genes from the ancestral core genome that if present, would diminish its pathogenic potential; third, *E*. *coli* may carry a virulence suppressor gene(s) that interferes with the synthesis and/or stability of a virulence protein(s); and fourth, *Salmonella* and *E*. *coli* may differ in the regulation of cellular factors important for survival in the host [1–3].

An intriguing difference between *Salmonella* and *E*. *coli* that might account for their distinctive lifestyles as regards the human host is the exopolysaccharide that each species uses to build the biofilm matrix. Bacteria spend most of their lives inside a biofilm surrounded by a highly hydrated layer that provides protection against desiccation, diffusion of antibiotics, toxic metal ions and other compounds, predation by protozoans and the host immune system, amongst others [4,5]. Diversity in biofilm exopolysaccharides composition is high, with some bacterial species being able to produce different types depending on the environmental conditions [6,7]. In parallel to such high diversity and for reasons that remain unknown, a wide range of phylogenetically distant bacteria make use of the same exopolysaccharide to embed themselves inside a biofilm. One example of a “universal” exopolysaccharide is cellulose, composed of β(1–4)-linked D-glucose units, used by a wide variety of bacteria, including both *E*. *coli* and *Salmonella* [8–10], as a significant biofilm matrix component. Another example corresponds to a homopolysaccharide composed of N-acetylglucosamine with β(1–6) glycosidic linkage [11]. Production of this exopolysaccharide was firstly described in *Staphylococcus epidermidis and S*. *aureus* where it was referred to as PIA/PNAG [12–14]. Later on, the synthesis of a similar exopolysaccharide was also reported in *E*. *coli* where it was named as PGA [15], and also in *Acinetobacter baumannii* [16], *Klebsiella pneumoniae* [17], *Bordetella bronchiseptica* and *B*. *pertussis* [18,19], *Actinobacillus pleuropneumoniae* [20], *Yersinia pestis* [21], *Burkholderia* species [22] and *Bacillus subtilis* [23]. In these bacteria, several functions have been ascribed to PGA such as surface attachment, intercellular adhesion, biofilm formation, epithelial cell attachment, and resistance to antibiotics, antimicrobial peptides and human PMNs [16,19,22,24–29]. In *E*. *coli*, the production, modification, and export of PGA requires the machinery encoded by the *pgaABCD* operon [15]. PgaA and PgaB are needed for poly-GlcNAc export and PgaC and PgaD are necessary for poly-GlcNAc synthesis [30–33]. As it generally occurs for bacterial exopolysaccharides, PGA synthesis is allosterically activated by the second messenger bis-(3’-5’)-cyclic dimeric GMP (c-di-GMP) [30,34,35]. Strikingly, *Salmonella* lacks the *pgaABCD* operon and any identifiable genetic loci similar to *pga* required for PGA synthesis.

Here, we pursue the reasons that explain why *E*. *coli* and *Salmonella* differ in their capacity to produce the PGA exopolysaccharide. We provide evidence that production of PGA reduces *Salmonella* resistance against bile salts and its capacity to survive inside macrophages, completely impairing the infection cycle and rendering *Salmonella* avirulent. Together, these observations highlight the relevance of gene loss in the adaptation to novel pathogenic niches and define the loss of the PGA exopolysaccharide as a landmark event during *Salmonella* speciation.

## Results

### Absence of the *pgaABCD* operon in *Salmonella* is likely the result of a secondary loss

**Fig 1:**
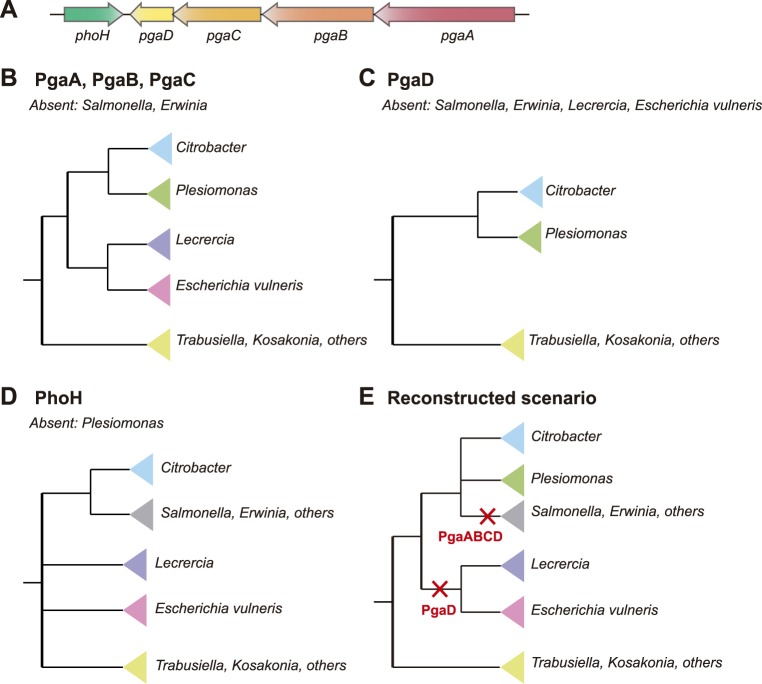
Schematic representation of the phylogenetic relationships of homologues of the proteins analyzed in groups that are closely related to *Salmonella*. **(A)** Schematic representation of the *pgaABCD* operon and the *phoH* gene in *E*. *coli* K-12 MG1655. The proteins PgaA, PgaB, and PgaC showed the same pattern **(B)**, with PgaD showing a more restricted distribution **(C)**. PhoH showed a phylogenetic pattern **(D)** that is compatible with those of PgaABCD if three losses are inferred **(E)**. PgaABCD in *Salmonella/Erwinia*, top red cross; PgaD in *Leclercia/E*. *Vulneris* (bottom red cross), and PhoH in *Plesiomonas* (not shown in the tree). The full phylogenies comprising the closest 250 homologues are provided in S1 dataset.

To investigate whether the presence of the *pgaABCD* operon in *Escherichia* and its absence in *Salmonella* is due to a lineage-specific acquisition in *Escherichia* or to a loss in *Salmonella*, we performed different comparative and phylogenetic analyses (see Materials and Methods). Analysis of the genomic context of *E*. *coli* PgaA protein in the STRING database [36] correctly identified the presence of four genes in the *pgaABCD* operon as significantly associated using exclusively gene neighborhood and gene co-occurrence information. The gene cluster, often only presenting the three upstream genes, is widespread among *Enterobacteriaceae*, being present in 22 species of the 83 available in the database. Besides *Escherichia*, the genera with the cluster include *Klebsiella*, *Pectobacterium*, *Yersinia*, *Citrobacter*, and *Enterobacter*, among others. Analyses of the presence/absence of the genes revealed a similar pattern, confirming the absence of the genes in *Salmonella* species and other genera. Importantly, both analyses revealed a patchy presence/absence pattern, including many recent apparent losses within some genera such as *Citrobacter* or *Escherichia*. We then reconstructed individual phylogenies in each of the genes in the cluster by aligning the top 500 hits of a blastP search in NCBI nr database, after setting a filter to exclude sequences assigned to *E*. *coli*. All the top hits belonged to related species of *Enterobacteriaceae* excluding the possibility of recent, independent transfers of the cluster from a non-*Enterobacteriaceae* species. Maximum likelihood phylogenies of the four genes produced roughly similar topological arrangements of the included taxa (schematically depicted in Fig 1). We performed a similar analysis with *phoH*, the gene located in the vicinity of the cluster, encoding a protein with a nucleoside triphosphate hydrolase domain. This gene has a broader distribution, present in 68 of the 83 taxa, a pattern suggesting a vertical inheritance with few independent losses. Importantly, however, for the shared species, the phylogenies of *phoH* and that of the four genes in the *pgaABCD* cluster showed an overall similarity (Fig 1, S1 Fig and S1 Dataset). This indicates that the five genes followed a similar evolutionary history, with the exception of differential loss of genes in alternative lineages. This topology was largely congruent with the species tree for *Enterobacteriaceae* provided in the PATRIC database, which is based on the analysis of several shared genes [37,38], with the notable exception of the position of *Yersinia* or *Serratia* strains. Previous studies have shown that losses are more frequent than lateral transfer in the evolution of prokaryotic genomes [39], and lateral transfer would generate discordance between gene trees [40]. Hence, our results point to an overall dominance of vertical inheritance and differential gene loss in the evolution of this gene cluster within *Enterobacteriaceae*. Considering this scenario, the *pgaABCD* cluster was lost somewhere after the divergence of *Salmonella* and *Citrobacter* clades, and previous to the diversification of the currently sequenced *Salmonella* strains.

### Expression of the *pgaABCD* operon in *Salmonella* is sufficient for PGA production

**Fig 2:**
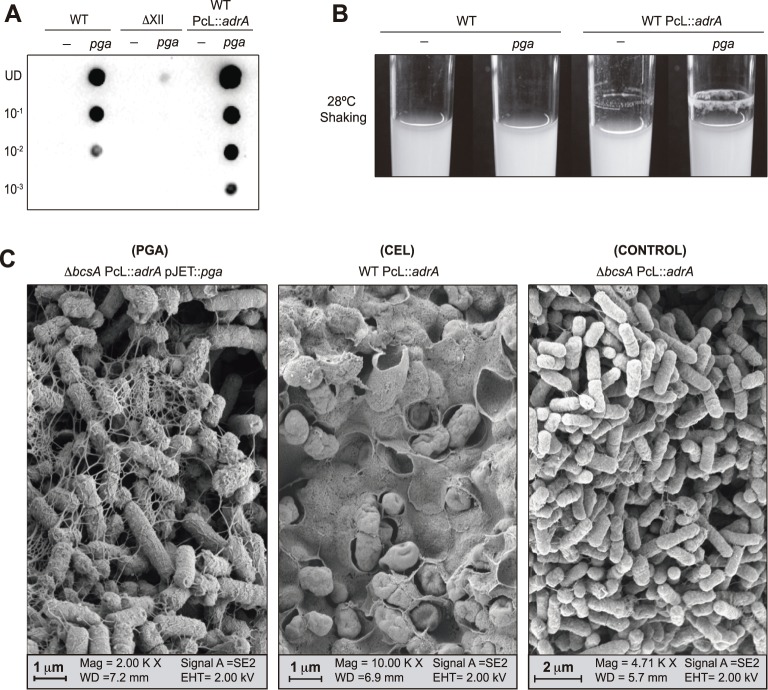
Heterologous expression of the *pgaABCD* operon in *Salmonella* drives PGA synthesis in response to c-di-GMP levels and makes *Salmonella* able to build a PGA mediated biofilm. **(A)** Dot blot analysis of the PGA accumulated by *S*. Enteritidis wild type, ΔXII and WT PcL::*adrA* and their corresponding transformed strains with plasmid pJET::*pga* after 48 hours of incubation in LB or LB Cb broth, at room temperature, under static conditions. Serial dilutions (1/10) of the samples were spotted onto nitrocellulose membranes and PGA production was detected with specific anti PIA/PNAG antibodies. UD; undiluted sample. **(B)** Biofilm phenotypes of wild type, WT PcL::*adrA* and their corresponding transformed strains with plasmid pJET::*pga* after incubation in LB or LB Cb broth, at 28°C for 16 hours under shaking conditions. A ring of cells adhered to the glass wall at the air–liquid interface corresponds with a PGA based biofilm. **(C)** Scanning electron microscopy analysis of the biofilms developed by the PGA overproducing strain, *ΔbcsA* PcL::*adrA* pJET::*pga*, after incubation in LB broth, at 28°C for 16 hours under shaking conditions and by the cellulose overproducing strain, WT PcL::*adrA*, after incubation in LB broth, at room temperature under static conditions for 72 hours. A control strain, *ΔbcsA* PcL::*adrA*, that produces neither PGA nor cellulose was also analyzed after incubation in LB broth, at 28°C for 16 hours under shaking conditions.

If *Salmonella* inability to synthesize PGA is exclusively due to loss of the *pgaABCD* operon, complementation with *pgaABCD* should be sufficient to restore PGA production. To test this hypothesis, we transformed a *S*. Enteritidis wild type strain with plasmid pJET::*pga* carrying the *pgaABCD* operon of *E*. *coli* MG1655 under the control of its own promoter, and analyzed PGA synthesis upon growth under *Salmonella* biofilm forming conditions (incubation in LB broth, at room temperature, without shaking) using a dot blot assay and an anti-PIA/PNAG antiserum. As expected, PGA was not detected in cell extracts of the wild type strain whereas WT pJET::*pga* produced PGA and accumulated it throughout the incubation time (Fig 2A and S2A Fig).

We next examined if, as it happens in *E*. *coli* [30], PGA production in *Salmonella* is also dependent on c-di-GMP. To do so, we firstly complemented *S*. Enteritidis ΔXII with the *pgaABCD* operon. *S*. Enteritidis ΔXII is a multiple mutant, derivative of the wild type strain, carrying mutations in all twelve genes encoding GGDEF domain proteins (putative c-di-GMP synthases) and thus incapable of synthesizing c-di-GMP [41,42]. The dot-blot assay showed that ΔXII pJET::*pga* was unable to produce PGA, confirming that c-di-GMP is indeed essential for PGA production in *Salmonella* (Fig 2A). Secondly, we constructed a strain in which the *adrA* gene of *Salmonella*, which encodes a c-di-GMP synthase, is under the control of a constitutive promoter. This strain (WT PcL::*adrA*) constitutively produces high levels of c-di-GMP. Upon transformation with pJET::*pga*, this strain produced higher PGA levels than the wild type strain (Fig 2A and S2A Fig), showing that heterologous PGA synthesis in *Salmonella* is commensurate to cellular c-di-GMP levels. Finally, and in order to identify the source of c-di-GMP in WT pJET::*pga* that triggers PGA production, we used a collection of twelve strains, derivatives of ΔXII, each of which contained the chromosomal copy of a single gene encoding a GGDEF domain protein in the original wild type genomic location [41,42]. The analysis of cell extracts of each strain complemented with pJET::*pga* showed that five GGDEF domain proteins, namely AdrA, YedQ, YegE, YfiN and SEN4316, when individually present in the chromosome of the cell, were able to elicit c-di-GMP dependent PGA synthesis (S2B Fig). Overall, these results showed that heterologous *pgaABCD* expression is sufficient to restore *Salmonella* capacity to synthesize PGA and that this synthesis is dependent on c-di-GMP levels that are provided as a pool by different *Salmonella* c-di-GMP synthases.

### Heterologous PGA production bestows biofilm lifestyle behavior upon *Salmonella*

In staphylococcal cells, production of PGA can be visualized as a ring of cells adhered to the glass wall at the air–liquid interface, when bacteria are incubated in a glass tube under shaking conditions [43]. To investigate whether *Salmonella* is likewise able to build a PGA mediated biofilm, we analyzed biofilm formation by WT pJET::*pga* and WT PcL::*adrA* pJET::*pga* after incubation in LB broth, at 28°C for 16 hours under shaking conditions. Only the second strain, which produces constitutive and high levels of c-di-GMP, produced a visible ring of bacteria adhered to the glass wall (Fig 2B). Structure of this PGA based biofilm was then compared with the natural cellulose based biofilm formed by *Salmonella* using scanning electron microscopy (Fig 2C). To do so, we used a cellulose overexpressing strain (WT PcL::*adrA*), a PGA positive and cellulose minus strain (*ΔbcsA* PcL::*adrA* pJET::*pga*) and a control strain that produces neither cellulose nor PGA (*ΔbcsA* PcL::*adrA*). In the case of the PGA dependent biofilm, cells were tangled up in an abundant extracellular matrix mesh that interconnected the bacteria. Furthermore, spherical, knob-like structures were evident on the bacterial cell surface. These knob-like structures have already been described in PGA (PIA/PNAG) related biofilms of *E*. *coli*, *Yersinia pestis* and *Staphylococcus epidermidis* [44–46]. On the other hand, bacteria inside a cellulose based biofilm were covered by a sheet-like material [47] that totally encased bacteria and that appeared more compact and structured than the PGA biofilm. To further investigate the differences between both types of biofilms, macrocolony biofilms were grown on LB agar plates (S3A Fig) and a water-droplet analysis of colony hydrophobicity was performed [48]. Results showed that a cellulose mediated biofilm is highly hydrophobic, whereas a PGA based biofilm exhibits intermediate hydrophobicity compared with the non-biofilm producing strain, *ΔbcsA* PcL::*adrA* (S3B Fig). Collectively, these findings showed that heterologous PGA expression alongside high c-di-GMP levels enable *Salmonella* to build a PGA mediated biofilm that greatly differs at the structural level from the natural cellulose based biofilm.

### Effects of PGA on *Salmonella* protection from environmental stresses

**Fig 3:**
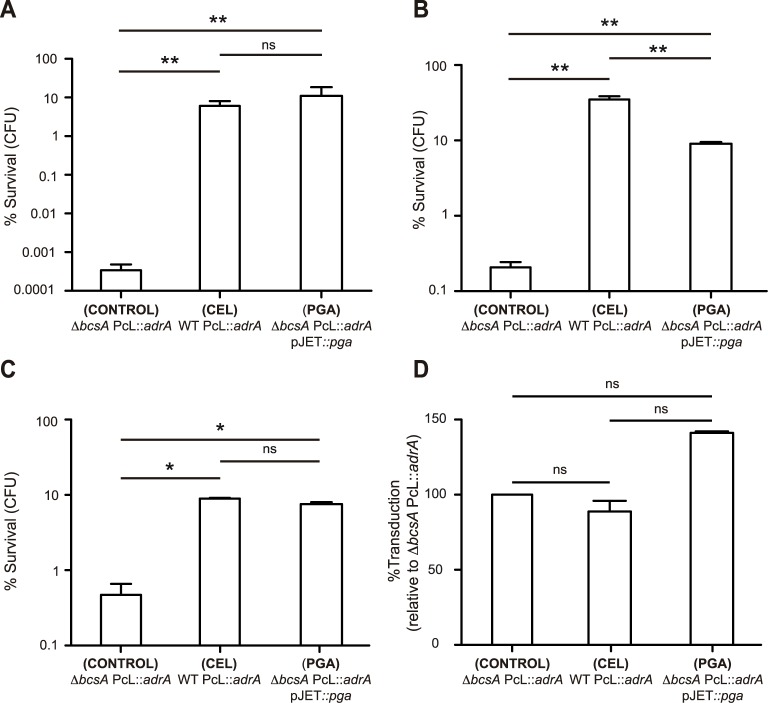
PGA provides, at the most, similar protection to that conferred by cellulose against chlorine treatment, UV light irradiation, heavy metal stress and phage infection. **(A)** The cellulose overproducing strain, WT PcL::*adrA*, the PGA overproducing strain, *ΔbcsA* PcL::*adrA* pJET::*pga*, and the control strain, *ΔbcsA* PcL::*adrA*, were incubated on LB agar or LB agar Cb media for 48h at 28°C. Macrocolonies were then exposed to 200 p.p.m. sodium hypochlorite for 40 min. Surviving bacteria were enumerated by viable plate counts, and their numbers were compared with that of control bacteria that had not been incubated with NaOCl. Note the logarithmic scale in the y axis. The data represent the mean of two independent experiments performed in triplicate. **(B)** Serial dilutions of the cellulose overproducing strain, the PGA overproducing strain and the exopolysaccharide minus strain were plated on N minimal medium containing carbenicillin and after 24 hours of incubation at 28°C, plates were exposed to five minutes of UV light irradiation. Replica plates were not subjected to treatment and served as controls. After 48 hours of incubation at 28°C, numbers of surviving bacteria were counted. Results are shown as % survival relative to untreated samples. *ΔbcsA* PcL::*adrA* and WT PcL::*adrA* carried an empty plasmid so that the three strains could be incubated on the same plates. Note the logarithmic scale in the y axis. The data represent the mean of three independent experiments. **(C)** Macrocolony biofilms were grown on sterile polymer membrane filters and then exposed to 0.5 mM CdCl_2_ for 3 h. Surviving bacteria were enumerated by viable plate counts, and their numbers were compared with that of control bacteria that had not been incubated with CdCl_2_. Note the logarithmic scale in the y axis. The data represent the mean of three independent experiments performed in duplicate. **(D)** Macrocolony biofilms were grown on sterile polymer membrane filters and then subjected to phage infection with a P22 phage lysate generated from a streptomycin resistant strain. Transductants were enumerated by plate counts on LB Sm media and their numbers were compared with those of the exopolysaccharide minus strain, *ΔbcsA* PcL::*adrA*, which defined 100% transduction. The data represent the mean of three independent experiments. Statistical analysis in all assays was carried out using a Mann-Whitney *U* test. ns = no significant difference; * P < 0.05; ** P < 0.01.

Biofilm exopolysaccharides provide protection from the external environment. Thus, a consequence of PGA loss might be a reduction in *Salmonella* resistance to environmental threats, unless another compound assumed such a function. To test this hypothesis, we compared the resistance provided by PGA and cellulose to several environmental stresses. Since it has already been described that cellulose mediates chlorine survival of *Salmonella* and other bacteria [10,49,50], we first analyzed the susceptibility of macrocolony biofilms formed by the cellulose-positive strain (WT PcL::*adrA*) and the PGA-positive cellulose-negative strain (*ΔbcsA* PcL::*adrA* pJET::*pga*) to chlorine. The non-biofilm producing strain, *ΔbcsA* PcL::*adrA*, was used as a control. A 40 min exposure to sodium hypochlorite (200 p.p.m.) caused a decrease of ~5.5 logs in the number of control bacteria, compared to samples treated with only PBS (Fig 3A). Conversely, the same sodium hypochlorite treatment caused a reduction of ~1 log in the number of bacteria inside a cellulose or a PGA based biofilm (Fig 3A). These results determined that the protection against chlorine conferred by PGA is equivalent to that provided by cellulose.

Next, we tested the resistance that PGA and cellulose confer to five minutes of UV light irradiation. Although both exopolysaccharide overproducing strains survived better than the control strain that produces neither polysaccharide, the cellulose-positive cells showed a significantly higher survival rate than the PGA-positive cellulose-negative strain (Fig 3B). Thus, under our experimental conditions, cellulose provides better protection against ultraviolet radiation than PGA.

Microbial biofilm formation and production of extracellular polymeric substances are generally associated with metal resistance and tolerance [51]. To evaluate the protection conferred by cellulose and PGA on *Salmonella* against heavy metal stress, we treated macrocolony biofilms with 0.5 mM cadmium chloride (CdCl_2_). Results indicated that cellulose and PGA confer equal resistance to metal toxicity (Fig 3C).

Phages are found in abundance in environmental settings and bacteria have developed sophisticated mechanisms, including biofilm formation, to limit phage reproduction. To address the impact of cellulose and PGA biofilm extracellular matrices on phage infection, we infected bacteria that had been grown on membrane filters under biofilm forming conditions with a P22 phage lysate and analyzed the transduction frequency of a streptomycin resistance cassette. Results showed that, under our experimental conditions, neither exopolysaccharide protected *Salmonella* from phage infection (Fig 3D).

Overall, these findings suggested that PGA provides, at the most, similar benefits to those conferred by cellulose against environmental threats, at least under the conditions tested. Since both polysaccharides seem to have redundant roles in environmental survival, our results support the idea that during speciation the PGA pathway was lost without affecting survival outside the host during the *Salmonella* cyclic lifestyle.

### PGA production hinders *Salmonella* intramacrophage survival

**Fig 4:**
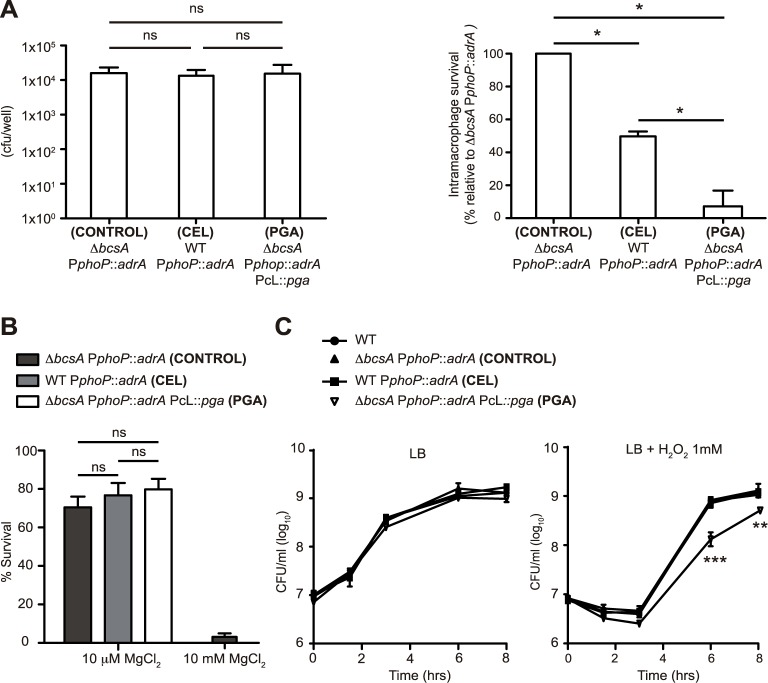
A *Salmonella* strain that makes PGA inside macrophages is highly defective for intramacrophage survival and shows increased sensitivity to H_2_O_2_. **(A)** CFU of phagocytosed bacteria after a 1.5 h incubation with 2 × 10^5^ RAW 264.7 macrophages at a multiplicity of infection of 10 (left panel). Replication within RAW 264.7 macrophages at 18 h postinfection (right panel). Replication of the exopolysaccharide minus strain, *ΔbcsA* P_phoP_::*adrA*, defined 100% intramacrophage survival. Significance was determined by a Mann-Whitney *U* test. The data represent the mean of three independent experiments performed in triplicate. ns = no significant difference; * P < 0.05; ** P < 0.01. **(B)** Strains *ΔbcsA* P_phoP_::*adrA*, WT P_phoP_::*adrA* and *ΔbcsA* P_phoP_::*adrA* PcL::*pga* were grown to logarithmic phase in N minimal medium with 10 μM MgCl_2_. Washed bacteria were diluted 1:10 in LB and LB medium containing Polymyxin B at a final concentration of 2.5 μg ml^-1^, and were incubated for 1 h at 37°C. Samples were diluted in PBS and plated on LB agar plates to assess bacterial viability. Survival values of bacteria incubated in LB Polymyxin are relative to values of bacteria incubated in LB. Strain *ΔbcsA* P_phoP_::*adrA* incubated in N minimal medium with 10 mM MgCl_2_ prior to polymyxin treatment showed a highly sensitive phenotype and served as a control of the resistance phenotype induced by low MgCl_2_ concentrations. Data were analyzed by a Mann-Whitney *U* test. Bars are means of replicates ± s.e. (n = 3). ns = no significant difference. **(C)** Overnight cultures were diluted 1/100 in LB broth or LB broth supplemented with 1 mM H_2_O_2_ and incubated at 37°C with aeration. Aliquots were collected hourly, serially diluted and plated. Statistical analysis was carried out using a two-way analysis of variance combined with the Bonferroni test. ** P < 0.01; *** P < 0.001. Bars are means of replicates ± s.e. (n = 3).

During infection, the ability of *Salmonella* to survive and replicate in the vacuole within host phagocytic cells is essential for systemic disease [52]. To investigate the consequences of PGA production in *Salmonella* intramacrophage replication, we tested the ability of a PGA producing strain to replicate in RAW264.7 murine macrophages and compared it with that of a cellulose producing strain. To guarantee the synthesis of PGA or cellulose inside macrophages, we created *Salmonella* strains displaying high c-di-GMP levels inside these cells through the use of the macrophage activated *phoP* promoter fused to the *adrA* gene [53]. We firstly constructed WT P_phoP_::*adrA* and confirmed that it produced a cellulose based biofilm in response to the low Mg^2+^ signal activating the *phoP* promoter (S4 Fig). Then, we engineered *ΔbcsA* P_phoP_::*adrA* PcL::*pga*, a cellulose mutant that constitutively expresses the PGA synthesis machinery from the chromosome but that synthesizes PGA in a *phoP* dependent fashion (S4 Fig). As a control, we constructed WT *ΔbcsA* P_phoP_::*adrA* producing neither cellulose nor PGA. The three strains were phagocytosed at similar rates and as it has already been described, the cellulose overproducing strain was defective for replication inside macrophages [53], showing an ~50% intramacrophage survival relative to the control strain *ΔbcsA* P_phoP_::*adrA* (Fig 4A). Remarkably, the PGA producing strain was significantly more attenuated than the cellulose overproducing strain, showing a 7% intramacrophage survival relative to the control strain (Fig 4A).

*Salmonella* contained within the phagosomal environment encounter a diversity of antimicrobial factors including cationic antimicrobial peptides (CAMP) and reactive oxygen species (ROS) [54]. To investigate the cause(s) behind the low intramacrophage survival phenotype related to PGA production, we firstly performed one-hour polymyxin susceptibility assays [55] of bacterial cells previously grown under low Mg^2+^ levels, a condition that promotes polymyxin resistance through activation of the PhoP regulon [55,56]. The presence of either polysaccharide, cellulose or PGA, did not have an effect on *Salmonella* polymyxin resistance (Fig 4B). Then, we investigated whether reduced intracellular replication was linked to increased sensitivity to ROS production by assessing the ability to grow in the presence of 1mM H_2_O_2_ (Fig 4C). When wild type *Salmonella* were inoculated into 1 mM peroxide-containing medium at 10^7^ CFU/ml, there was no increase in cell numbers for the first 3 h of incubation, followed by fast recovery [57]. Growth of the cellulose overproducing and control strains were indistinguishable from that of the wild type, whilst the PGA overproducing strain showed a significant viability loss throughout the incubation time (Fig 4C). Taken together, these results indicated that PGA production has a detrimental effect on *Salmonella* intramacrophage survival and that such survival decrease may be partially explained by the fact that PGA makes *Salmonella* more sensitive to oxidative stress.

### PGA production renders *Salmonella* avirulent in mice

**Fig 5:**
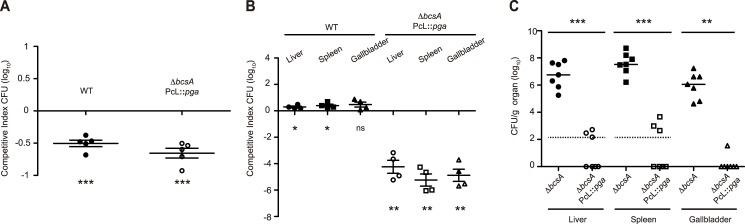
PGA production leads to a complete *Salmonella* attenuation in a mouse model of infection. **(A)** Competitive index analysis of the wild type strain and *ΔbcsA* PcL::*pga* coinoculated with the control strain, *ΔbcsA*, after performing an ileal loop coinfection experiment. Five ileal loops were coinfected with 2x10^7^ cfu containing equal numbers of the control and either the cellulose or PGA producing strains. A CI > 0 indicates the exopolysaccharide producing strain with a colonization advantage compared to the control and a CI < 0 indicates the exopolysaccharide producing strain with a colonization disadvantage over the control. **(B)** CI analysis following intragastric inoculation of four BALBc mice with a 1:1 mixture of the control strain, *ΔbcsA*, and either the wild type or *ΔbcsA* PcL::*pga* strain (total inoculum administered was 2 x 10^8^ cfu). Mice were sacrificed when evident signs of disease (score 2 to 3) were observed, and bacteria were enumerated from livers, spleens and gallbladders. No *ΔbcsA* PcL::*pga* bacteria were recovered from any of the organs analyzed; detection limit was 133 cfu/g in livers and spleens and 2 cfu/organ in the gallbladder. This detection limit was used to calculate the output corresponding to mice coinoculated with *ΔbcsA* and *ΔbcsA* PcL::*pga*. The plots display values obtained from individual samples and the mean CI is represented by horizontal bars. P-values were determined by a Student *t* test. ns = no significant difference; *P < 0.05; ** P < 0.01. **(C)** Virulence of *ΔbcsA* and *ΔbcsA* PcL::*pga* in mice inoculated intragastrically. Seven mice were infected with 1 x 10^8^ cfu of the indicated strain. Mice inoculated with *ΔbcsA* were sacrificed when evident signs of disease (score 2 to 3) were observed, whilst mice inoculated with *ΔbcsA* PcL::*pga* were sacrificed at the end of the experiment. Bacterial loads in livers, spleens and gallbladders were analyzed. The dashed line indicates detection limit (133 cfu/g in livers and spleens). The plots display values obtained from individual samples and the median is represented by horizontal bars. Differences in colonization were statistically analyzed by using the Mann-Whitney U test. ** P < 0.01; *** P < 0.001.

Since heterologous expression of PGA makes *Salmonella* less capable to survive inside macrophages, we hypothesized that PGA production might result in virulence attenuation upon infection by the natural oral route of BALB/c mice, which are susceptible to systemic infection with *Salmonella*. Taking into account that c-di-GMP is involved in modulating the innate immune response [58,59], we constructed a *Salmonella* strain that constitutively produced PGA from the chromosome, without altering natural c-di-GMP levels. As expected, levels of PGA production by this strain, WT PcL::*pga*, were lower than those produced by WT pJET::*pga* (S5 Fig). Additionally, the *bcsA* gene was mutated in this strain, resulting in *ΔbcsA* PcL::*pga*, which produced PGA but not cellulose. Thus, virulence assays were carried out by comparing the pathogenic behavior of the control strain, *ΔbcsA*, which produces neither cellulose nor PGA, with that of either the PGA producing strain *ΔbcsA* PcL::*pga* or the wild type strain, which produces natural levels of cellulose during infection. These two strains did not show any discernable fitness cost compared to *ΔbcsA* when grown in LB broth at 37°C (S6 Fig). Firstly, the impact of PGA and cellulose synthesis on the capacity of *Salmonella* to adhere and invade the intestinal epithelium was analyzed by carrying out a competitive index analysis in an ileal loop coinfection experiment (Fig 5A). Both the wild type and *ΔbcsA* PcL::*pga* strains showed reduced capacity to adhere and invade the intestinal epithelium compared with the control strain, *ΔbcsA*. Secondly, we assessed the level of organ colonization following oral co-inoculation of the control strain, *ΔbcsA*, and either the wild type or *ΔbcsA* PcL::*pga* strain. In the case of mice co-infected with the wild type and *ΔbcsA* strains, the bacterial burden of the wild type was slightly higher than that of *ΔbcsA* in all organs analyzed (livers, spleens and gallbladders). Conversely, the PGA producing strain showed to be extremely attenuated, since no *ΔbcsA* PcL::*pga* bacteria were recovered from the organs examined after co-infection with the control strain (Fig 5B). To exclude the possibility that the control strain outcompetes the PGA producing strain when coinfection experiments are performed, we next compared the virulence of *ΔbcsA* and *ΔbcsA* PcL::*pga* strains by carrying out single infection experiments. Results confirmed that the PGA producing strain was highly attenuated, since mice inoculated with *ΔbcsA* PcL::*pga* did not show any disease symptom and most of them presented bacterial counts under the detection limit in livers, spleens and gallbladders (Fig 5C). It is important to note that in the case of gallbladders, the entire organ was plated and that six out of seven gallbladders from mice inoculated with *ΔbcsA* PcL::*pga* were free from infection. Thus, these findings reflected the PGA impact on *Salmonella* intramacrophage survival and supported the view that heterologous PGA production impairs *Salmonella* survival in orally infected mice.

### PGA production makes *Salmonella* sensitive to bile salts

**Fig 6:**
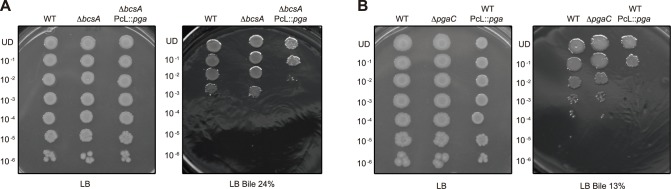
Production of PGA in *Salmonella* and *E*. *coli* greatly increases bile sensitivity. **(A)** Bile sensitivity assay for the wild type, the PGA producing strain, *ΔbcsA* PcL::*pga*, and their corresponding exopolysaccharide minus strain, *ΔbcsA*. Two microliter portions of the appropriate dilutions of each culture were incubated for 24 h at 37°C in an LB plate (left) or an LB plate containing 24% bile bovine (right). **(B)** Bile sensitivity assay for *E*. *coli* MG1655, a MG1655 *pgaC* mutant and MG1655 PcL::*pga* strain that overproduces PGA. Two microliter portions of the appropriate dilutions of each culture were incubated for 24 h at 37°C in an LB plate (left) or an LB plate containing 13% bile bovine (right).

Bile resistance is indispensable for *Salmonella* to colonize the hepatobiliary tract during systemic infection and persist in the gall bladder during chronic infection [60,61] and again, this characteristic represents a major difference between *Enterobacteriaceae* species. Thus, to further examine the consequences of PGA production in the *Salmonella* infection process, we analyzed the ability of *Salmonella* PGA producing cells to cope with the presence of bile. Dilutions from cultures of the wild type, the PGA producing strain, *ΔbcsA* PcL::*pga*, and their corresponding exopolysaccharide minus strain, *ΔbcsA*, were spread on LB plates supplemented with 24% bile bovine. Exposure to bile caused a decrease of ~3 logs in the number of cfu of both the wild type and *ΔbcsA* strain, whereas it provoked a reduction of ~5 logs in the case of the PGA producing strain (Fig 6A). Remarkably, PGA production in *E*. *coli* was also very detrimental for bile survival, since an *E*. *coli* strain producing PGA showed a ~2.5 logs reduction in bile sensitivity compared either with the wild type or with a *pgaC* mutant (Fig 6B and S7 Fig). To determine if the observed bile sensitivity mediated by PGA was common to other membrane active agents, we tested the sensitivity of *Salmonella* and *E*. *coli* PGA producing strains to the anionic detergent SDS. The minimal inhibitory concentration (MIC) of SDS for the *Salmonella* wild type and *ΔbcsA* strains was 17%, whilst it decreased to 15% in the case of *ΔbcsA* PcL::*pga*. On the other hand, the MIC for *E*. *coli* MG1655 and *ΔpgaC* strains was found to be 15%, compared with 7% for the PGA producing strain MG1655 PcL::*pga*. Altogether, these results indicate that PGA causes a significant reduction in bile resistance both in *Salmonella* and *E*. *coli* and suggest that this negative effect on resistance might be generalizable to other detergents.

## Discussion

Acquisition of new genes is considered to be a mechanism to enhance an organism’s ability to colonize a new environment, resist a specific antimicrobial or evade the immune system [40,62]. However, genomic data reveal that gene loss is also a widespread strategy to enhance bacterial fitness [63–68]. There are at least two reasons why bacteria may loss genes during evolution. A gene product or pathway may become superfluous in the new environment. In the absence of purifying selection, the gene accumulates neutral mutations, generating pseudogenes that may be finally removed from the bacterial genome. Alternatively, the product of the gene may be detrimental, triggering selection to optimize bacterial fitness in the new environment. It is well established that *Salmonella* evolution towards virulence has, at least, involved the acquisition by horizontal gene transfer (HGT) of a virulence plasmid and several pathogenicity islands that contain the genes necessary for invasion of intestinal epithelial cells and the systemic phase of infection [69,70]. However, the possibility that adaptation of *Salmonella* to the intracellular environment has occurred through gene loss has rarely been considered [65,71]. McClelland et al. proposed that gene deletion has contributed to genome degradation in *S*. Paratyphi A and Typhi serovars as they specialized to be human restricted variants. Nevertheless, these authors pointed out that the contribution of gene deletion to this evolution is less obvious than that of point mutations (pseudogenes) since the existence of a deletion is sometimes hard to determine [72]. Our work provides evidence that acquisition by *Salmonella* of an arsenal of virulence factors might have been useless in a strain producing PGA, supporting the idea that gene transfer and gene loss are inter-related processes, and that both contribute to the ongoing evolution of pathogenicity [73].

All bacterial species adapted to the mammalian intestine are resistant to the antibacterial activity of bile salts. However, the resistance of *Salmonella enterica* is especially remarkable. During systemic infection, *Salmonella* is able to transit from the liver into the gallbladder, where it can either induce inflammation and acute infection or persist chronically, creating a carrier state [74–76]. Several cell components and mechanisms have been related with *Salmonella* resistance to bile [77,78]. On one hand, different efflux pumps transport bile salts outside the cell decreasing their intracellular concentration [79,80]. On the other, diverse strategies that involve membrane reorganization and provide barriers to reduce bile salts uptake have been described, such as remodeling the lipopolysaccharide (both lipid A and O-antigen), changing the length of the enterobacterial common antigen and reducing the content of the Braun lipoprotein bound to the peptidoglycan, the levels of muropeptides cross-linked by 3–3 peptide bridges and the amount of porins sensitive to bile [81–86]. Our finding that constitutive production of PGA causes bile sensitivity in *S*. Enteritidis suggests an alternative strategy: the removal of compounds (PGA) that render the bacteria susceptible to bile. How PGA causes this effect is presently unclear. PGA represents an unusual bacterial exopolysaccharide, as some GlcNAc residues become deacetylated by the PgaB protein during secretion, providing a positive net charge to the polymer [15,32]. Thus, the presence of PGA may favor the accumulation of anionic bile salts on the bacterial surface.

We showed that constitutive expression of PGA also causes bile sensitivity in *E*. *coli*. These results raise the broader question of why *E*. *coli*, which displays a fair level of bile resistance necessary to grow in the small intestine, still produces PGA. Bile salts are maintained at high concentrations in the duodenum, jejunum, and proximal ileum. In the distal ileum, bile salts are absorbed into the blood-stream, and the majority of bile is recycled back into the small intestine and does not enter the colon [78]. *E*. *coli* resides in the microbiota found in the cecum and colon of humans. Thus, the presence of PGA might be compatible with the bile concentration in the small intestine and not with the concentration in the gallbladder. Alternatively, it is also possible that *E*. *coli* has developed regulatory systems to prevent PGA expression in the small intestine.

The second step of the infection process that is negatively affected by the presence of PGA is the survival and replication in the vacuole within host phagocytic cells. During systemic infection, *Salmonella* survives and replicates in vacuoles within host phagocytic cells where it must overcome the reactive oxygen species produced by macrophages [87]. It has been reported that *Salmonella* needs to repress cellulose production inside the vacuole through the activation of MgtC, which prevents a rise in c-di-GMP [53]. Increased levels of cellulose interfere with replication inside the vacuole and impair virulence in mice. The mechanisms underlying the antivirulence trait of cellulose has not been determined. We have now found that PGA production also hinders *Salmonella* division inside macrophages. Regarding this phenotype, we showed that production of PGA increases the susceptibility to H_2_O_2_ treatment, thus providing a potential mechanism for this attenuation. The notion that PGA is detrimental during infection of mammal cells is supported by studies with *Y*. *pestis* [28]. *Y*. *pestis* forms PGA mediated biofilms below 30°C in the blood-feeding fleas favoring the transmission and invasiveness of the bacteria from fleas to mammals [88]. However, PGA production has to be inhibited in the mammal host over 30°C to allow the development of a lethal infection. This temperature dependent regulation of PGA depends on the tight regulation of the c-di-GMP secondary messenger.

*Salmonella* is an ubiquitous bacterium with a dual intracellular/extracellular lifestyle. Its extracellular life involves survival in the environment, a scenario in which exopolysaccharide-mediated biofilms play an important role, protecting bacteria against environmental threats. Our results indicate that PGA loss provides a fitness advantage when *Salmonella* colonizes the liver, gallbladder or resides inside the macrophages. However, loss of PGA might have negative consequences for survival in the environment unless another compound off the cell wall was able to compensate for PGA absence. Comparative phenotypic analysis between the protection conferred by PGA and cellulose against environmental threats revealed that PGA confers at the most similar benefits than cellulose, indicating that cellulose is sufficient to provide *Salmonella* with protection against environmental stresses and compensate for the loss of PGA function.

Our findings provide a plausible explanation for PGA loss from the *Salmonella* genome during evolution. They also enhance our understanding of the benefits and burdens of a widely used exopolysaccharide to form the bacterial biofilm matrix, highlighting the necessity of additional studies to depict the exact role of PGA at each step of the life cycle. Finally, our study may also encourage microbiologists to turn more attention towards gene loss research as an approach to obtain information about how pathogenic bacteria have adapted to the host.

## Materials and methods

### Phylogenetic analyses

Protein sequences from *E*. *coli* PgaABCD and PhoH were used in a Blastp search against the NCBI non-redundant database accessed in July 2016, using an e-value threshold of 10^−5^ and excluding from the results hits taxonomically assigned to *E*. *coli*. The sequences from the top 500 hits were retrieved for each search and aligned using MUSCLE v 3.8 [89] and then trimmed using trimAl v1.4 [90] (gap-score cut-off 0.9). A Maximum Likelihood phylogenetic reconstruction was performed using phyML v3.0 [91] with the JTT model, setting the number of rate categories to four, and inferring the number of invariant positions and the parameters of the gamma distribution from the data. Branch support was computed using an aLRT (approximate likelihood ratio test) based on a chi-square distribution.

### Ethics statement

Animal studies were performed in accordance with the European Community guiding in the care and use of animals (Directive 2010/63/EU). Protocols were approved by the ethics committee of the Public University of Navarra (Comité de Ética, Experimentación Animal y Bioseguridad of the Universidad Pública de Navarra) (approved protocol PI-004/11). Work was carried out in the animal facility of the Instituto de Agrobiotecnología, Universidad Pública de Navarra. Animals were housed under controlled environmental conditions with food and water ad libitum. Mice were euthanized by CO_2_ inhalation followed by cervical dislocation and all efforts were made to minimize suffering.

### Bacterial strains, plasmids and growth conditions

The strains and plasmids used in this work are described in S1 Table. *Escherichia coli* and S. *enterica* subsp. *enterica* serovar Enteritidis (*S*. Enteritidis) cells were grown in LB broth and on LB agar (Pronadisa) with appropriate antibiotics at the following concentrations: kanamycin (Km), 50 μg ml^-1^; ampicillin (Am), 100 μg ml^-1^; carbenicillin (Cb), 50 μg ml^-1^; chloramphenicol (Cm), 20 μg ml^-1^; and streptomycin (Sm) 500 μg ml^-1^.

### DNA manipulations

Routine DNA manipulations were performed using standard procedures unless otherwise indicated. Plasmid DNA from *E*. *coli* was purified using a Quantum Prep plasmid kit (BioRad). Plasmids were transformed into *E*. *coli* and *S*. Enteritidis by electroporation. Transformants carrying Red helper plasmids were made electro-competent as described [10,92]. Restriction enzymes were purchased from ThermoFisher Scientific and used according to the manufacturer’s instructions. Oligonucleotides were synthesized by StabVida (Caparica—Portugal) and are listed in S2 Table. Phage P22 HT105/1 int-201 [93] was used to carry out transductions between strains according to recommended protocols [94].

### Construction of a collection of strains containing a single GGDEF domain protein

*S*. Enteritidis 3934 ΔXII is a multiple mutant carrying mutations in all genes encoding GGDEF domain proteins [42]. Derivatives of ΔXII containing the following single GGDEF protein encoding gene, namely *adrA*, *yeaJ*, *sen1023*, *yciR*, *yegE*, *yfiN*, *yhdA*, *sen3222*, and *yhjK* were constructed as described [41]. In the case of ΔXII+*sen2484*, ΔXII+*yfeA* and ΔXII+*sen4316* strains, DNA fragments corresponding to the coding sequences of *sen2484*, *yfeA* and *sen4316 genes* were amplified with primer pairs A and D and chromosomal DNA from *S*. Enteritidis 3934 as a template. Amplified fragments were sequenced and cloned into the pKO3blue plasmid that was electroporated into ΔXII. Integration and excision of the plasmid was performed as described [41] in order to obtain the corresponding restored strains.

### Chromosome expression of *adrA* under PcL and P_*phoP*_ promoters

To express *adrA* under the PcL constitutive promoter in *S*. Enteritidis 3934, a PCR generated linear DNA fragment was used as described [95] with some modifications. The Red helper plasmid pKD46 was transformed into *S*. Enteritidis 3934, and transformants were selected on LB agar Am after incubation at 30°C for 24 h. One transformant carrying pKD46 was made electrocompetent as described [10]. A DNA fragment containing a kanamycin resistance gene, the PcL promoter and the RBS sequence of the PcL cassette was generated by PCR using primers *adrA* Km PcL rbs Fw and *adra* Km PcL rbs Rv and chromosomal DNA from strain MG1655 Km PcL-λATT-GFP as template [96]. Electroporation (25 mF, 200 W, 2.5kV) was carried out according to the manufacturer’s instructions (Bio-Rad) using 50 μl of cells and 1 to 5 μg of purified and dialysed (0.025 μm nitrocellulose filters; Millipore) PCR product. Shocked cells were added to 1 ml of LB broth, incubated for 1 h at 28°C and then spread on LB Km agar to select Km^R^ transformants after incubation at 37°C for 24 h. Transformants were then grown on LB Km broth at 44°C for 24 h and incubated overnight on LB Am agar at 28°C to test for loss of the helper plasmid.

To place the *adrA* gene under the control of the *phoP* promoter, a protocol described previously was carried out with some modifications [92]. In a first step, primers Km SceI P_*phoP*_ *adrA* Fw and Km SceI P_*phoP*_ *adrA* Rv, with 60-bp homology extensions, were used to amplify a kanamycin resistance cassette and an I-SceI recognition site from plasmid pWRG717. This DNA was integrated upstream the *adrA* gene via λ Red-mediated recombination using plasmid pWRG730, a temperature-sensitive plasmid for independent inducible expression of the λ Red recombinase and I-SceI endonuclease. After confirming proper insertion of the resistance cassette by colony PCR with primers 01-E and Km SceI P_*phoP*_ *adrA* Rv, a DNA fragment generated by PCR and derived from oligonucleotides P_*phoP*_ *adrA* Fw and P_*phoP*_ *adrA* Rv and *S*. Enteritidis 3934 chromosomal DNA as template, was electroporated into the mutant strain still containing the pWRG730 plasmid. This DNA fragment included the *phoP* promoter and homology regions used for its upstream *adrA* integration. After 1 h of incubation at 28°C, 100 μl of a 10^−2^ dilution was plated on LB agar plates containing 500 ng ml^-1^ anhydrotetracycline, which induced expression of I-SceI endonuclease. After overnight incubation at 28°C, single colonies were purified and successful recombination was checked by monitoring absence of antibiotic resistance, colony PCR with oligonucleotides 01-E and P_*phoP*_ *adrA* Rv, and sequencing of the resulting fragment. Finally, pWRG730 was cured by incubating selected colonies at 44°C.

### Construction of a *Salmonella* strain that constitutively expresses the *pgaABCD* operon from the chromosome

To insert the *pgaABCD* genes from *E*. *coli* K-12 MG1655 into the *S*. Enteritidis 3934 chromosome, the T64B prophage site was chosen [97]. Two DNA fragments, *sb13* AB and *sb13* CD, of ∼500 bp length of the *S*. Enteritidis *sb13* gene, were amplified with primer pairs SmaI *sb13* AB Fw/SphI *sb13* AB Rv and SphI *sb13* CD Fw/SalI *sb13* CD Rv, respectively. The PCR products were cloned into the pJET 1.2 vector (ThermoFisher Scientific) and resulting plasmids were digested with SmaI and SphI enzymes in the case of the AB fragment and SphI and SalI enzymes in the case of the CD fragment. AB and CD fragments were ligated in the same ligation mixture with the pKO3 vector [98] digested with SmaI and SalI enzymes, resulting in plasmid pKO3::*sb13*AD. The pJET::*pga* plasmid constructed in this study was digested with SphI to obtain a DNA fragment containing the *pga* promoter and *pgaABCD* genes. P_*pga*_::*pgaABCD* was ligated with pKO3::*sb13*AD digested with SphI, resulting in pKO3::*sb13*AD-P_*pga*_::*pgaABCD* plasmid. Integration and excision of the plasmid was used as described [98] to obtain WT P_*pga*_::*pgaABCD* strain. Insertion of P_*pga*_::*pgaABCD* into the *sb13* gene was confirmed by PCR using primers *sb13* OK Fw and *pgaA* comp Rv. The ability of this strain to produce PGA was not detectable by Dot Blot, probably because heterologous chromosomal expression of the *pgaABCD* operon under its own promoter was not sufficient to produce evident PGA levels. Thus, a second *Salmonella* strain was generated in order to express *pgaABCD* under the PcL constitutive promoter and in the chromosome. To do so, a 427 bp DNA fragment, namely *sb13* AB_2_, of the *S*. Enteritidis *sb13* gene was amplified with primers BglII *sb13* AB Fw and BamHI sb13 AB Rv, using *S*. Enteritidis 3934 chromosomal DNA as template, and cloned into the pJET 1.2 vector (ThermoFisher Scientific). A second DNA fragment containing the PcLrbs promoter [96] and the first 543 bp of the *pgaA* gene coding sequence was constructed by overlapping PCR, using two separate PCR products. Primers BamHI PcLrbs Fw and sb13 PcL*pga* Rv were used to amplify the PcLrbs promoter, using *E*. *coli* MG1655 Km PcL-λATT-GFP chromosomal DNA as template [96]. Primers PcL *pgaA* Fw and PstI PcL *pgaA* Rv were used to amplify 543 bp of the *pgaA* gene, using *E*. *coli* MG1655 chromosomal DNA as template. These two purified PCR products were mixed, and a second PCR using BamHI PcL rbs Fw and PstI PcL *pgaA* Rv primers was performed to obtain a single DNA fragment, PcLrbs::*pgaA*, that was cloned into the pJET 1.2 vector (ThermoFisher Scientific). Plasmids pJET::sb13AB_2_ and pJET:: PcLrbs::*pgaA* were digested with BglII /BamHI and BamHI/PstI enzymes, respectively, and digestion products were ligated in the same ligation mixture with the pKO3Blue vector [41] digested with BglII and PstI enzymes, resulting in plasmid pKO3Blue::*sb13*AB_2_-PcLrbs::*pgaA* that was electroporated in WT P_*pga*_::*pgaABCD* strain. Integration and excision of the plasmid was used as described [41] to generate Wt PcLrbs::*pgaABCD*. Insertion of PcLrbs::*pgaABCD* into the *sb13* gene was confirmed by PCR using primers *sb13* OK Fw and sb13 PcL *pgaA* Rv. Finally, a *bcsA* mutation was transduced from Δ*bcsA* strain to generate Δ*bcsA*::Cm^R^ PcLrbs::*pgaABCD*, which is hereafter abbreviated as Δ*bcsA* PcL::*pga*.

### Construction of an *E*. *coli* strain that constitutively expresses the *pgaABCD* operon from the chromosome

To express the *pgaABCD* operon under the PcL promoter in *E*. *coli* MG1655, a PCR generated linear DNA fragment and the Red helper plasmid pKD46 were used as described above. Primers used to generate the DNA fragment containing a kanamycin resistance gene, the PcL promoter and the RBS sequence of the PcL cassette were Km PcL rbs *pga* Fw and Km Pcl rbs *pga* Rv.

### Construction of an *E*. *coli* MG1655 Δ*pgaC* mutant

To delete a 500 bp fragment of the *pgaC* gene in *E*. *coli* MG1655, and as a consequence suppress PGA production in *E*.*coli* [15], a protocol described previously was carried out with some modifications [92]. First, primers *pgaC* Km SceI Fw and *pgaC* Km SceI Rv, with 60-bp homology extensions, were used to amplify a kanamycin resistance cassette and an I-SceI recognition site from plasmid pWRG717. This DNA was integrated in the *pgaC* gene using plasmid pWRG730 plasmid and integration was confirmed by colony PCR with primers *pgaC* Km SceI Fw and *pgaD* Rv. Phosphorylated 80-mer double-stranded DNA derived from oligonucleotides Δ*pgaC* Fw and Δ*pgaC* Rv was electroporated into the mutant strain still containing the pWRG730 plasmid. After 1 h of incubation at 28°C, 100 μl of a 10^−2^ dilution was plated on LB agar plates containing 500 ng ml^-1^ anhydrotetracycline, which induced expression of I-SceI endonuclease. After overnight incubation at 28°C, single colonies were purified, and successful recombination was checked by monitoring absence of antibiotic resistance and colony PCR with oligonucleotides Δ*pgaC* Fw and *pgaD* Rv. Finally, pWRG730 was cured by incubating selected colonies at 44°C.

### PGA quantification

PGA exopolysaccharide levels were quantified as previously described [14] with minor modifications. Briefly, cultures in 5 ml LB or LB Cb broth of the strains tested were adjusted to the same number of cells and centrifuged at 18,000 x g for 5 min. Pellets were resuspended in 50 μl of 0.5 M EDTA (pH 8.0) and suspensions were incubated for 5 min at 100°C and centrifuged at 18,000 x g for 5 min. Each supernatant (40 μl) was incubated with 10 μl of proteinase K (20 mg ml^-1^) (Sigma) for 30 min at 37°C. After the addition of 10 μl of Tris-buffered saline (20 mM Tris-HCl, 150 mM NaCl [pH 7.4]) containing 0.01% bromophenol blue, 5 μl were spotted on a nitrocellulose membrane using a Bio-Dot microfiltration apparatus (Bio-Rad). The membrane was blocked overnight with 5% skimmed milk in phosphate-buffered saline (PBS) with 0.1% Tween 20, and incubated for 2 h with specific anti-PNAG antibodies diluted 1:10,000 [25]. Bound antibodies were detected with peroxidase-conjugated goat anti-rabbit immunoglobulin G antibodies (Jackson ImmunoResearch Laboratories, Inc., West- grove, PA) diluted 1:10,000 and developed using the SuperSignal West Pico Chemiluminescent Substrate (ThermoFisher Scientific). All extracts assayed in a particular experiment were analyzed on the same membrane. Images obtained in a GBox Chemi HR16 system (Syngene) were cut and put together to assemble horizontal figures showing PGA quantification.

### Biofilm formation

The cellulose mediated biofilm formed in glass tubes on standing rich cultures was examined visually after growth in 5 ml of LB broth at room temperature for 72 h [10]. The PGA mediated biofilm was visualized after growth in LB broth at 28°C in an orbital shaker (250 r.p.m) for 16 h [43]. Macrocolony biofilms on the surface of LB agar plates were formed after spotting 50 μl drops of overnight liquid cultures and incubating at 28°C for 48 hours [99].

### Scanning electron microscopy

For scanning electron microscopy bacterial strains were grown under biofilm forming conditions. Growth medium was removed and bacterial cells were fixed by adding a fixation solution (1.3% glutaraldehyde, 0.07M cacodylate buffer and 0.05% rhutenium red). Samples were then washed in and post-fixed by incubation with 2% osmium tetroxide for 1 h. Bacteria were then fully dehydrated in a graded series of ethanol solutions and dried in hexamethyldisilazane (HMDS, Sigma). Finally, samples were coated with 40 Å platinum, using a GATAN PECS 682 apparatus (Pleasanton, CA), before observation under a Zeiss Ultra plus FEG-SEM scanning electron microscope (Oberkochen, Germany) (Laboratoire de Biologie Cellulaire et Microscopie Electronique, UFR Médecine (Tours, France)).

### Hydrophobicity assay

Macrocolony biofilms were formed on the surface of LB or LB Cb agar plates as described above, and a 10 μl water droplet stained with red food colouring was placed on the biofilm to show the hydrophobicity exhibited by the structure [48].

### Sodium hypochlorite treatment of macrocolony biofilms

To perform sodium hypochlorite survival analyses, a protocol described previously was carried out with some modifications [10]. Macrocolony biofilms were formed on sterile polymer membrane filters (diameter 47 mm; Millipore) resting on LB agar or LB agar Cb media for 48h at 28°C. Filters were then transferred to an empty petri dish and macrocolonies were treated with 10 ml PBS containing 200 p.p.m. sodium hypochlorite for 40 min at 37°C. Control samples were incubated with 10 ml of PBS. Macrocolonies were harvested with a bent tip and bacteria were washed in PBS three times and suspended in 5 ml of PBS. After vortexing and sonicating (30 sec; potency 3; Branson sonifier 250; microtip), bacteria were enumerated by viable plate counts.

### UV light treatment

Bacterial strains were grown in LB Cb broth at 28°C in an orbital shaker (200 r.p.m) for 16 h. After sonication (30 sec; potency 3; Branson sonifier 250; microtip), the OD_600nm_ was adjusted to 1 and serial dilutions were plated on four plates of N media agar supplemented with Cb [53]. After 24h of growth at 28°C, two plates were irradiated with UV light for 5 min. All plates were then incubated at 28°C for 48h and the numbers of surviving bacteria were counted. Results are shown as % survival relative to non-irradiated samples. Experiments were conducted in triplicate.

In order to incubate all strains on the same plates, strains *ΔbcsA* PcL::*adrA* and WT PcL::*adrA* were transformed with a pJET empty plasmid.

### Heavy metal resistance assay

Macrocolony biofilms on sterile polymer membrane filters (diameter 47 mm; Millipore) resting on LB agar or LB agar Cb media were formed after spotting 5 μl drops of overnight liquid cultures and incubating at 28°C for 48 hours. Filters were then transferred to an empty petri dish and macrocolonies were treated with 10 ml of 0.5 mM CdCl_2_ for 3 h at 28°C. Control samples were incubated with 10 ml of PBS. Macrocolonies were harvested with a bent tip and bacteria were washed in water three times and suspended in 5 ml of PBS. After vortexing and sonicating (30 sec; potency 3; Branson sonifier 250; microtip), bacteria were enumerated by viable plate counts.

### Susceptibility of biofilms to phage infection

Overnight cultures in LB or LB Cb broth were sonicated (30 sec; potency of 3; Branson sonifier 250; microtip) and the OD_600nm_ was adjusted to 1. Sterile polymer membrane filters (diameter 47 mm; Millipore) were placed on LB or LB Cb agar plates and seeded with a 50 μl drop of each bacterial suspension. Plates were inverted and incubated at 28°C for 48 h to allow macrocolony biofilm formation on top of the filters, that were then transferred to an empty petri dish and treated with a P22 phage lysate generated from the streptomycin resistant strain *S*. Typhimurium SL1344. After 1h of incubation at 37°C, the entire content of the plates was collected, washed in PBS and plated on LB Sm agar. The number of streptomycin resistant cfu were indicative of transduction efficiency. Experiments were conducted in triplicate.

### Macrophage survival assay

Macrophage survival assay was conducted essentially as described [53] with some modifications. The murine macrophage cell line RAW 264.7 was propagated in Dulbecco’s modified Eagle’s medium (DMEM) (Gibco) supplemented with 10% fetal bovine serum (Invitrogen) and 1% Penicillin/Streptomycin/Glutamine (Gibco). Macrophages were seeded at a density of 2 x 10^5^ cells per well in 24-well plates 24 h prior to infection. *Salmonella* overnight cultures grown at 37°C in LB broth were sonicated (30 sec; potency 3; Branson sonifier 250; microtip) and diluted 1:100 in LB broth. Cultures were incubated at 37°C in an orbital shaker (200 r.p.m) to an OD_600nm_ of 1 and the suspension was sonicated again and washed twice with DMEM deprived of serum. Macrophages were then infected with *Salmonella* strains at a multiplicity of infection of approximately 10:1 and plates were centrifuged at 1000 r.p.m. for 10 minutes at room temperature. After 20 min of phagocytosis, monolayers were washed twice with PBS and treated with gentamicin (100 μg ml^-1^) for 1 h. To estimate phagocytosed bacteria, samples were then washed three times with sterile PBS and macrophages were lyzed with 1% (vol/vol) Triton X-100-PBS to release intracellular bacteria that were counted by plating 25 μl of serial dilutions onto LB plates. To assess bacterial survival, medium was replaced by DMEM supplemented with 10% FBS and 12 μg ml^-1^ gentamycin and the cells were incubated at 37°C. After 18 h of infection, wells were washed twice with PBS and were treated with Triton X-100 as indicated above. The percentage survival was obtained by dividing the number of bacteria recovered after 18 h by the number of phagocytosed bacteria and multiplying by 100. At each stage when infected cells were lysed, the number of viable cells in duplicate monolayers infected with each strain was assessed by 0.4% trypan blue exclusion and counting viable cells. No difference in viability was noted between cells infected with the different strains. Experiments were done in triplicate on three independent occasions.

### Polymyxin B resistance assay

One-hour polymyxin susceptibility assays were performed as described [55]. Polymyxin B Sulfate (Sigma) was used at a final concentration of 2.5 μg ml^-1^. Data are presented as survival percentage relative to samples incubated in LB without polymyxin. Experiments were conducted in triplicate.

### Hydrogen peroxide sensitivity assay

Sensitivity to hydrogen peroxide was tested as previously described [57] with minor modifications. Briefly, overnight cultures were subcultured at 1/100 in 5 ml LB containing either no or 1 mM H_2_O_2_ (Merk). Replica cultures were used for each time point. Cultures were grown at 37°C with aeration and collected hourly. LB broth contains ∼30–40 μM Mg^2+^ [101], which activates the *phoP* promoter, thus, leading to *adrA* expression. After sonication (30 sec; potency 3; Branson sonifier 250; microtip) the number of surviving bacteria were counted by plating serial dilutions onto LB plates. Experiments were performed on three separate occasions.

### Ileal loop co-infection experiment

In order to differentiate strains in all mice competitive infections performed, the wild type and *ΔbcsA* PcL::*pga* strains were made streptomycin (Sm) resistant through P22 phage transduction of the *aadA* gene from the natural streptomycin resistant strain *S*. Typhimurium SL1344 [102].

To compare the *in vivo* interaction of *Salmonella* strains with murine intestinal epithelial cells, the ligated ileal loop co-infection model was used as described previously [10,100]. Strains were incubated on LB agar for 48 hours at room temperature, suspended in PBS and sonicated (30 sec; potency 3; Branson sonifier 250; microtip) prior to infection. Competitive index (CI) was defined as the log_10_ of the ratio of the exopolysaccharide producing strain to control strain recovered (Output) divided by the ratio of the exopolysaccharide producing strain to control strain present in the inoculum (Input). A CI > 0 indicates the exopolysaccharide producing strain with a colonization advantage compared to the control and a CI < 0 indicates the exopolysaccharide producing strain with a colonization disadvantage over the control.

### Colonization experiments

Colonization experiments were carried out with 8-week-old female BALB/c mice (Charles River Laboratories). Mice were acclimated for 7 days after arrival before the experiments were started in the animal facility of the Instituto de Agrobiotecnología, Universidad Pública de Navarra. Food and water were removed, twelve and two hours respectively, before the administration of bacterial suspension. Mice were prefed with 20 μl of 10% sodium bicarbonate 30 min before bacterial inoculation. Water and food were again supplied right after inoculation.

Strains were incubated on LB agar for 48 hours at room temperature, suspended in PBS and sonicated (30 sec; potency 3; Branson sonifier 250; microtip) prior to infection. Mice were inoculated intragastrically with 100 μl of bacterial suspensions. In the case of coinfection experiments, the total bacteria inoculum was 2 x 10^8^ cfu of combined polysaccharide producing strain and *ΔbcsA* strain at a ratio of 1:1. In the case of individual infections, inoculum was 1 x 10^8^ cfu of the strain analysed. The cfu of each strain in the inoculum (input) were quantified by plating dilution series on LB agar supplemented with chloramphenicol and LB agar supplemented with streptomycin to distinguish between strains. Over the course of infection, mice were examined twice per day and a final disease score was given to each mouse according to clinical signs observed as follows. No clinical signs (0); mild clinical signs: ruffled fur (1); moderate clinical signs: ruffled fur plus, lethargy, hunched posture and decreased activity (2); severe clinical signs: paresis, paralysis, tremor, shivers, ataxia, rigidity (3). When evident signs of disease (score 2 to 3) were observed, mice were euthanized by CO_2_ inhalation followed by cervical dislocation. Then, dilution series of liver, spleen and gallbladder lysates were plated on LB agar for enumeration of cfu (output), using antibiotic resistance to differentiate strains. Values for CI were calculated as described above.

### Bile and SDS sensitivity assays

Bile bovine sensitivity assay was performed as described [82] with minor modifications. Bacterial strains were grown in LB broth at 28°C for 48 h in an orbital shaker (200 r.p.m). Two microliter portions of serial dilutions were incubated for 24 h at 37°C in LB agar plates containing either no or bile bovine (Sigma). The bile concentration used was 24% or 13% when assessing *Salmonella* and *E*. *coli* bile sensitivity, respectively.

To carry out the SDS MIC analysis, bacterial strains were grown in LB broth at 28°C for 48 h in an orbital shaker (200 r.p.m) and diluted such that samples of 2x10^3^ CFU/ml were subjected to various concentrations of SDS in polypropylene microtiter plates (ThermoFisher Scientific). The plates were incubated overnight at 37°C under nonaerated conditions and the wells of the plate were visually analyzed to determine the MICs.

### Statistical analyses

All statistical analyses were performed in GraphPad Prism 5.01. Sodium hypochlorite survival, UV light irradiation data, heavy metals resistance, susceptibility of biofilms to phage infection, macrophage survival and Polymyxin B resistance analyses were analysed by the Mann-Whitney *U* test. A two-way analysis of variance combined with the Bonferroni test was used to analyse statistical significance in hydrogen peroxide sensitivity assays. A nonparametric Mann-Whitney *U* test and an unpaired Student’s t test were used to assess significant differences in individual colonization or coinfection experiments, respectively.

## Supporting information

S1 Table Strains and plasmids used in this study. (PDF)

S2 Table Oligonucleotides used in this study. (PDF)

S1 Fig Gene neighborhood analysis for *pgaA*, as provided in the STRING database [36]. Results for *Enterobacteriaceae* genomes are fully displayed, while those from other groups are presented in a collapsed mode.
(EPS)

S2 Fig Analysis of PGA synthesis dependence on c-di-GMP. Quantification of PGA exopolysaccharide production by dot blot. Serial dilutions (1/10) of the samples were spotted onto nitrocellulose membranes and PGA production was detected with specific anti PIA/PNAG antibodies. UD; undiluted sample. **(A)** Dot blot analysis of the PGA accumulated by *S*. Enteritidis wild type, WT PcL::*adrA* and their corresponding transformed strains with plasmid pJET::*pga* throughout 72 hours of incubation under *Salmonella* biofilm forming conditions, that is incubation in LB or LB Cb broth, at room temperature, under static conditions. **(B)** Dot blot analysis of the PGA accumulated by ΔXII and its twelve derivative strains, in which c-di-GMP synthesis is provided by a unique GGDEF domain protein. The wild type strain is shown as a control. All strains carried plasmid pJET::*pga* and were grown for 72 hours in LB Cb broth, at room temperature, under static conditions. Seven genes that are shown in a black box encode predicted or demonstrated c-di-GMP synthases. Five of them, namely AdrA, YedQ, YegE, YfiN and SEN4316, were able to induce PGA synthesis. The rest of the proteins, namely YciR, YfeA, YhdA, YhjK and SEN2484, although harboring a GGDEF domain, are predicted or demonstrated phosphodiesterases (c-di-GMP degrading enzymes) or proteins with no c-di-GMP metabolic activity, and therefore, do not elicit PGA synthesis.
(EPS)

S3 Fig Hydrophobicity analysis of macrocolony biofilms formed by WT PcL::*adrA* and *ΔbcsA* PcL::*adrA* pJET::*pga*. **(A)** The cellulose overproducing strain, WT PcL::*adrA*, the PGA overproducing strain, *ΔbcsA* PcL::*adrA* pJET::*pga* and the control strain, *ΔbcsA* PcL::*adrA*, were incubated on LB agar or LB agar Cb media for 48h at 28°C. A bent tip was used to visualize the macrocolony appearance. The cellulose mediated macrocolony looked like a pellicle that was very difficult to disrupt whilst the PGA mediated macrocolony appeared much more mucoid. **(B)** Macrocolony biofilms were generated and a 10 μl water droplet stained with red food coloring was placed on each macrocolony, showing the hydrophobicity exhibited by the structure.
(EPS)

S4 Fig Strains WT P_*phoP*_::*adrA* and *ΔbcsA* P_phoP_::*adrA* PcL::*pga* produce cellulose and PGA, respectively, in a *phoP* dependent fashion. A cellulose based biofilm is produced by WT P_*phoP*_::*adrA* after overnight growth in low-Mg^2+^ liquid medium (top panel). An aggregate of *ΔbcsA* P_*phoP*_::*adrA* PcL::*pga* bacteria can be observed at the bottom of the tube as Mg^2+^ concentrations decrease, indicative of PGA synthesis (low panel). Such aggregation correlates with increased PGA production detected by dot blot, using specific anti PIA/PNAG antibodies. Strain *ΔbcsA* P_*phoP*_::*adrA* that produces neither cellulose nor PGA shows a planktonic phenotype.
(EPS)

S5 Fig Synthesis of PGA by a *Salmonella* strain that constitutively expresses the *pgaABCD* operon from the chromosome. Comparison of the PGA accumulated by WT PcL::*pga* and WT pJET::*pga*, expressing the *pgaABCD* operon from the chromosome or from a plasmid, respectively, after 24 hours of growth at 37°C in LB or LB Cb media. Serial dilutions (1/10) of the samples were spotted onto nitrocellulose membranes and PGA production was detected with specific anti PIA/PNAG antibodies. UD; undiluted sample.
(EPS)

S6 Fig Fitness of *ΔbcsA* versus the wild type or *ΔbcsA* PcL::*pga* strains. The competitive fitness of *ΔbcsA* in co-culture with either the wild type or *ΔbcsA* PcL::*pga* was determined by combining the two strains in LB broth, incubating at 37°C and enumerating each strain over time.
(EPS)

S7 Fig PGA is overproduced by *E*. *coli* MG1655 when the *pgaABCD* operon is expressed from a constitutive promoter. Dot blot analysis of the PGA accumulated by *E*. *coli* MG1655, a *pgaC* mutant and a derivative of *E*. *coli* MG1655 in which the *pga*ABCD operon was placed under the control of the constitutive promoter PcL, after 24 hours of growth at 37°C in LB media. Serial dilutions (1/10) of the samples were spotted onto nitrocellulose membranes and PGA production was detected with specific anti PIA/PNAG antibodies. UD; undiluted sample. Note that, as it has already been described [32], the parental *E*. *coli* MG1655 strain exhibited an extremely weak reaction with the antibody because of the posttransciptional repression of PGA production by the CsrA protein [103].
(EPS)

S1 Dataset Phylogenetic trees of the analyzed proteins. First column indicates the protein name, and the second column is the phylogenetic tree in standard newick format. Labels indicate species names, as retrieved from NCBI, followed by an underscore separator and the gene identifier.
(PDF)
