# Supplementary material for: Lack of the PGA exopolysaccharide in Salmonella as an adaptive trait for survival in the host
Source: PLoS Genet. 2017 May 24;13(5):e1006816. doi: 10.1371/journal.pgen.1006816 (PMC5464674; doi:10.1371/journal.pgen.1006816)
Supplement: S1 Table — (PDF) [file pgen.1006816.s001.pdf]

S1 Table. Strains and plasmids used in this study.

Strain or plasmid	Relevant characteristics	MIC[a]	Reference or source[b]
Strains			
***S.* Enteritidis**			
3934	Wild-type clinical isolate	54	[1]
ΔXII	3934 Δ*adrA* Δ*sen1023* *yeaJ::*Tc^R Δ*yciR* Δ*yegE* Δ*yfiN* Δ*yhda* Δ*sen3222* Δ*yhjK* Δ*sen2484* *yfeA::*Km^R Δ*sen4316*	3664	[2]
Δ*bcsA*	3934 Δ*bcsA*::Cm^R	3702	[2]
WT PcL::*adrA*	3934 Km^R PcL rbs::*adrA*	4397	This study
Δ*bcsA* PcL::*adrA*	3934 Δ*bcsA*::Cm^R Km^R PcL rbs::*adrA*	5583	This study
WT P_{phoP}::*adrA*	3934 P_{phoP}::*adrA*	5624	This study
Δ*bcsA* P_{phoP}::*adrA*	3934 Δ*bcsA*::Cm^R P_{phoP}::*adrA*	5626	This study
Δ*bcsA* P_{phoP}::*adrA* PcL::*pga*	Δ*bcsA*::Cm^R P_{phoP}::*adrA* PcL rbs::*pga*	5860	This study
Δ*bcsA* PcL::*pga*	3934 Δ*bcsA*::Cm^R PcL rbs::*pgaABCD*	5735	This study
ΔXII + *adrA*	3934 Δ*sen1023* *yeaJ::*Tc^R Δ*yciR* Δ*yegE* Δ*yfiN* Δ*yhda* Δ*sen3222* Δ*yhjK* Δ*sen2484* *yfeA::*Km^R Δ*sen4316*	2785	This study
ΔXII + *yeaJ*	3934 Δ*adrA* Δ*sen1023* Δ*yciR* Δ*yegE* Δ*yfiN* Δ*yhda* Δ*sen3222* Δ*yhjK* Δ*sen2484* *yfeA::*Km^R Δ*sen4316*	2786	This study
ΔXII + *yciR*	3934 Δ*adrA* Δ*sen1023* *yeaJ::*Tc^R Δ*yegE* Δ*yfiN* Δ*yhda* Δ*sen3222* Δ*yhjK* Δ*sen2484* *yfeA::*Km^R Δ*sen4316*	2787	This study
ΔXII + *sen1023*	3934 Δ*adrA* *yeaJ::*Tc^R Δ*yciR* Δ*yegE* Δ*yfiN* Δ*yhda* Δ*sen3222* Δ*yhjK* Δ*sen2484* *yfeA::*Km^R Δ*sen4316*	2788	This study
ΔXII + *yegE*	3934 Δ*adrA* Δ*sen1023* *yeaJ::*Tc^R Δ*yciR* Δ*yfiN* Δ*yhda* Δ*sen3222* Δ*yhjK* Δ*sen2484* *yfeA::*Km^R Δ*sen4316*	2789	This study
ΔXII + *yfeA*	3934 Δ*adrA* Δ*sen1023* *yeaJ::*Tc^R Δ*yciR* Δ*yegE* Δ*yfiN* Δ*yhda* Δ*sen3222* Δ*yhjK* Δ*sen2484* Δ*sen4316*	2790	This study
ΔXII + *yfiN*	3934 Δ*adrA* Δ*sen1023* *yeaJ::*Tc^R Δ*yciR* Δ*yegE* Δ*yhda* Δ*sen3222* Δ*yhjK* Δ*sen2484* *yfeA::*Km^R Δ*sen4316*	2791	This study

ΔXII + *yhdA*	3934 Δ*adrA* Δ*sen1023* *yeaJ::*Tc^R Δ*yciR* Δ*yegE* Δ*yfiN* Δ*sen3222* Δ*yhjK* Δ*sen2484* *yfeA::*Km^R Δ*sen4316*	2792	This study
ΔXII + *sen3322*	3934 Δ*adrA* Δ*sen1023* *yeaJ::*Tc^R Δ*yciR* Δ*yegE* Δ*yfiN* Δ*yhda* Δ*yhjK* Δ*sen2484* *yfeA::*Km^R Δ*sen4316*	2793	This study
ΔXII + *yhjK*	3934 Δ*adrA* Δ*sen1023* *yeaJ::*Tc^R Δ*yciR* Δ*yegE* Δ*yfiN* Δ*yhda* Δ*sen3222* Δ*sen2484* *yfeA::*Km^R Δ*sen4316*	2794	This study
ΔXII + *sen4316*	3934 Δ*adrA* Δ*sen1023* *yeaJ::*Tc^R Δ*yciR* Δ*yegE* Δ*yfiN* Δ*yhda* Δ*sen3222* Δ*yhjK* Δ*sen2484* *yfeA::*Km^R	3831	This study
ΔXII + *sen2484*	3934 Δ*adrA* Δ*sen1023* *yeaJ::*Tc^R Δ*yciR* Δ*yegE* Δ*yfiN* Δ*yhda* Δ*sen3222* Δ*yhjK* *yfeA::*Km^R Δ*sen4316*	2795	This study

E.coli

XL1Blue	*recA1* *endA1* *gyrA96* *thi-1* *hsdr*17 *supE*44 *rel*A1 *lac* [F′*proAB* *lacI*^{q} Z Δ*M15* Tn10 (Tc^R)]	797	Stratagene
K-12 MG1555	Wild type commensal strain	5359	Provided by J. M. Ghigo
MG1655 Km PcL-λATT-GFP	MG1655 Km^R PcL-λATT-GFP	3023	[3]
MG1655 Δ*pgaC*	MG1555 Δ*pgaC*	5487	This study
MG1655 PcL::*pga*	MG1555 Km^R PcL rbs::*pgaABCD*	5732	This study

Plasmids

pJET 1.2	Cloning vector Am^R		Thermo Scientific
pKD46	Temperature-sensitive plasmid containing λ red recombinase genes under the control of an arabinose-inducible promoter; Am^R		[4]
pWRG717	Plasmid with I-SceI recognition site, Km^R. Use for kanamycin cassette amplification in rapid mutagenesis.		Provided by R. G. Gerlach
pWRG730	Plasmid with I-SceI endonuclease under control of tetracycline-inducible promoter (P_{tetA}), temperature-sensitive, Cm^R		Provided by R. G. Gerlach
pKO3	Cm^R vector for recombination experiments		[5]

pKO3Blue	Cm^R, derivative of pKO3 carrying the pMAD lacZ gene under the control of the P*clpB* promoter.	[6]
pJET::*pga*	pJET 1.2 containing the *pgaABCD* operon of *E.coli* MG1655	This study
pKO3::*sb13*AD	pKO3 containing fragments AB and CD from the *sb13* gene of T64B phage.	This study
pKO3::*sb13*AD-P_{pga}::*pgaABCD*	pKO3 containing fragments AB and CD from the *sb13* gene of T64B phage and the *pgaABCD* operon of *E.coli* MG1655 under its own promoter	This study
pKO3Blue::*sb13*AB_2-PcLrbs::*pgaA*	pKO3Blue containing fragment AB_2 from the *sb13* gene of T64B phage and the *pgaA* gene under the PcLrbs promoter	This study
pCN40	Vector encoding ampicillin resistance	[7]

a. Number of each strain in the culture collection of the Laboratory of Microbial Pathogenesis, Navarrabiomed-Universidad Pública de Navarra

b. References

1. Solano C, Sesma B, Alvarez M, Humphrey TJ, Gamazo C, Thorns CJ. Discrimination of strains of *Salmonella enteritidis* with differing levels of virulence by an in vitro glass adherence test. J Clin Microbiol. 1998;36: 674–678.

2. Zorraquino V, Garcia B, Latasa C, Echeverz M, Toledo-Arana A, Valle J, et al. Coordinated cyclic-di-GMP repression of *Salmonella* motility through YcgR and cellulose. J Bacteriol. 2013;195: 417–428. doi:10.1128/JB.01789-12

3. Da Re S, Le Quere B, Ghigo JM, Beloin C. Tight modulation of *Escherichia coli* bacterial biofilm formation through controlled expression of adhesion factors. Appl Environ Microbiol. 2007;73: 3391–3403. doi:10.1128/AEM.02625-06

4. Datsenko KA, Wanner BL. One-step inactivation of chromosomal genes in *Escherichia coli* K-12 using PCR products. Proc Natl Acad Sci USA. 2000;97: 6640–6645. doi:10.1073/pnas.120163297

5. Link AJ, Phillips D, Church GM. Methods for generating precise deletions and insertions in the genome of wild-type *Escherichia coli*: application to open reading frame characterization. J Bacteriol. 1997;179: 6228–6237. doi: 10.1128/jb.179.20.6228-6237.1997

6. Solano C, García B, Latasa C, Toledo-Arana A, Zorraquino V, Valle J, et al. Genetic reductionist approach for dissecting individual roles of GGDEF proteins within the c-di-GMP signaling network in *Salmonella*. Proc Natl Acad Sci USA. 2009;106: 7997–8002. doi:10.1073/pnas.0812573106

7. Charpentier E, Anton AI, Barry P, Alfonso B, Fang Y, Novick RP. Novel Cassette-Based Shuttle Vector System for Gram-Positive Bacteria. Appl Environ Microbiol. 2004;70: 6076–6085. doi:10.1128/AEM.70.10.6076-6085.2004
