# Supplementary material for: Lack of the PGA exopolysaccharide in Salmonella as an adaptive trait for survival in the host
Source: PLoS Genet. 2017 May 24;13(5):e1006816. doi: 10.1371/journal.pgen.1006816 (PMC5464674; doi:10.1371/journal.pgen.1006816)
Supplement: S2 Table — (PDF) [file pgen.1006816.s002.pdf]

Supporting Table 2. Oligonucleotides used in this study.

Primer	Sequence 5´→3´
**Cloning and sequencing of *pgaABCD* and *pgaABCD-ycdT* genes into the pJET vector**	
pgaA Fw	**GCATGC**CCGAAATCATGCATCGGAATTTACTGATT
pgaD Rv	**GCATGC**TTATGCCCGGACTAGCGCTT
pgaA sec Fw	CCGCACTGCCCAGTACCA
pgaB sec Fw	GTGCGCGCGTAAAAGCCAT
pgaC sec Fw	GCCCTGGCAGAAGTGGGTTA
***adrA* chromosome expression under the PcL promoter**	
adrA Km PcL rbs Fw	CCGAAATTTCATACTTCCTCCATGCGCCCTGTTTCTATAATTTGG GAAAATTGTTTCTAATTCGCTCAAGTTAGTAATTCTCAC[a]
adrA Km PcL rbs Rv	TACCGCCTGTTCAACCGCTTTTCGGTAAAAATTTTCATCATTCAT TATTTTTGGGAACATGCGGTACCTTTCTCCTCTTTAATG[b]
***adrA* chromosome expression under the *phoP* promoter**	
Km SceI P_{phoP} *adrA* Fw	GTAGCAAGTTTATGAGCGCCTGCCTGAAAAAAGCGGCGTAGTG CTATCGGGTGAGCCCTGAGGTTTTCCCAGTCACGAC[c]
Km SceI P_{phoP} *adrA* Rv	TACCGCCTGTTCAACCGCTTTTCGGTAAAAATTTTCATCATTCAT TATTTTTGGGAACATTGCTTCCGGCTCGTATGTTG[c]
P_{phoP} *adrA* Fw	GTAGCAAGTTTATGAGCGCCTGCCTGAAAAAAGCGGCGTAGTG CTATCGGGTGAGCCCTGACTATTTGTCTGGTTTATTAACTG[d]
P_{phoP} adrA Rv	TACCGCCTGTTCAACCGCTTTTCGGTAAAAATTTTCATCATTCAT TATTTTTGGGAACATCTCGTCTCCCTTGTGTTAACAATAAGAAC[d]
01-E	CACAGTTGTTATAACGTTAC
***pgaABCD* expression from the *Salmonella* chromosome**	
SmaI *sb13* AB Fw	**CCCGGG**AACTGTATGTCATTGCCGTA
SpHI *sb13* AB Rv	**GCATGC**CCGATATAATCGAACGGCT
SpHI *sb13* CD Fw	**GCATGC**AGACGCCTGCTGATGAACT
SalI *sb13* CD Rv	**GTCGAC**TGCAGACGGAACTGGTTAA
BglII *sb13* AB Fw	**AGATCT**AACTGTATGTCATTGCCGTAC
BamHI *sb13* AB Rv	**GGATCC**ATAAAACGAAAGGCCCAGTCTTTCGACTGAGCCTTTC GTTTTACCGATATAATCGAACGGCTCAT

BamHI PcLrbs Fw	**GGATCC**AAATCTATCACCGCAAGGGA
sb13 PcL*pgaA* Rv	GCTACTTGAATACATGCGGTACCTTTCTCCTCTTT
PcL *pgaA* Fw	AAAGAGGAGAAAGGTACCGCATGTATTCAAGTAGC
PstI PcL *pgaA* Rv	**CTGCAG**CCCCGCCAGTTTATAGATAT
sb13 OK Fw	ATCGGTTGATTATGCCCGTCA
pgaA comp Rv	CCATTTGGTTTTCGGGCACC

***pgaABCD* operon expression in *E. coli* under the PcL promoter**

Km Pcl rbs *pga* Fw	CATTAGGAATAACAATTAAATCCGTGAGTGCCGTAGCGCAGCC TTTCATCAGGACTTTCGTTCGCTCAAGTTAGTAATTCTCAC[a]
Km Pcl rbs *pga* Rv	AGTAAGAAGTTTCAAAGCCCATTTGGTTTTCGGGCACCTTTTTC TGCTACTTGAATACATGCGGTACCTTTCTCCTCTTTAATG[b]

Mutation of the *pgaC* gene in *E. coli*

pgaC Km SceI Fw	TAGACCTTATTCGTCCTGAGTTTTCAACAGCCTGGTATCCGAAA AATGATTAATCGCATCAGGTTTTCCCAGTCACGAC[c]
pgaC Km SceI Rv	TACCGGTTACGGCACCCACACGCGGGTTGTACAACATCGGTTCC ACAATATATGCCGCCTGCTTCCGGCTCGTATGTTG[c]
Δ*pgaC*_Fw	TTTTCAACAGCCTGGTATCCGAAAAATGATTAATCGCATCGGCG GCATATATTGTGGAACCGATGTTGTACAACCCGCGT
Δ*pgaC*_Rv	ACGCGGGTTGTACAACATCGGTTCCACAATATATGCCGCCGATG CGATTAATCATTTTTCGGATACCAGGCTGTTGAAAA

Construction of a collection of strains, derivative of ΔXII containing a single GGDEF domain protein

01-A	**GCGGCCGC**TGCCAGTGTAACTGTGGA[e]
01-D	**AGATCT**CTGGGACACGACCGTAA[e]
02-H	**GCGGCCGC**ATGAATTTGCATCATAAAGCG[e]
02-D	**AGATCT**GGCGATGCGCAGATAGT[e]
03-A	**GCGGCCGC**GATATCACCCAACAAATG[e]
03-D	**AGATCT**CAGATACGCCGGTAATTTT[e]
04-A	**GCGGCCGC**GGAATTGTCGTACACGGT[e]
04-D	**AGATCT**CTCACAACGAAATCCGCC[e]
05-A	**GCGGCCGC**ACCGGTAATTCAATCGCC[e]

05-D	**AGATCT**GTTTGAACAGGGCGTGC[e]
06-A2	**GCGGCCGC**CGTCATCCGTTCCTTGAACATAACGCGTCATCCGT TCCTTGAACATAACG[e]
06-D2	**AGATCT**CTGGGTTACATCTTCTACCCGGTCCTGGGTTACATCTT CTACCCGGTC[e]
07-A	**GCGGCCGC**GACGATATGGCAAAATAATG[e]
07-D	**AGATCT**AGCAACTTGAACAAGAGCA[e]
08-A	**GCGGCCGC**CACAGCATGGCGGTAAAA[e]
08-D	**AGATCT**GATATTGCCCGGCGTAC[e]
09-A	**GCGGCCGC**AGTTTCACCACAGGCGC[e]
09-D	**AGATCT**TTGAGAATAAAACGCAGTTG[e]
10-A	**GCGGCCGC**TATAGCCCGCAGGAATAC[e]
10-D	**AGATCT**ATCGAGCGTTGCCGGAT[e]
11-A	**GCGGCCGC**GTAAGATAACTGTGCGAAG[e]
11-D	**AGATCT**TCCTGATGCACATCAAGC[e]
12-A	**GCGGCCGC**TAACAGCTTAACGTTGTCC[e]
12-D	**AGATCT**CAGCTTGAAGCGTTGCTT[e]

Restriction enzymes recognition sites are shown in bold.

[a] Priming sequence for the Km resistance cassette underlined.

[b] Priming sequence for the PcL promoter underlined.

[c] Priming sequence for plasmid pWRG717 underlined, used for Km resistance cassette and I-SceI site amplification.

[d] Priming sequence for the *phoP* promoter underlined.

[e]Equivalences of gene names and the internal code used to name primers utilized for pKO3blue experiments: 01 (*adrA*); 02 (*yeaJ*); 03 (*yciR*); 04 (*sen1023*); 05 (*yegE*); 06 (*yfeA*); 07 (*yfiN*); 08 (*yhdA*); 09 (*sen3222*); 10 (*yhjK*); 11 (*sen4316*); 12 (*sen2484*).
