# Supplementary material for: Lack of the PGA exopolysaccharide in Salmonella as an adaptive trait for survival in the host
Source: PLoS Genet. 2017 May 24;13(5):e1006816. doi: 10.1371/journal.pgen.1006816 (PMC5464674; doi:10.1371/journal.pgen.1006816)
Supplement: S1 Dataset — First column indicates the protein name, and the second column is the phylogenetic tree in standard newick format. Labels indicate species names, as retrieved from NCBI, followed by an underscore separator and the gene identifier. (PDF) [file pgen.1006816.s010.pdf]

pgaA

(Escherichia_hermannii_NBRC_105704__gi377539940:0.00625527,(Enterobacter__
gi758509317:0.00000014,Enterobacter__gi918527196:0.00251675)0.812000:0.00674211,
(((((Escherichia_fergusonii__gi803575878:0.00356300,(Escherichia_fergusonii__gi4
46209335:0.00118221,Escherichia_fergusonii__gi446209336:0.00236838)0.000000:0.00
000009)0.460000:0.00118155,(Escherichia_fergusonii__gi446209337:0.00236458,(Esch
erichia_fergusonii__gi803568292:0.00000009,Escherichia_fergusonii__gi803577752:0
.00594488)0.807000:0.00118145)0.000000:0.00000008)0.998000:0.10689496,(Escherich
ia_coli__gi446209618:0.00133147,(Escherichia_coli__gi486366124:0.00393869,((Esch
erichia_coli__gi545168572:0.00662716,(Escherichia__gi446209585:0.00729077,Entero
bacteriaceae__gi550712596:0.00241701)0.879000:0.00309031)0.981000:0.00944571,(Es
cherichia_sp._1_1_43__gi404292140:0.00199477,(Escherichia_coli__gi1048098491:0.0
0000001,(Klebsiella_pneumoniae_IS22__gi571215631:0.00537053,((PgaA:0.00000001,(E
scherichia_coli__gi485810325:0.00483367,(Escherichia_coli__gi446209606:0.0012053
3,Proteobacteria__gi693065241:0.00120524)0.000000:0.00000010)0.000000:0.00000009
)0.000000:0.00000004,(Shigella_boydii__gi1043572726:0.00684104,(Escherichia_sp._
1_1_43__gi404292144:0.00000001,Enterobacteriaceae__gi446209603:0.00000001)0.0000
00:0.00000001)0.000000:0.00000010)0.000000:0.00000002)0.000000:0.00000001)0.0000
00:0.00000009)0.918000:0.00460361)0.381000:0.00295178)0.900000:0.00564889)1.0000
00:0.15919823)1.000000:0.31713223,(Enterobacter_cloacae__gi1015388413:0.00000009
,Enterobacter_cloacae__gi970312386:0.00000001)1.000000:0.50783845)0.270000:0.077
07897,((((Xenorhabdus_nematophila__gi754590956:0.00262438,(Xenorhabdus_nematophi
la__gi498911579:0.00131220,(Xenorhabdus_nematophila__gi740384790:0.00131121,(Xen
orhabdus_nematophila__gi502949677:0.00000001,Xenorhabdus_nematophila_AN6/1__gi73
3163172:0.00000010)0.836000:0.00128129)0.000000:0.00000004)0.000000:0.00000004)1
.000000:0.08599666,(Xenorhabdus_khoisanae__gi847177222:0.09114000,(Xenorhabdus__
gi836618974:0.08793089,(Xenorhabdus_doucetiae__gi661561974:0.00000008,Xenorhabdu
s_doucetiae__gi800943131:0.00000001)1.000000:0.09342868)0.990000:0.04629725)0.36
1000:0.03115641)1.000000:0.39468095,(Serratia_marcescens__gi839841902:0.00038140
,((Serratia_marcescens__gi1039035331:0.00000050,(Serratia_marcescens_WW4__gi4452
13636:0.00237756,Serratia_marcescens_EGD-
HP20__gi542020459:0.00118777)0.771000:0.00237670)0.248000:0.00257756,((Serratia_
marcescens_subsp._marcescens_ATCC_13880__gi669002448:0.00000009,Serratia_marcesc
ens__gi977033193:0.00237551)0.977000:0.00815554,((Serratia_marcescens__gi9773745
55:0.00000001,Serratia_marcescens__gi977354685:0.00118546)0.887000:0.00238230,((
Serratia_marcescens__gi977898467:0.00118736,(Serratia_marcescens__gi838730960:0.
00000007,Serratia_marcescens__gi977771663:0.00118721)0.000000:0.00000007)0.93200
0:0.00237467,(Serratia_marcescens__gi977170984:0.00000004,(Klebsiella_oxytoca__g
i1030351233:0.00356397,((Serratia_marcescens__gi668659703:0.00118440,(Serratia_m
arcescens__gi977342320:0.00237016,(Serratia_marcescens__gi977307298:0.00118463,(
(Serratia_marcescens__gi977361128:0.00118629,(Serratia_marcescens__gi977621173:0
.00000009,(Serratia_marcescens__gi977806098:0.00236783,Serratia_marcescens__gi97
7631739:0.00000008)0.855000:0.00118389)0.000000:0.00000009)0.851000:0.00118462,(
Serratia_marcescens_SM39__gi573011630:0.00118491,(Serratia_marcescens__gi9773281
81:0.00000001,(Serratia_marcescens__gi977080985:0.00595022,(Serratia_marcescens_
_gi928491917:0.00118325,Serratia_marcescens__gi839833627:0.00000001)0.888000:0.0
0238224)0.739000:0.00117746)0.000000:0.00000008)0.000000:0.00000008)0.000000:0.0
0000008)0.000000:0.00000009)0.000000:0.00000008)0.000000:0.00000008,(Serratia_ma
rcescens_BIDMC_80__gi612278636:0.00118533,Serratia_marcescens__gi976948631:0.000
00010)0.793000:0.00118449)1.000000:0.01316636)0.000000:0.00000004)0.000000:0.000
00012)0.760000:0.00118391)0.943000:0.00496231)0.921000:0.00479908)0.874000:0.004
18356)1.000000:0.42966794)0.999000:0.22339093,(((Enterobacter__gi918528554:0.000
00011,Escherichia_hermannii__gi488365872:0.00710920)1.000000:0.26434891,(((Franc
onibacter_helveticus__gi639214329:0.00000009,Franconibacter_helveticus__gi896322
989:0.00000001)0.982000:0.03574360,(Franconibacter_pulveris__gi657886487:0.00763
443,(Cronobacter_sp._DJ34__gi890459514:0.00115854,(Franconibacter_pulveris__gi63
9223186:0.00000001,Franconibacter_pulveris__gi639218661:0.00115801)0.000000:0.00
000001)0.982000:0.00869333)0.987000:0.03899607)1.000000:0.27361786,(((Plesiomona
s_shigelloides__gi743527885:0.00644365,Plesiomonas_sp._ZOR0011__gi835946970:0.01
109338)1.000000:0.30091616,((Citrobacter_sp._S-

77__gi780038527:0.00000001,Citrobacter_sp._MGH106__gi851927720:0.00115984)0.9940
00:0.03295030,((Citrobacter__gi851904789:0.00195581,(Citrobacter_pasteurii__gi74
9609715:0.00354537,Shigella_flexneri_1235-
66__gi391321755:0.00116144)0.965000:0.00751936)1.000000:0.03156600,(((Enterobact
eriaceae__gi489922183:0.00467223,((Citrobacter_sp._BIDMC108__gi851937121:0.00233
421,((Enterobacteriaceae__gi949724366:0.00000001,Citrobacter_sp._MGH105__gi85191
2056:0.00116606)0.000000:0.00000009,(Citrobacter_sp._30_2__gi538025766:0.0011660
9,Citrobacter_freundii__gi696369877:0.00116741)0.000000:0.00000008)0.000000:0.00
000009)0.844000:0.00116639,(Citrobacter_sp._MGH110__gi851970010:0.00116704,(Citr
obacter_freundii__gi927047161:0.00000010,Citrobacter_freundii__gi983531643:0.000
00008)0.936000:0.00233291)0.000000:0.00000009)0.000000:0.00000009)0.831000:0.002
25503,((Citrobacter_freundii__gi560879369:0.00124789,(Citrobacter__gi489124055:0
.00116540,((Citrobacter_freundii_complex__gi696375471:0.00000001,Citrobacter_fre
undii__gi970961643:0.00233138)0.000000:0.00000008,(Citrobacter_sp._MGH103__gi851
922910:0.00232985,Citrobacter_freundii__gi949719544:0.00233051)0.000000:0.000000
14)0.854000:0.00116489)0.000000:0.00000010)0.000000:0.00000010,(((Citrobacter_sp
._AATXR__gi1004675524:0.00233445,(Citrobacter_freundii_ATCC_8090_=_MTCC_1658__gi
668711520:0.00000009,(Citrobacter_freundii__gi985306141:0.00000001,(Citrobacter_
sp._KTE30__gi507080182:0.00116744,(Citrobacter_freundii__gi489937692:0.00000001,
(Citrobacter_sp._MGH109__gi851981014:0.00116668,Citrobacter_freundii_complex__gi
696362293:0.00000001)0.844000:0.00116614)0.000000:0.00000009)0.000000:0.00000010
)0.000000:0.00000010)0.831000:0.00116618)0.000000:0.00000010,(Citrobacter_sp._MG
H104__gi851961160:0.00116619,(Citrobacter_freundii__gi765457548:0.00000009,(Citr
obacter_freundii_GTC_09479__gi455642882:0.00000009,Citrobacter_freundii__gi69636
4168:0.00000013)0.901000:0.00116620)0.000000:0.00000009)0.000000:0.00000010)0.00
0000:0.00000009,((Citrobacter_freundii__gi949802701:0.00000001,(Citrobacter_freu
ndii__gi949788322:0.00000004,Citrobacter_freundii__gi895900140:0.00386596)0.0000
00:0.01034696)0.000000:0.00000013,Citrobacter_freundii__gi949787355:0.00000013)0
.836000:0.00116740)0.842000:0.00116712)0.783000:0.00126379)0.853000:0.00354709,(
((Citrobacter_freundii__gi757800262:0.00116113,(Citrobacter_freundii__gi83523227
5:0.00000001,Citrobacter_freundii__gi1046456174:0.00116460)0.000000:0.00000001)0
.981000:0.00928551,(Citrobacter_braakii__gi896286132:0.00233403,(Citrobacter__gi
507083559:0.00000010,(Citrobacter_braakii__gi835241545:0.00116662,Enterobacter_s
p._GN02600__gi829876999:0.00000014)0.845000:0.00116711)0.000000:0.00000014)0.929
000:0.00492685)0.973000:0.00859893,((Citrobacter_freundii__gi740848154:0.0011598
8,(Enterobacteriaceae__gi735677197:0.00000001,((Enterobacter_asburiae__gi7429351
49:0.00116142,Enterobacter_cloacae_complex__gi782724661:0.00000008)0.880000:0.00
116291,(Enterobacter_asburiae__gi798886125:0.00116195,Enterobacter_cloacae_compl
ex__gi949716452:0.00000001)0.866000:0.00116182)0.000000:0.00000013)0.756000:0.00
116254)0.983000:0.00953973,((Citrobacter_werkmanii__gi536720639:0.00000004,(Citr
obacter_werkmanii__gi754954701:0.00116504,(Salmonella_enterica__gi1002399645:0.0
0116567,Salmonella_enterica__gi1002470781:0.00000011)0.000000:0.00000011)0.00000
0:0.00000004)0.924000:0.00581552,(Citrobacter_youngae__gi696365395:0.00000014,Ci
trobacter_youngae_ATCC_29220__gi291068523:0.00000001)0.999000:0.01657373)0.19400
0:0.00229835)0.966000:0.00936098)0.504000:0.00415504)0.996000:0.02221678)0.71800
0:0.01685708)0.958000:0.03777339)1.000000:0.09625131,((Escherichia_vulneris__gi7
55035127:0.00000009,Escherichia_vulneris_NBRC_102420__gi689830595:0.00000001)1.0
00000:0.15102815,(Enterobacter_sp._Bisph2__gi745786028:0.16006300,(Leclercia_ade
carboxylata__gi743511000:0.02355403,(Enterobacter_sp._Bisph1__gi745757885:0.0587
2149,(Leclercia_adecarboxylata__gi976149804:0.00224509,(Leclercia_adecarboxylata
__gi695646313:0.00000005,Leclercia_adecarboxylata_ATCC_23216_=_NBRC_102595__gi66
8974511:0.00000001)0.774000:0.00128248)0.999000:0.03190561)0.217000:0.01726252)1
.000000:0.13574763)1.000000:0.13522125)0.987000:0.06399600)1.000000:0.20481071)0
.979000:0.07987776)0.454000:0.04547145,((Kosakonia_oryzae__gi1035730536:0.113229
49,(Trabulsiella_odontotermitis__gi906993096:0.00203384,Trabulsiella_odontotermi
tis__gi908733475:0.00589424)1.000000:0.11953525)1.000000:0.26409650,(((Enterobac
ter__gi823311526:0.00273774,(Enterobacter__gi895985413:0.00353213,(((Enterobacte
r__gi779948663:0.00117009,Enterobacter__gi1022693953:0.00000001)0.774000:0.00116
824,(Enterobacter__gi823290369:0.00117015,(Enterobacteriaceae__gi695792712:0.000
00008,Enterobacter__gi695792482:0.00117401)0.934000:0.00352598)0.743000:0.001174

23)0.784000:0.00117070,(Enterobacter__gi823324577:0.00000013,Enterobacter__gi102
2657161:0.00234373)0.000000:0.00000009)0.756000:0.00116345)0.914000:0.00444076)0
.963000:0.01600689,(Enterobacter__gi823286003:0.00240897,(((Enterobacter__gi8959
75091:0.00000013,(Enterobacter__gi823298955:0.00234715,(Enterobacter__gi69579928
5:0.00352185,Enterobacter__gi970305703:0.00000005)0.000000:0.00000005)0.830000:0
.00117220)0.954000:0.00352367,(Enterobacter__gi695795433:0.00000001,Enterobacter
__gi1032322598:0.00117236)0.780000:0.00117319)0.783000:0.00117212,((Enterobacter
__gi836582834:0.00117221,(Enterobacter__gi851950765:0.00824756,(Enterobacter__gi
823302256:0.00000001,((Enterobacter__gi895947557:0.00469927,Enterobacter__gi7576
55726:0.00117173)0.000000:0.00000009,((Enterobacter__gi651618314:0.00234634,Ente
robacter__gi823303341:0.00000008)0.478000:0.00117214,((Enterobacter__gi102269322
2:0.00117209,(Enterobacter__gi779966423:0.00705466,Enterobacter__gi823326189:0.0
0000014)0.799000:0.00117245)0.000000:0.00000009,(Enterobacter__gi823297669:0.001
17265,(Enterobacter__gi1022634789:0.00000008,((Enterobacter__gi779932195:0.00235
028,(Enterobacter__gi822023678:0.00117333,Enterobacter__gi1022689041:0.00000009)
0.768000:0.00117574)0.871000:0.00234926,(Enterobacter__gi779883424:0.00117383,En
terobacter__gi823323249:0.00352660)0.000000:0.00000010)0.000000:0.00000008)0.953
000:0.00352699)0.747000:0.00117303)0.914000:0.00234564)0.000000:0.00000008)0.927
000:0.00117243)0.000000:0.00000001)0.000000:0.00000010)0.857000:0.00117216,(Ente
robacter__gi695801281:0.00352254,(Enterobacter__gi976146720:0.00234522,(Enteroba
cter__gi981223214:0.00117215,(Enterobacter__gi823289370:0.00000013,(Enterobacter
__gi823281145:0.00117188,Enterobacter__gi779891907:0.00000001)0.858000:0.0011717
2)0.848000:0.00117271)0.000000:0.00000008)0.000000:0.00000011)0.000000:0.0000000
9)0.000000:0.00000013)0.978000:0.00827410)0.183000:0.00650155)0.999000:0.1205039
1,(Klebsiella_pneumoniae__gi1000905073:0.00929920,(((Klebsiella_pneumoniae__gi81
7656028:0.00000009,(Klebsiella_pneumoniae__gi1031361802:0.00000001,Klebsiella_pn
eumoniae__gi1031858112:0.00000001)0.918000:0.00234804)0.859000:0.00117366,((Kleb
siella_variicola__gi895850273:0.00000001,(Klebsiella_pneumoniae__gi839615841:0.0
0000001,Klebsiella_pneumoniae__gi852283344:0.00000001)0.858000:0.00117339)0.9100
00:0.00117348,(Klebsiella_variicola__gi895911461:0.00000004,(Klebsiella_pneumoni
ae__gi985816440:0.00117403,((Klebsiella_sp._KTE92__gi500616114:0.00000001,Klebsi
ella_sp._KTE92__gi695768118:0.00000001)0.953000:0.00352415,((Klebsiella_variicol
a__gi906285813:0.00000008,Klebsiella_pneumoniae_subsp._pneumoniae_KP5-
1__gi660552911:0.00353214)0.000000:0.00000008,(Klebsiella_variicola__gi896143476
:0.00117237,(((Klebsiella_pneumoniae__gi695823429:0.00000001,Klebsiella_pneumoni
ae_UCICRE_10__gi555048126:0.00000001)0.964000:0.00469348,(Klebsiella_pneumoniae_
_gi1031835242:0.00000001,Klebsiella_pneumoniae__gi1030846072:0.00000001)0.892000
:0.00234248)0.400000:0.00117251,(Klebsiella_pneumoniae__gi1030397297:0.00000001,
Klebsiella_pneumoniae__gi1031842254:0.00000010)0.000000:0.00000001)0.898000:0.00
234567)0.000000:0.00000008)0.861000:0.00117295)0.000000:0.00000013)0.860000:0.00
117383)0.000000:0.00000004)0.000000:0.00000010)0.824000:0.00117361,((((Klebsiell
a_variicola_CAG:0.00000001,Klebsiella_pneumoniae__gi1031812528:0.00000001)0.7730
00:0.00117280,(Klebsiella_pneumoniae_MGH_80__gi636423752:0.00000001,Klebsiella_p
neumoniae__gi695823054:0.00000001)0.803000:0.00117233)0.797000:0.00117368,(Klebs
iella__gi695817919:0.00000001,Klebsiella_pneumoniae_UCI_18__gi583511027:0.000000
09)0.000000:0.00000001)0.910000:0.00117212,(Klebsiella_pneumoniae_BIDMC_61__gi63
5941410:0.00000001,(Klebsiella_pneumoniae__gi695843011:0.00000001,((((((Klebsiel
la_pneumoniae_subsp._pneumoniae_BJ1-
GA__gi573070445:0.00117459,(Klebsiella_pneumoniae__gi694094956:0.00000009,(Klebs
iella_pneumoniae_NB60__gi583437058:0.00000001,(Klebsiella_pneumoniae__gi10318760
75:0.00000009,(Klebsiella_pneumoniae__gi1030367732:0.00000001,(Klebsiella_pneumo
niae__gi694072308:0.00000001,Klebsiella_pneumoniae_BWH_2__gi583686014:0.00000001
)0.916000:0.00117030)0.000000:0.00000001)0.910000:0.00117078)0.000000:0.00000009
)0.776000:0.00117251)0.881000:0.00234837,(Klebsiella_pneumoniae__gi1030972819:0.
00000001,Klebsiella_pneumoniae__gi1031823935:0.00000001)0.894000:0.00234985)0.88
6000:0.00234641,((Klebsiella_pneumoniae__gi1031741499:0.00000001,Klebsiella_pneu
moniae__gi749506488:0.00000001)0.875000:0.00235106,(Klebsiella_pneumoniae_MGH_39
__gi583717040:0.00117443,(Klebsiella_pneumoniae__gi820953183:0.00117558,(Klebsie
lla_pneumoniae__gi749042112:0.00000001,Klebsiella_pneumoniae__gi699974582:0.0000
0001)0.798000:0.00117265)0.885000:0.00234949)0.753000:0.00117519)0.799000:0.0011

7324)0.000000:0.00000008,((Klebsiella_pneumoniae__gi1043625968:0.00000001,Klebsi
ella_pneumoniae__gi1045804001:0.00000001)0.981000:0.00470810,(Klebsiella_pneumon
iae__gi749588492:0.00234722,(Klebsiella_pneumoniae__gi1044143687:0.00000001,Kleb
siella_pneumoniae__gi523671461:0.00000001)0.789000:0.00117585)0.789000:0.0011737
2)0.000000:0.00000014)1.000000:0.02018136,((Klebsiella_pneumoniae__gi1031745218:
0.00000001,Klebsiella_pneumoniae__gi1032817855:0.00000001)0.852000:0.00117217,(K
lebsiella_variicola__gi751261293:0.00117055,(Klebsiella_pneumoniae__gi695820284:
0.00000001,Klebsiella_pneumoniae_MGH_68__gi636376923:0.00000001)0.799000:0.00117
184)0.792000:0.00117149)0.000000:0.00000009)0.000000:0.00000009,(Klebsiella_vari
icola__gi1016530657:0.00351756,((Klebsiella_pneumoniae__gi501537268:0.00352440,(
Klebsiella_variicola__gi906290086:0.00352073,((Klebsiella_pneumoniae__gi10307585
91:0.00000001,Klebsiella_pneumoniae__gi1031814102:0.00000001)0.937000:0.00352332
,(Klebsiella_pneumoniae__gi835816254:0.00000010,Klebsiella_pneumoniae__gi1031268
450:0.00234292)0.291000:0.00117410)0.722000:0.00117030)0.775000:0.00117124)0.000
000:0.00000008,((Klebsiella_pneumoniae__gi1031862399:0.00000007,(Klebsiella_pneu
moniae__gi1031180678:0.00000001,(Klebsiella__gi695825454:0.00000001,Klebsiella_p
neumoniae_MGH_40__gi555124307:0.00000001)0.910000:0.00117131)0.000000:0.00000009
)0.851000:0.00117148,((Klebsiella_variicola__gi896131203:0.00471343,(Klebsiella_
pneumoniae__gi1031849057:0.00000001,Klebsiella_pneumoniae__gi1031294775:0.000000
01)0.786000:0.00117065)0.763000:0.00117613,((Klebsiella__gi822021700:0.00000008,
(Klebsiella_pneumoniae__gi746030077:0.00000001,(Klebsiella_variicola_At-
22__gi288892284:0.00000001,Klebsiella_variicola__gi752466505:0.00000001)0.851000
:0.00117146)0.958000:0.00352055)0.832000:0.00117173,(Klebsiella_sp._1_1_55__gi49
6082721:0.00000001,(Klebsiella_variicola__gi906304386:0.00000001,(Klebsiella_pne
umoniae__gi1031847705:0.00000001,Klebsiella_pneumoniae__gi1030987628:0.00000001)
0.961000:0.00351980)0.000000:0.00000001)0.000000:0.00000010)0.000000:0.00000008)
0.000000:0.00000008)0.843000:0.00117140)0.000000:0.00000009)0.000000:0.00000008)
0.930000:0.00117174)0.000000:0.00000001)0.000000:0.00000009)0.000000:0.00000009)
0.936000:0.01467246)1.000000:0.27926630)1.000000:0.38614530)0.675000:0.03750796)
0.996000:0.15227584)1.000000:0.42539986)1.000000:0.73930116);
pgaB (Shigella_flexneri_SFJ17B_gi335576371:0.00369732,Shigella_flexneri_K-
218_gi333006267:0.00370471,((Proteobacteria_gi446868305:0.00000007,(Escherichia_
coli_gi446868324:0.00275381,(Escherichia_coli_gi1016524433:0.00171264,PgaB:0.000
00007)0.000000:0.00000007)0.000000:0.00000016)0.976000:0.01088920,(((Escherichia
_sp._TW15838_gi446868285:0.00823896,Escherichia_coli_gi486159179:0.00136960)0.00
0000:0.00000010,(Escherichia_coli_gi446868292:0.00830958,((Enterobacteriaceae_gi
550712595:0.00137134,Escherichia_coli_gi519082975:0.00413070)0.979000:0.01334021
,(((Escherichia_fergusonii_gi803575877:0.00334199,(Escherichia_fergusonii_gi4468
67382:0.00749061,(Escherichia_fergusonii_gi446867381:0.00162230,Escherichia_ferg
usonii_gi446867383:0.00408514)0.739000:0.00188407)0.769000:0.00276291)0.882000:0
.00657427,(Escherichia_fergusonii_gi803568294:0.01239591,Escherichia_fergusonii_
gi803577753:0.00000007)0.825000:0.00659576)0.993000:0.07703104,(Enterobacter_clo
acae_gi970313466:0.31505150,(Pusillimonas_sp._T7-
7_gi503507648:0.46104396,((Erwinia_sp._Leaf53_gi947571648:0.31059220,((Serratia_
ficaria_gi1006546986:0.03413878,((Serratia_marcescens_gi896295582:0.00139572,((S
erratia_marcescens_gi852259667:0.00139206,(Serratia_gi665934509:0.00000001,Serra
tia_marcescens_EGD-
HP20_gi542020458:0.00143245)0.000000:0.00000009)0.000000:0.00000009,(Serratia_ne
matodiphila_gi727160022:0.00139131,Serratia_marcescens_gi505191539:0.00278476)0.
000000:0.00000010)0.781000:0.00139340)0.714000:0.00169596,((Serratia_marcescens_
gi896178927:0.00140173,(Serratia_marcescens_gi727173310:0.00280666,Serratia_marc
escens_gi983123903:0.00140158)0.000000:0.00000009)0.787000:0.00151363,(Serratia_
marcescens_gi654668837:0.00141409,((Serratia_marcescens_gi896190975:0.00139582,E
nterobacteriaceae_gi896287403:0.00000009)0.910000:0.00279498,((Serratia_gi983090
938:0.00278956,(Serratia_marcescens_gi983069727:0.00139479,(Serratia_marcescens_
gi727186249:0.00000001,(Serratia_marcescens_gi983122066:0.00139276,(Serratia_mar
cescens_gi896187073:0.00139430,((Serratia_marcescens_gi751567234:0.00139319,((Se
rratia_marcescens_gi983084684:0.00139176,Serratia_marcescens_gi983088055:0.00139
331)0.797000:0.00139384,(Enterobacteriaceae_gi896235748:0.00139319,Serratia_marc
escens_gi835584788:0.00139613)0.000000:0.00000009)0.000000:0.00000012)0.000000:0

.00000008,(Serratia_marcescens_gi746531731:0.00139403,(Serratia_marcescens_gi727
191943:0.00000009,Serratia_marcescens_gi983100365:0.00278891)0.000000:0.00000014
)0.000000:0.00000008)0.000000:0.00000008)0.929000:0.00278921)0.000000:0.00000010
)0.000000:0.00000009)0.000000:0.00000017)0.932000:0.00279522,((Serratia_sp._YD25
_gi1028171349:0.00000009,Serratia_marcescens_gi518283600:0.00138938)0.941000:0.0
0418538,((Serratia_marcescens_gi742397428:0.00139528,Serratia_marcescens_gi83657
2328:0.00000010)0.980000:0.00700042,(Serratia_marcescens_gi983101248:0.00278823,
Serratia_gi983070374:0.00000010)0.795000:0.00138903)0.769000:0.00140460)0.899000
:0.00279465)0.000000:0.00000017)0.962000:0.00682722)0.407000:0.00159287)0.866000
:0.00381443)0.999000:0.06540219)1.000000:0.21231381,(((Xenorhabdus_poinarii_gi80
0929704:0.06906656,(Xenorhabdus_gi836618898:0.03113206,Xenorhabdus_doucetiae_gi8
00943133:0.03468859)0.986000:0.02472478)0.696000:0.01027648,((Xenorhabdus_nemato
phila_gi754590957:0.00137408,Xenorhabdus_nematophila_gi498911578:0.00000001)1.00
0000:0.07041414,(Xenorhabdus_khoisanae_gi847177185:0.04622215,(Xenorhabdus_szent
irmaii_gi740407651:0.07489167,(Xenorhabdus_sp._NBAII_XenSa04_gi835848803:0.01496
965,Xenorhabdus_cabanillasii_gi740429278:0.01302866)1.000000:0.09165573)0.785000
:0.02178251)0.203000:0.01078812)0.747000:0.01239161)1.000000:0.23543044,((Yersin
ia_entomophaga_gi1035666199:0.01462400,Yersinia_nurmii_gi902506980:0.02346832)1.
000000:0.18942725,((Yersinia_rohdei_gi696237367:0.00143113,Yersinia_rohdei_ATCC_
43380_gi238710235:0.00000001)0.992000:0.03262301,((Yersinia_gi910778590:0.000000
08,(Yersinia_intermedia_ATCC_29909_gi238728143:0.00000001,(Yersinia_intermedia_g
i915758104:0.00137027,(Yersinia_intermedia_gi696330981:0.00000001,Yersinia_inter
media_gi910764947:0.00137133)0.000000:0.00000001)0.000000:0.00000014)0.908000:0.
00274554)0.989000:0.01930798,(Yersinia_gi740848299:0.00891967,Yersinia_kristense
nii_gi910814891:0.00921953)0.962000:0.01269743)0.832000:0.01598224)1.000000:0.15
188944)1.000000:0.11508225)0.736000:0.04848696)0.826000:0.05775593)0.939000:0.05
271370,((((Plesiomonas_shigelloides_gi743527886:0.00143020,Plesiomonas_sp._ZOR00
11_gi835946975:0.00538404)1.000000:0.17383267,(Citrobacter_gi780038519:0.0256551
5,(((Citrobacter_freundii_gi740848152:0.00274146,(Enterobacteriaceae_gi735677196
:0.00000008,(Enterobacter_cloacae_complex_sp._SMART_454_gi970272674:0.00136450,(
Enterobacter_cloacae_gi895868195:0.00136507,(Enterobacter_cloacae_complex_sp._GN
02570_gi836590361:0.00136290,Enterobacter_cloacae_complex_gi742935147:0.00000008
)0.000000:0.00000012)0.000000:0.00000010)0.839000:0.00136549)0.000000:0.00000010
)0.823000:0.00243043,(Citrobacter_youngae_gi493737735:0.01771109,(Citrobacter_we
rkmanii_gi754954698:0.00000001,Salmonella_enterica_gi1002399644:0.00136877)0.000
000:0.00035768)0.235000:0.00274437)0.849000:0.00449922,((Enterobacteriaceae_gi85
1912057:0.00000011,(Citrobacter_sp._30_2_gi496061226:0.00136946,(Citrobacter_fre
undii_complex_gi696369875:0.00000008,(Enterobacteriaceae_gi489922185:0.00000010,
((Citrobacter_sp._MGH104_gi851961155:0.00272895,((Citrobacter_freundii_gi9856289
01:0.00136443,(Citrobacter_freundii_gi489937690:0.00000001,Citrobacter_freundii_
gi970961644:0.00136705)0.000000:0.00000010)0.853000:0.00136411,(Citrobacter_freu
ndii_gi560879371:0.00136414,(Citrobacter_gi489124057:0.00000001,(((Citrobacter_f
reundii_gi936194604:0.00136825,Citrobacter_freundii_gi895900118:0.00136526)0.000
000:0.00000008,(Citrobacter_freundii_complex_gi489943122:0.00000001,Citrobacter_
freundii_gi895919193:0.00136507)0.000000:0.00000001)0.000000:0.00000007,(Citroba
cter_sp._KTE30_gi507080181:0.00411131,(Citrobacter_freundii_gi949787354:0.000000
09,Citrobacter_sp._AATXR_gi1004675522:0.00136516)0.854000:0.00136481)0.000000:0.
00000009)0.943000:0.00136595)0.000000:0.00000016)0.000000:0.00000010)0.000000:0.
00000016)0.920000:0.00411032,(Citrobacter_gi851904787:0.00136526,(Citrobacter_pa
steurii_gi749609717:0.00000008,Shigella_flexneri_1235-
66_gi391321756:0.00137207)0.865000:0.00275685)1.000000:0.01681439)0.949000:0.005
52194)0.823000:0.00136734)0.855000:0.00136625)0.000000:0.00000011)0.947000:0.008
93491,((Citrobacter_amalonaticus_gi808791664:0.00136363,((Citrobacter_sp._KTE151
_gi507083558:0.00548042,Citrobacter_braakii_gi895958856:0.00136917)0.765000:0.00
135866,(Enterobacteriaceae_gi507086658:0.00000009,(Citrobacter_braakii_gi8963035
63:0.00272716,Citrobacter_freundii_gi1045891833:0.00136262)0.000000:0.00000010)0
.000000:0.00000016)0.000000:0.00000010)0.926000:0.00413707,(Citrobacter_freundii
_gi1046456175:0.00000007,Citrobacter_freundii_gi757800261:0.00136407)0.977000:0.
00827125)0.858000:0.00356713)0.020000:0.00411866)0.877000:0.01301403)0.972000:0.
03621353)0.997000:0.07394128,(Escherichia_vulneris_gi755035125:0.15734139,(Enter

obacter_sp._Bisph2_gi745785857:0.15587334,(Leclercia_adecarboxylata_gi976149803:
0.00206040,Leclercia_adecarboxylata_gi695646315:0.00489841)0.999000:0.08910286)1
.000000:0.13104465)0.730000:0.03455500)1.000000:0.10503924,((((Cronobacter_sp._D
J34_gi890454442:0.00000039,Franconibacter_pulveris_gi639223584:0.00961450)1.0000
00:0.15462305,((Enterobacteriaceae_gi639223187:0.01042326,Franconibacter_pulveri
s_gi657886489:0.00384385)0.968000:0.01836673,(Franconibacter_helveticus_gi896322
986:0.00140232,Franconibacter_helveticus_gi639214328:0.00140398)0.996000:0.03303
497)0.957000:0.03412840)1.000000:0.14341967,(Enterobacteriaceae_gi488365874:0.20
340759,(Kosakonia_oryzae_gi1035730534:0.06857678,Trabulsiella_odontotermitis_gi9
08733474:0.07391316)1.000000:0.10409606)0.009000:0.02840193)0.564000:0.03307452,
(((Alcanivorax_sp._43B_GOM-
46m_gi652554633:0.00137954,Alcanivorax_dieselolei_gi941256109:0.00139436)0.00000
0:0.00000013,Alcanivorax_sp._PN-
3_gi552528759:0.00000014)1.000000:0.25970714,((Cedecea_davisae_gi514233591:0.107
50979,(Klebsiella_michiganensis_gi788189869:0.01597180,(Enterobacter_sp._Ag1_gi4
95734747:0.02376652,((Cedecea_neteri_gi740687719:0.02309307,Cedecea_neteri_gi100
1720255:0.01567406)0.960000:0.01044947,(Cedecea_neteri_gi746254323:0.02571637,Ce
decea_neteri_gi746244381:0.01900553)0.865000:0.00773347)0.740000:0.00227948)0.83
8000:0.01284209)1.000000:0.08938819)1.000000:0.09205386,((Kluyvera_ascorbata_gi7
37926522:0.22197632,(Enterobacter_gi779932199:0.00000010,(Enterobacter_gi6957954
31:0.00000001,(Enterobacteriaceae_gi505181576:0.00000001,Enterobacter_gi89596509
4:0.00137038)0.963000:0.00412846)0.756000:0.00137292)1.000000:0.13451367)0.56200
0:0.04034097,(((Klebsiella_quasipneumoniae_subsp._similipneumoniae_gi591277804:0
.00000001,(Klebsiella_quasipneumoniae_gi694114970:0.00000001,(Klebsiella_quasipn
eumoniae_gi895904174:0.00000009,(Klebsiella_pneumoniae_gi759823480:0.00140224,(K
lebsiella_quasipneumoniae_gi896036831:0.00000010,(Klebsiella_pneumoniae_gi103187
5086:0.00421303,(Klebsiella_gi490311702:0.00139861,(Klebsiella_pneumoniae_gi5165
11641:0.00000001,(Klebsiella_pneumoniae_gi852090375:0.00280274,(Klebsiella_gi927
316794:0.00421030,(Klebsiella_pneumoniae_gi1048228412:0.00000001,(Klebsiella_pne
umoniae_gi556467772:0.00000017,((Klebsiella_pneumoniae_gi950167119:0.00139898,Kl
ebsiella_pneumoniae_gi1046631386:0.00140015)0.000000:0.00000017,(Klebsiella_gi89
6073728:0.00000009,(Klebsiella_quasipneumoniae_subsp._similipneumoniae_gi1029974
791:0.00000012,Klebsiella_quasipneumoniae_gi1039431431:0.00280583)0.792000:0.002
80330)0.835000:0.00140079)0.000000:0.00000017)0.913000:0.00139932)0.000000:0.000
00010)0.000000:0.00000008)0.852000:0.00139994)0.000000:0.00000001)0.875000:0.002
80375)0.755000:0.00140542)0.000000:0.00000009)0.951000:0.00280676)0.852000:0.001
40184)0.000000:0.00000001)0.961000:0.00712218,((Klebsiella_quasipneumoniae_gi914
217502:0.00280488,Klebsiella_pneumoniae_gi852307897:0.00000008)0.841000:0.001401
70,(Klebsiella_pneumoniae_gi852159473:0.00000001,((Klebsiella_quasipneumoniae_gi
895915040:0.00139929,(Klebsiella_pneumoniae_gi1031828910:0.00000001,(Klebsiella_
pneumoniae_gi1031831485:0.00139858,(Klebsiella_pneumoniae_gi556292835:0.00280086
,(Klebsiella_quasipneumoniae_subsp._quasipneumoniae_gi591283822:0.00000009,(Kleb
siella_quasipneumoniae_gi694112509:0.00000001,Klebsiella_pneumoniae_gi1048231565
:0.00139753)0.000000:0.00000001)0.858000:0.00139826)0.000000:0.00000008)0.000000
:0.00000008)0.000000:0.00000013)0.715000:0.00139917,(Klebsiella_pneumoniae_gi104
8241188:0.00563637,(Klebsiella_quasipneumoniae_gi764954934:0.00000001,Klebsiella
_quasipneumoniae_subsp._quasipneumoniae_gi635562719:0.00000001)0.753000:0.001399
45)0.000000:0.00139866)0.000000:0.00000001)0.000000:0.00000009)0.905000:0.004207
15)0.892000:0.00983134,((((Klebsiella_pneumoniae_gi1031835243:0.00000001,(Klebsi
ella_variicola_gi906290087:0.00140203,Klebsiella_pneumoniae_subsp._pneumoniae_KP
5-
1_gi660552910:0.00140320)0.000000:0.00000017)0.858000:0.00140439,(Klebsiella_var
iicola_gi896109338:0.00000001,(Klebsiella_pneumoniae_gi1031849061:0.00280817,Kle
bsiella_pneumoniae_gi1031814104:0.00000001)0.840000:0.00140343)0.000000:0.000000
09)0.890000:0.00281571,((Klebsiella_pneumoniae_gi556491715:0.00000010,Klebsiella
_sp._KTE92_gi507089415:0.00279677)0.000000:0.00000010,(Klebsiella_gi762040058:0.
00000004,(Klebsiella_variicola_gi748578592:0.00000001,(Klebsiella_variicola_gi90
6304387:0.00000001,((Klebsiella_gi556471744:0.00000017,(Klebsiella_pneumoniae_gi
695823108:0.00000001,Klebsiella_variicola_gi906293358:0.00000001)0.871000:0.0013
9888)0.842000:0.00139915,(((Klebsiella_pneumoniae_gi1032817878:0.00139949,Klebsi

ella_pneumoniae_gi817656037:0.00139860)0.000000:0.00000009,((Klebsiella_variicol
a_gi906285814:0.00000001,(Klebsiella_gi501537269:0.00000010,(Klebsiella_pneumoni
ae_gi985816448:0.00139956,(Klebsiella_pneumoniae_gi942472443:0.00000053,(Klebsie
lla_variicola_gi896131224:0.00280014,Klebsiella_pneumoniae_gi852043638:0.0027900
7)0.686000:0.00280301)0.719000:0.00139853)0.000000:0.00000008)0.000000:0.0000001
0)0.000000:0.00000010,(Klebsiella_pneumoniae_gi556334801:0.00139670,(Klebsiella_
pneumoniae_gi1000905438:0.00422153,Klebsiella_variicola_gi1031761208:0.00280265)
0.904000:0.00280698)0.812000:0.00140128)0.000000:0.00000009)0.922000:0.00139897,
(Klebsiella_sp._1_1_55_gi496082722:0.00139924,(Klebsiella_pneumoniae_gi983460431
:0.00280386,Klebsiella_gi502734150:0.00000001)0.000000:0.00000012)0.000000:0.000
00017)0.000000:0.00000009)0.000000:0.00000013)0.909000:0.00139918)0.000000:0.000
00008)0.750000:0.00140033)0.765000:0.00280599)0.857000:0.00309482,(Klebsiella_pn
eumoniae_gi1048235632:0.00420535,(Klebsiella_pneumoniae_gi749511745:0.00000012,(
Klebsiella_pneumoniae_gi749527557:0.00000001,(Klebsiella_pneumoniae_gi1031874296
:0.00139815,(Klebsiella_gi490282427:0.00139823,((Klebsiella_pneumoniae_gi6940949
42:0.00139886,((Klebsiella_pneumoniae_gi490281873:0.00000001,(Klebsiella_pneumon
iae_subsp._pneumoniae_Kp13_gi569555056:0.00000010,((Klebsiella_pneumoniae_gi1031
861540:0.00139954,Klebsiella_pneumoniae_gi694066595:0.00000001)0.846000:0.001399
92,(Enterobacteriaceae_gi496666894:0.00140014,(Klebsiella_pneumoniae_gi754539783
:0.00000001,Klebsiella_pneumoniae_subsp._pneumoniae_MGH_78578_gi150957838:0.0000
0001)0.845000:0.00140075)0.000000:0.00000009)0.000000:0.00000007)0.000000:0.0000
0009)0.918000:0.00140230,(Klebsiella_pneumoniae_gi490297093:0.00000001,((Klebsie
lla_pneumoniae_gi1031873574:0.00139804,(Klebsiella_pneumoniae_gi915767369:0.0013
9829,(Klebsiella_pneumoniae_gi852318119:0.00139835,(Klebsiella_pneumoniae_gi5140
77712:0.00139879,Klebsiella_pneumoniae_gi895866912:0.00139828)0.000000:0.0000000
1)0.000000:0.00000001)0.000000:0.00000008)0.000000:0.00000008,((Enterobacteriace
ae_gi556217712:0.00000001,(((Klebsiella_pneumoniae_gi895919361:0.00139777,(Klebs
iella_pneumoniae_gi1031824013:0.00000001,Klebsiella_pneumoniae_gi749532599:0.001
39846)0.000000:0.00000001)0.865000:0.00139836,(Klebsiella_pneumoniae_gi103185919
3:0.00421377,(Klebsiella_pneumoniae_gi1048233422:0.00280026,(Klebsiella_pneumoni
ae_gi1031822719:0.00280188,(Klebsiella_pneumoniae_gi490320566:0.00000001,Klebsie
lla_pneumoniae_subsp._pneumoniae_SA1_gi573081478:0.00000018)0.000000:0.00000001)
0.000000:0.00000010)0.000000:0.00000017)0.830000:0.00139925)0.000000:0.00000017,
(Klebsiella_pneumoniae_gi1031835836:0.00279952,Klebsiella_pneumoniae_909957_gi55
3375623:0.00000001)0.000000:0.00000009)0.000000:0.00000009)0.000000:0.00000009,(
Klebsiella_gi556231024:0.00140095,((Klebsiella_pneumoniae_gi749596823:0.00244795
,Klebsiella_pneumoniae_gi749537601:0.00076337)0.737000:0.00099161,(Klebsiella_pn
eumoniae_gi740647946:0.00139691,Klebsiella_pneumoniae_gi749649069:0.00139748)0.0
00000:0.00000001)0.000000:0.00000009)0.000000:0.00000010)0.911000:0.00139888)0.0
00000:0.00000008)0.000000:0.00000009)0.000000:0.00000015)0.000000:0.00000015,(((
Klebsiella_pneumoniae_gi852041580:0.00000001,(Klebsiella_pneumoniae_gi490290975:
0.00139909,((Klebsiella_pneumoniae_gi985347131:0.00000001,Klebsiella_pneumoniae_
gi1031854125:0.00139825)0.000000:0.00000001,(Klebsiella_pneumoniae_gi694085377:0
.00139814,Klebsiella_pneumoniae_gi694083622:0.00000001)0.000000:0.00000009)0.934
000:0.00280802)0.000000:0.00000009)0.848000:0.00139990,(Klebsiella_pneumoniae_gi
1031830004:0.00140125,(Klebsiella_pneumoniae_gi763385626:0.00000001,Klebsiella_p
neumoniae_gi641640791:0.00000001)0.827000:0.00139764)0.000000:0.00000009)0.85800
0:0.00140222,(Klebsiella_pneumoniae_gi1045804007:0.01283429,((Klebsiella_pneumon
iae_gi981999140:0.00140513,(Klebsiella_pneumoniae_gi749642514:0.00140780,(Klebsi
ella_pneumoniae_gi895890995:0.00140444,Klebsiella_gi852127244:0.00000001)0.00000
0:0.00000010)0.000000:0.00000009)0.000000:0.00000008,(Klebsiella_pneumoniae_gi85
2268364:0.00421644,Klebsiella_pneumoniae_gi918411936:0.00721612)0.000000:0.00000
011)0.746000:0.00141345)0.755000:0.00139159)0.000000:0.00000017)0.854000:0.00139
880)0.000000:0.00000009)0.000000:0.00000001)0.723000:0.00139830)0.942000:0.00703
424)0.998000:0.01841521)0.787000:0.00588328)1.000000:0.15904732)0.961000:0.04801
376)1.000000:0.16584767)0.562000:0.02999293)0.941000:0.04814937)0.999000:0.12013
575)0.545000:0.09837865)1.000000:0.27100636)0.999000:0.18410456)0.999000:0.12530
415)0.668000:0.00192699)0.867000:0.00271616)0.000000:0.00000009,(Escherichia_gi4
46868279:0.00136781,Escherichia_gi446868280:0.00000001)0.936000:0.00411459)0.876
000:0.00432803)0.930000:0.00555214);

pgaC

(Enterobacter_gi760377991:0.00000001,Escherichia_hermannii_gi488367752:0.0
0993670,(((Enterobacter_cloacae_gi970312340:0.00000064,Enterobacter_cloacae_gi97
0313467:0.00319882)1.000000:0.50024286,((Escherichia_coli_B185_gi291434358:0.000
00020,(Escherichia_coli_gi485657747:0.00700264,(Escherichia_coli_B088_gi29132413
6:0.00333897,(Klebsiella_pneumoniae_IS22_gi571215628:0.00349690,(Escherichia_col
i_O7:0.00651087,(((Shigella_flexneri_gi585378719:0.00000001,Shigella_flexneri_5_
str._8401_gi110614584:0.00000012)0.902000:0.00646999,(Escherichia_coli_gi4465331
05:0.00000011,PgaC:0.00000015)0.000000:0.00000014)0.000000:0.00000011,(Proteobac
teria_gi446907221:0.00000001,(Escherichia_fergusonii_gi968934317:0.00349673,(Esc
herichia_gi485663290:0.00000001,Escherichia_coli_CFT073_gi26107446:0.00000015)0.
000000:0.00000001)0.834000:0.00323037)0.000000:0.00000012)0.000000:0.00000013)0.
000000:0.00000014)0.000000:0.00000015)0.000000:0.00000013)0.795000:0.00323270)0.
985000:0.10043969,(Escherichia_fergusonii_gi585374915:0.00000001,(Escherichia_fe
rgusonii_ATCC_35469_gi218356787:0.00000025,Escherichia_fergusonii_gi803577758:0.
00354593)0.000000:0.00000001)0.991000:0.10236614)1.000000:0.23980176)0.725000:0.
05708154,((((Alcanivorax_dieselolei_gi941256106:0.00102257,Alcanivorax_gi5525287
58:0.00569195)1.000000:0.23643776,(((Franconibacter_pulveris_gi657886491:0.00000
029,(Franconibacter_pulveris_gi639220367:0.00336550,Enterobacteriaceae_gi6392231
88:0.00000001)0.788000:0.00337267)0.842000:0.01175868,(Franconibacter_helveticus
_gi896322983:0.00000001,Franconibacter_helveticus_gi639214327:0.00332325)0.92800
0:0.02275543)1.000000:0.11749066,((((Enterobacter_gi822023680:0.00000001,(Entero
bacter_gi895947555:0.00332834,(Enterobacter_gi1022689040:0.00000001,(Enterobacte
r_gi823302253:0.00332385,(Enterobacteriaceae_gi505181577:0.00000001,((Enterobact
er_gi823326187:0.00333122,((Enterobacter_gi779948668:0.00334087,Enterobacter_gi8
23311525:0.00000020)0.851000:0.00334213,(Enterobacter_gi823324578:0.00000001,Ent
erobacter_gi823290375:0.00333640)0.000000:0.00000001)0.853000:0.00333422)0.00000
0:0.00000015,(Enterobacter_gi823303344:0.00333086,(Enterobacter_gi823321951:0.00
333304,Enterobacter_gi518923967:0.00000035)0.851000:0.00333610)0.000000:0.000000
30)0.000000:0.00000022)0.841000:0.00333071)0.000000:0.00000020)0.861000:0.003329
85)0.000000:0.00000022)0.961000:0.05026662,((Klebsiella_variicola_gi896131205:0.
00000079,((Klebsiella_variicola_gi895850272:0.00337597,Klebsiella_pneumoniae_sub
sp._pneumoniae_gi938752759:0.00338098)0.000000:0.00000021,((Klebsiella_pneumonia
e_gi1048235613:0.00338026,(Klebsiella_pneumoniae_gi695827801:0.00338192,(Klebsie
lla_pneumoniae_gi1031862400:0.00338232,(Klebsiella_pneumoniae_gi835816250:0.0033
8297,(Klebsiella_pneumoniae_gi695823053:0.00000001,Klebsiella_variicola_gi103176
1209:0.00338389)0.000000:0.00000001)0.845000:0.00338248)0.000000:0.00000025)0.00
0000:0.00000020)0.000000:0.00000025,((Klebsiella_variicola_gi757718623:0.0033832
6,((Klebsiella_pneumoniae_gi1031824745:0.00340896,Klebsiella_pneumoniae_gi523681
118:0.00260801)0.000000:0.00077762,(Klebsiella_pneumoniae_gi694098570:0.00338611
,Klebsiella_pneumoniae_gi852015874:0.00338523)0.000000:0.00000030)0.958000:0.010
19554)0.000000:0.00000018,(Klebsiella_gi496082723:0.00000001,Klebsiella_pneumoni
ae_gi556491714:0.00338073)0.000000:0.00000001)0.000000:0.00000018)0.000000:0.000
00024)0.757000:0.00338793)0.998000:0.08581129,(Kluyvera_ascorbata_gi737926524:0.
13213893,(Citrobacter_youngae_gi493735845:0.13448059,(((Enterobacter_cloacae_com
plex_gi527036932:0.00000013,((Enterobacter_cloacae_EcWSU1_gi365750981:0.00000001
,Enterobacter_cloacae_complex_gi752455035:0.00000001)0.829000:0.00320296,(Entero
bacter_sp._BIDMC_30_gi695757088:0.00321830,(Enterobacter_ludwigii_gi976147833:0.
00319052,Enterobacter_cloacae_complex_gi695752712:0.00000015)0.000000:0.00000013
)0.000000:0.00000014)0.420000:0.00318508)0.984000:0.03519781,((Enterobacter_cloa
cae_gi1006476589:0.00000019,Enterobacter_cloacae_gi740635068:0.00322277)0.834000
:0.00322198,(Enterobacter_gi659669286:0.00000015,((Enterobacter_cloacae_gi742824
627:0.00323018,(Enterobacter_cloacae_gi1016090448:0.00324552,(Enterobacter_cloac
ae_gi772699812:0.00000014,(Enterobacter_cloacae_gi748770067:0.00324168,Proteobac
teria_gi502863722:0.00324051)0.000000:0.00000014)0.000000:0.00000012)0.847000:0.
00324125)0.000000:0.00000028,(Enterobacter_gi970267913:0.00322480,(Enterobacter_
cloacae_gi970309050:0.00322501,(Enterobacter_cloacae_gi970312094:0.00000016,(Ent
erobacter_cloacae_gi504646149:0.00646046,(Enterobacter_sp._SENG-
6_gi1015876505:0.00322457,Enterobacter_cloacae_gi970314221:0.00000012)0.000000:0
.00000019)0.000000:0.00000024)0.851000:0.00322981)0.000000:0.00000021)0.000000:0

.00000013)0.000000:0.00000019)0.000000:0.00000029)0.997000:0.04441213)0.738000:0
.00637280,(((Enterobacter_cloacae_complex_gi504698119:0.00000078,(Enterobacter_g
i556479720:0.00000014,Enterobacter_kobei_gi970327080:0.00323344)0.724000:0.00321
954)0.998000:0.04090088,((Leclercia_gi850372158:0.00653745,(Enterobacter_cloacae
_gi851936165:0.00000020,Enterobacter_cloacae_complex_sp._CIDEIMsCOL1_gi798945946
:0.00326341)0.000000:0.00000020)0.835000:0.00876463,(Enterobacter_cloacae_comple
x_gi779806967:0.02484383,(Enterobacter_sp._EGD-
HP1_gi695704726:0.00324692,Enterobacter_sp._MGH_24_gi556484246:0.00000023)0.9630
00:0.01862176)0.092000:0.00665081)0.893000:0.01425950)0.480000:0.00952827,((Ente
robacter_cloacae_gi980964605:0.00000015,(Enterobacter_sp._BIDMC92_gi847056788:0.
00325102,(Enterobacter_cloacae_complex_sp._35699_gi772676101:0.00326694,Enteroba
cter_cloacae_gi647322737:0.00000019)0.869000:0.00325943)0.000000:0.00000018)0.98
9000:0.03312605,(Enterobacter_cloacae_complex_gi1022700501:0.04383718,(Enterobac
ter_cloacae_complex_sp._GN02283_gi829920271:0.00647588,((Enterobacter_cloacae_co
mplex_gi979846011:0.00323383,(Enterobacter_gi654546693:0.00000006,(Enterobacter_
sp._BIDMC_29_gi754491268:0.00652106,Enterobacter_cloacae_complex_gi829938216:0.0
0323400)0.000000:0.00000006)0.000000:0.00000013)0.000000:0.00000014,(Enterobacte
r_cloacae_gi896108527:0.00647003,Enterobacter_cloacae_gi895899736:0.00000021)0.8
57000:0.00323745)0.000000:0.00000022)0.935000:0.01829593)0.770000:0.00918642)0.1
47000:0.01052526)0.934000:0.02350551)0.070000:0.01711309)0.629000:0.03347496)0.7
01000:0.02950528)0.888000:0.03320711)1.000000:0.26048851,(Trabulsiella_odontoter
mitis_gi908733473:0.05143097,Kosakonia_oryzae_gi1035730532:0.06134381)0.998000:0
.12124381)0.175000:0.02198020,Enterobacteriaceae_gi488365877:0.12731272)0.146000
:0.01988985)0.621000:0.01691813)0.800000:0.02877479,((Escherichia_vulneris_gi755
035123:0.11001386,(Enterobacter_sp._Bisph2_gi745785856:0.05174932,(Leclercia_ade
carboxylata_gi743511002:0.02573904,Leclercia_adecarboxylata_gi695646318:0.038265
41)0.736000:0.03979978)0.948000:0.05472848)0.907000:0.03582261,((Plesiomonas_sp.
_ZOR0011_gi835946980:0.00000001,Plesiomonas_shigelloides_gi743527887:0.00325182)
1.000000:0.15624738,((Enterobacteriaceae_gi507083557:0.00000017,(Citrobacter_bra
akii_gi896286136:0.00327052,Citrobacter_sp._KTE32_gi507086657:0.00328124)0.00000
0:0.00000017)0.753000:0.00350677,(((Citrobacter_werkmanii_gi536720649:0.00335470
,(Salmonella_enterica_gi1002399643:0.00000001,Citrobacter_werkmanii_gi754954697:
0.00326384)0.000000:0.00000001)0.779000:0.00329204,(Citrobacter_youngae_gi493737
736:0.01336042,(Citrobacter_freundii_complex_gi696362295:0.00000017,((Enterobact
eriaceae_gi489124059:0.00000006,(Citrobacter_freundii_gi985628902:0.00328101,Cit
robacter_freundii_gi696369873:0.00328016)0.000000:0.00000006)0.820000:0.00327903
,(Citrobacter_freundii_gi757800260:0.00000650,(Citrobacter_gi780038514:0.0343909
2,(Citrobacter_gi749609719:0.00000017,Shigella_flexneri_1235-
66_gi391321757:0.00000018)0.885000:0.01037850)0.919000:0.01668466)0.860000:0.009
83292)0.000000:0.00000021)0.882000:0.00661319)0.750000:0.00329260)0.897000:0.012
30178,((Citrobacter_freundii_gi740848149:0.00328724,(Enterobacteriaceae_gi735677
194:0.00000001,Enterobacter_cloacae_complex_gi742935145:0.00326981)0.000000:0.00
000001)0.968000:0.02145211,(Citrobacter_freundii_gi835232281:0.01992374,Citrobac
ter_sp._MGH110_gi851970008:0.01000248)0.930000:0.01863368)0.781000:0.00685207)0.
761000:0.00436508)0.907000:0.02486813)0.992000:0.08767089)0.892000:0.03819952)0.
947000:0.08459801,((Erwinia_sp._Leaf53_gi947571650:0.20602692,(Brenneria_goodwin
ii_gi873957859:0.14931427,(Pectobacterium_wasabiae_gi492821367:0.02532273,Pectob
acterium_gi504515082:0.02565191)1.000000:0.32205397)0.822000:0.05412393)0.960000
:0.08528304,((Serratia_ficaria_gi1006546987:0.01751146,((Serratia_gi515493569:0.
00000021,(Serratia_gi505191538:0.00000017,(Serratia_marcescens_gi983382860:0.003
33802,Serratia_marcescens_gi545150261:0.00333398)0.000000:0.00000020)0.867000:0.
00333555)0.673000:0.00271973,(((Serratia_gi742397427:0.00000023,((Serratia_marce
scens_gi518283599:0.00333758,Serratia_sp._YD25_gi1028171348:0.00000021)0.835000:
0.00334061,(Enterobacteriaceae_gi896287400:0.00000035,Serratia_marcescens_gi8961
90976:0.00337057)0.896000:0.00674283)0.000000:0.00000035)0.846000:0.00333710,(Se
rratia_marcescens_gi727186248:0.00334484,Serratia_gi727191945:0.00000012)0.00000
0:0.00000031)0.855000:0.00334142,(Enterobacteriaceae_gi751567232:0.00000001,Serr
atia_marcescens_gi983082276:0.00334151)0.000000:0.00000015)0.751000:0.00399115)0
.899000:0.03123683)1.000000:0.29433974,((Acidovorax_gi947497182:0.35660238,((Bor
detella_bronchiseptica_gi704414125:0.21892189,((Achromobacter_gi1027705330:0.000

00037,(Achromobacter_xylosoxidans_gi721481308:0.00325729,(Achromobacter_xylosoxi
dans_gi503158312:0.00924622,(Achromobacter_sp._gi935582534:0.00318482,Bordetella
_bronchiseptica_gi980956470:0.00319719)0.766000:0.00625407)0.887000:0.01031674)0
.669000:0.00313738)0.937000:0.04668162,(Achromobacter_sp._Root565_gi947909515:0.
01893286,Achromobacter_xylosoxidans_gi1028212780:0.02508224)0.965000:0.05441930)
0.998000:0.16002485)0.993000:0.13731658,(Pseudomonas_sp._TTU2014-
080ASC_gi953984099:0.26027493,(((Pseudomonas_sp._DSM_29164_gi951314327:0.0066249
2,(Pseudomonas_fluorescens_gi734904557:0.00336937,Pseudomonas_gi489279831:0.0033
1379)0.767000:0.00335194)0.723000:0.01032469,(((Pseudomonas_sp._31_R_17_gi104855
8007:0.00000001,(Pseudomonas_orientalis_gi951342804:0.01004098,Pseudomonas_sp._2
8_E_9_gi1048572268:0.00000022)0.824000:0.00332951)0.747000:0.00333337,(Pseudomon
as_sp._34_E_7_gi1048626775:0.00000018,(Pseudomonas_sp._37_R_15_gi1048593738:0.00
664052,Pseudomonas_sp._24_E_13_gi1048573964:0.00000021)0.244000:0.00332538)0.781
000:0.00332635)0.992000:0.03575965,(Pseudomonas_libanensis_gi949578739:0.0000001
4,((Pseudomonas_libanensis_gi980979568:0.00325866,(Pseudomonas_sp._Root569_gi948
189360:0.00000001,Pseudomonas_fluorescens_gi803456122:0.00324952)0.000000:0.0000
0001)0.909000:0.00652411,(Pseudomonas_synxantha_gi949593999:0.00990401,Pseudomon
as_fluorescens_gi758892498:0.00000023)0.000000:0.00135496)0.000000:0.00200241)0.
815000:0.00699861)0.522000:0.01363082)0.994000:0.10557579,(Pseudomonas_gi4893107
67:0.00662184,Pseudomonas_sp._PAMC_25886_gi497854624:0.00000060)0.946000:0.05396
375)1.000000:0.28493314)0.951000:0.08390578)0.936000:0.10229843)0.992000:0.17065
618,((Yersinia_entomophaga_gi1035666201:0.01596137,Yersinia_nurmii_gi902506981:0
.01420253)0.995000:0.09098827,((Yersinia_rohdei_ATCC_43380_gi238710236:0.0000000
1,(Yersinia_rohdei_gi811544544:0.00000001,Yersinia_rohdei_gi696237368:0.00000011
)0.000000:0.00000001)0.989000:0.05866707,(Yersinia_frederiksenii_gi802367628:0.0
0000001,(Yersinia_frederiksenii_gi910775310:0.00000001,(Yersinia_gi518039520:0.0
0000013,(Yersinia_massiliensis_gi893336677:0.00000001,((((Yersinia_mollaretii_gi
812679750:0.00000021,(Yersinia_mollaretii_gi893023241:0.00000001,((Yersinia_moll
aretii_gi902774848:0.00000001,Yersinia_mollaretii_gi893011125:0.00000001)0.91700
0:0.00324343,Yersinia_mollaretii_gi902809189:0.00000013)0.000000:0.00000014)0.92
8000:0.00325021)0.000000:0.00000012,(Yersinia_gi902536936:0.00000001,(Yersinia_m
ollaretii_gi899868412:0.00000001,(Yersinia_mollaretii_gi696235011:0.00000001,Yer
sinia_mollaretii_ATCC_43969_gi238718315:0.00000018)0.000000:0.00000001)0.912000:
0.00327030)0.000000:0.00000001)0.882000:0.00667022,((Yersinia_gi696321439:0.0000
0001,(Yersinia_bercovieri_ATCC_43970_gi238715818:0.00000033,Yersinia_enterocolit
ica_gi802292125:0.00000012)0.000000:0.00000013)0.962000:0.01364385,(Yersinia_gi8
73914046:0.00000001,Yersinia_aleksiciae_gi802305694:0.00000001)0.934000:0.010071
09)0.766000:0.00328630)0.892000:0.00982973,((((Yersinia_pestis_gi514990956:0.005
13923,((Yersinia_pestis_gi919117389:0.00000001,Yersinia_pestis_subsp._microtus_b
v._Ulegeica_gi767764315:0.00000020)0.000000:0.00000013,(Yersinia_pestis_gi510938
856:0.00000001,((Yersinia_pseudotuberculosis_gi740614391:0.00324582,(Yersinia_pe
stis_KIM10+_gi21959212:0.00000001,(Yersinia_pestis_gi4106593:0.00000001,(Yersini
a_pseudotuberculosis_complex_gi488140834:0.00000001,Yersinia_pestis_PY-
02_gi391428321:0.00000024)0.000000:0.00000001)0.912000:0.00327365)0.000000:0.000
00011)0.000000:0.00000012,(Yersinia_gi488153248:0.00000015,((Yersinia_pseudotube
rculosis_gi910803509:0.00324507,Yersinia_pseudotuberculosis_gi742321039:0.003248
29)0.000000:0.00000011,(Yersinia_wautersii_gi806892351:0.00000001,Yersinia_pseud
otuberculosis_complex_gi727729111:0.00000001)0.937000:0.00650238)0.000000:0.0000
0016)0.000000:0.00000021)0.000000:0.00000001)0.000000:0.00000015)0.753000:0.0027
9921)0.898000:0.01217982,(Yersinia_pseudotuberculosis_complex_gi644962940:0.0000
0001,Yersinia_similis_gi802320617:0.00000001)0.828000:0.00623711)0.994000:0.0270
8104,(Yersinia_frederiksenii_gi696334817:0.00000001,(((Yersinia_frederiksenii_gi
893094278:0.00000001,(Yersinia_frederiksenii_gi910795410:0.00000014,(Yersinia_fr
ederiksenii_gi910921799:0.00000001,Yersinia_frederiksenii_gi893121991:0.00000001
)0.925000:0.00323949)0.000000:0.00000015)0.947000:0.00984482,(Yersinia_enterocol
itica_LC20_gi595641326:0.00657936,((Yersinia_pekkanenii_gi893588997:0.00000001,Y
ersinia_pekkanenii_gi902545462:0.00000001)1.000000:0.02996377,(((Yersinia_kriste
nsenii_gi892532037:0.00000001,Yersinia_kristensenii_gi910814892:0.00000001)0.967
000:0.01310918,((Yersinia_kristensenii_gi892957535:0.00000001,Yersinia_kristense
nii_gi898207785:0.00000001)0.945000:0.00969680,(Yersinia_gi740848296:0.00000001,

Yersinia_kristensenii_gi755437730:0.00000014)0.000000:0.00000001)0.401000:0.0032
9618)0.997000:0.02300939,(Yersinia_frederiksenii_gi802314319:0.00000014,(Yersini
a_frederiksenii_gi912948511:0.00326033,Yersinia_frederiksenii_gi910807564:0.0000
0001)0.000000:0.00000017)0.878000:0.00655438)0.762000:0.00324380)0.760000:0.0032
6064)0.753000:0.00332150)0.765000:0.00323017,(Yersinia_frederiksenii_gi802373979
:0.00000005,Yersinia_frederiksenii_ATCC_33641_gi238724778:0.00000035)0.000000:0.
00000005)0.000000:0.00000011)0.928000:0.00983466)0.742000:0.00339315,(((Yersinia
_aldovae_gi755640857:0.00000001,(Yersinia_aldovae_gi892407013:0.00000001,(Yersin
ia_aldovae_gi892961892:0.00000011,(Yersinia_aldovae_gi902533984:0.00000001,(Yers
inia_aldovae_ATCC_35236_gi238704723:0.00339410,(Yersinia_aldovae_gi757587926:0.0
0000001,Yersinia_aldovae_gi892443252:0.00000010)0.000000:0.00000001)0.860000:0.0
0323622)0.000000:0.00000001)0.933000:0.00324070)0.000000:0.00000001)0.999000:0.0
3370526,(Yersinia_gi696330983:0.00000001,(Yersinia_intermedia_ATCC_29909_gi23872
8144:0.00000030,Yersinia_intermedia_gi805211034:0.00000012)0.000000:0.00000012)0
.934000:0.01336380)0.733000:0.00314351,(Yersinia_enterocolitica_gi1032683930:0.0
0000015,((Yersinia_enterocolitica_gi896647615:0.00000001,Yersinia_enterocolitica
_gi893285834:0.00000001)0.915000:0.00328369,((Yersinia_enterocolitica_gi10003671
79:0.00000001,Yersinia_enterocolitica_gi901635303:0.00000001)0.924000:0.00328380
,((Yersinia_enterocolitica_gi802268241:0.00000001,Yersinia_enterocolitica_gi6851
71420:0.00360081)0.000000:0.00000015,(Yersinia_enterocolitica_gi800187810:0.0000
0001,(Yersinia_gi491306392:0.00000001,Yersinia_enterocolitica_IP_10393_gi4317889
46:0.00000001)0.000000:0.00000013)0.000000:0.00000001)0.000000:0.00000011)0.0000
00:0.00000033)0.000000:0.00000018)0.888000:0.00665630)0.762000:0.00332346)0.9070
00:0.00985703)0.947000:0.01316787)0.000000:0.00000015)0.915000:0.00326552)0.0000
00:0.00000001)0.953000:0.03418984)0.973000:0.05832782)1.000000:0.15761134)0.4970
00:0.02266269)0.571000:0.04368599)0.874000:0.07665037)1.000000:0.37535856)1.0000
00:0.49900516);
pgaD
(Enterobacter_gi760377993:0.02925177,Escherichia_hermannii_gi488367753:0.0
1293267,((Enterobacter_cloacae_gi970312339:1.05548116,((Escherichia_fergusonii_g
i446983830:0.01522505,Escherichia_fergusonii_gi803575876:0.02169645)0.875000:0.1
7854398,(Enterobacteriaceae_gi446983835:0.01855773,(Escherichia_sp._1_1_43_gi404
292177:0.00000001,((Proteobacteria_gi446983839:0.00000044,((Shigella_flexneri_gi
745763616:0.00000001,PgaD:0.00000027)0.000000:0.00000034,(Shigella_flexneri_gi74
5764778:0.01306199,(Shigella_dysenteriae_gi585368865:0.03948523,Bacteria_gi44628
7246:0.06372613)0.950000:0.07788810)0.000000:0.00000018)0.000000:0.00000018)0.00
0000:0.00000029,(Escherichia_coli_gi446983842:0.00685134,Escherichia_gi446983843
:0.00000039)0.746000:0.00685472)0.000000:0.00000001)0.641000:0.00997133)0.971000
:0.32110003)0.972000:0.69307697)0.808000:0.33644449,((Acinetobacter_gi491181253:
0.00000248,(Acinetobacter_calcoaceticus_gi515957497:0.00684755,(Acinetobacter_ca
lcoaceticus_gi507066924:0.02129885,(Acinetobacter_calcoaceticus_gi779996783:0.01
434635,((Acinetobacter_sp._WC-
141_gi496869143:0.00723517,Acinetobacter_oleivorans_gi734502263:0.01417782)0.961
000:0.02909603,(((Acinetobacter_pittii_gi507069792:0.01385350,(Acinetobacter_bau
mannii_gi691140938:0.00708631,(Acinetobacter_pittii_gi949736151:0.00000001,Acine
tobacter_calcoaceticus/baumannii_complex_gi491210177:0.00000027)0.000000:0.00000
001)0.949000:0.02959565)0.862000:0.02216637,Acinetobacter_calcoaceticus_gi770625
180:0.00000068)0.731000:0.00436528,(Acinetobacter_calcoaceticus_gi490781129:0.00
699278,(Acinetobacter_calcoaceticus_gi489748455:0.00000017,(Acinetobacter_calcoa
ceticus_gi491187962:0.00703590,((Acinetobacter_pittii_gi949793812:0.00000001,(Ac
inetobacter_gi507075036:0.00000001,(Acinetobacter_gi491215272:0.00000023,(Acinet
obacter_pittii_gi1043544759:0.00687587,(Acinetobacter_sp._826659_gi727726248:0.0
0688546,(Acinetobacter_calcoaceticus/baumannii_complex_gi516050252:0.00000030,(A
cinetobacter_pittii_gi949728153:0.00685871,(Acinetobacter_gi515954869:0.00688659
,(Acinetobacter_pittii_gi820954340:0.01390214,Acinetobacter_pittii_gi1016519428:
0.00000030)0.000000:0.00000030)0.000000:0.00000040)0.000000:0.00000030)0.000000:
0.00000031)0.000000:0.00000028)0.000000:0.00000023)0.979000:0.02834710)0.000000:
0.00000001)0.983000:0.02795192,((Acinetobacter_baumannii_gi746189085:0.00708547,
(Acinetobacter_nosocomialis_gi549996802:0.00000033,(Acinetobacter_calcoaceticus/
baumannii_complex_gi515962996:0.00693481,(Acinetobacter_calcoaceticus/baumannii_

complex_gi691025008:0.00000001,(Acinetobacter_calcoaceticus/baumannii_complex_gi
691068649:0.00000001,Acinetobacter_calcoaceticus/baumannii_complex_gi487976417:0
.00000035)0.000000:0.00000001)0.000000:0.00000001)0.837000:0.00681397)0.899000:0
.02039532)1.000000:0.18692088,(Acinetobacter_oleivorans_gi502962364:0.00692934,(
Acinetobacter_gi491448944:0.00000021,Acinetobacter_sp._NIPH_817_gi490929625:0.02
125916)0.000000:0.00000021)0.781000:0.00687974)0.000000:0.00000046)0.804000:0.00
697761)0.000000:0.00000016)0.000000:0.00000030)0.739000:0.00448673)0.740000:0.00
511605)0.772000:0.00701877)0.000000:0.00000028)0.881000:0.01432089)0.770000:0.01
423742)1.000000:2.43012758,(((Cupriavidus_sp._amp6_gi916713558:0.29007382,Cupria
vidus_taiwanensis_gi516632550:0.30678487)0.982000:0.50083598,(Ventosimonas_graci
lis_gi1002804265:0.72515483,(((Achromobacter_gi493438632:0.04068728,Achromobacte
r_gi896009906:0.00660654)0.533000:0.01330769,(Achromobacter_gi568194071:0.000000
29,(Achromobacter_sp._gi935635462:0.00664899,(Achromobacter_xylosoxidans_gi99194
3512:0.07281621,Achromobacter_sp._gi935618342:0.00000180)0.783000:0.00720063)0.0
00000:0.00000041)0.000000:0.00000066)0.956000:0.12400087,(Achromobacter_xylosoxi
dans_gi970287464:0.00653050,(Achromobacter_gi849126282:0.00000001,((Achromobacte
r_xylosoxidans_gi651248823:0.00643192,Achromobacter_xylosoxidans_gi529019148:0.0
0000001)0.804000:0.00650716,(Achromobacter_sp._gi935720993:0.00000062,(Achromoba
cter_sp._KCJK1731_gi1028217507:0.04905382,(Achromobacter_xylosoxidans_gi50315831
1:0.02944894,(Bordetella_bronchiseptica_gi980956472:0.01665768,Achromobacter_sp.
_gi935582536:0.01854973)0.935000:0.03894979)0.975000:0.07088004)1.000000:0.23959
144)0.963000:0.04192597)0.000000:0.00000040)0.000000:0.00000001)0.829000:0.04906
737)0.972000:0.34589061)0.816000:0.26000264)0.872000:0.39272924,((Acidovorax_gi9
47497183:0.81336507,(((Pseudomonas_sp._PAMC_26793_gi516047460:0.00559651,(Pseudo
monas_fluorescens_gi1016364294:0.00594800,(Pseudomonas_sp._PAMC_25886_gi49785462
6:0.03707692,(Pseudomonas_fluorescens_gi489310765:0.00000001,Pseudomonas_sp._Ag1
_gi495709086:0.00593182)0.901000:0.01793438)0.904000:0.01800411)0.800000:0.02433
679)0.974000:0.11757273,((Pseudomonas_sp._CHM02_gi647577650:0.02385383,Pseudomon
as_fluorescens_gi734851699:0.01187520)0.695000:0.00507572,(((Pseudomonas_sp._CBZ
-
4_gi648445356:0.06574558,(Pseudomonas_sp._TKP_gi568198333:0.01849600,(Pseudomona
s_sp._KG01_gi881004566:0.03725882,(Pseudomonas_veronii_gi814585896:0.00000753,Ps
eudomonas_veronii_gi817116522:0.03016361)0.988000:0.05225702)0.880000:0.01836380
)0.860000:0.01266276)0.716000:0.01064681,((Pseudomonas_gi489279832:0.00000040,(P
seudomonas_gi734875946:0.00000036,Pseudomonas_fluorescens_gi984660911:0.00600313
)0.000000:0.00000040)0.978000:0.03855704,((Pseudomonas_synxantha_gi492233599:0.0
0000038,(Pseudomonas_sp._BRG-
100_gi696302977:0.00000037,(Pseudomonas_libanensis_gi949578740:0.01885864,Pseudo
monas_gi758892497:0.00000029)0.984000:0.03168531)0.821000:0.00627385)0.985000:0.
03875637,((Pseudomonas_fluorescens_gi515703990:0.00602544,(Pseudomonas_fluoresce
ns_gi966375234:0.01220353,Pseudomonas_sp._Leaf15_gi948200297:0.00607475)0.000000
:0.00000028)0.000000:0.00000027,(Pseudomonas_tolaasii_gi648368667:0.05685249,Pse
udomonas_sp._58_R_3_gi1048635282:0.00000030)0.570000:0.00603830)0.966000:0.03094
348)0.727000:0.00511652)0.954000:0.03891238)0.945000:0.03041761,((Pseudomonas_tr
ivialis_gi903245500:0.00589326,Pseudomonas_sp._58_R_12_gi1048562776:0.00000030)0
.937000:0.01800719,((Pseudomonas_sp._22_E_5_gi1048651134:0.00656478,Pseudomonas_
sp._2-
92_2010__gi655187880:0.00522662)0.952000:0.02946209,((Pseudomonas_sp._RIT357_gi6
96309373:0.03411937,((Pseudomonas_sp._LAMO17WK12:0.00000033,Pseudomonas_sp._8_R_
14_gi1048641023:0.00580611)0.734000:0.00623638,(Pseudomonas_sp._31_E_5_gi1048548
927:0.00566255,Pseudomonas_fluorescens_gi516097813:0.00606677)0.951000:0.0298400
8)0.819000:0.01914879)0.912000:0.02479642,(Pseudomonas_fluorescens_gi742363206:0
.03198211,(Pseudomonas_marginalis_gi1028887139:0.00000026,(Pseudomonas_fluoresce
ns_SBW25_gi229359589:0.00000001,Pseudomonas_fluorescens_gi759483688:0.00000001)0
.000000:0.00000001)0.956000:0.03008267)0.811000:0.01040417)0.879000:0.01236523)0
.733000:0.00599823)0.765000:0.00577571)0.759000:0.00674900)0.946000:0.09458141)0
.948000:0.27143678,((((Pseudomonas_gi495205527:0.00000001,Pseudomonas_chlororaph
is_gi496332433:0.00000001)0.867000:0.02369324,((Pseudomonas_chlororaphis_gi51507
5193:0.00000001,(Pseudomonas_chlororaphis_gi496337189:0.00000001,Pseudomonas_chl
ororaphis_gi740797859:0.01220531)0.000000:0.00000001)0.760000:0.00605626,(Pseudo

monas_chlororaphis_gi565888759:0.01834671,(Pseudomonas_chlororaphis_gi655271948:
0.00000016,Pseudomonas_chlororaphis_gi754595179:0.00606211)0.000000:0.00000016)0
.741000:0.00611954)0.924000:0.04116166)1.000000:0.27356910,((Pseudomonas_batumic
i_gi749012760:0.09112193,(Pseudomonas_gingeri_gi515692155:0.08258973,(Pseudomona
s_fuscovaginae_gi929037659:0.00000055,Pseudomonas_fuscovaginae_gi929038886:0.006
30327)1.000000:0.15538291)0.320000:0.04600725)0.999000:0.18531031,(((Pseudomonas
_fluorescens_gi1042202568:0.04605011,((Pseudomonas_sp._MS586_gi1033072250:0.0260
3281,Pseudomonas_fluorescens_gi499651157:0.00000028)0.942000:0.03271716,(Pseudom
onas_sp._GM25_gi495232871:0.00000026,Pseudomonas_gi748202076:0.00622977)0.730000
:0.00743694)0.990000:0.07118741)0.632000:0.02158510,((Pseudomonas_sp._H1h_gi6406
89021:0.03338176,(Pseudomonas_fluorescens_gi829057053:0.00599684,(Pseudomonas_sp
._W15Feb9B_gi751602866:0.00602914,Pseudomonas_fluorescens_gi489313099:0.06854024
)0.000000:0.00000039)0.776000:0.01276163)0.940000:0.03205514,(((Pseudomonas_gi75
2848418:0.01238721,(Pseudomonas_fluorescens_gi748185190:0.03208996,(Pseudomonas_
sp._RIT-PI-
o_gi1033049985:0.00000001,Pseudomonas_gi756161663:0.01193628)0.959000:0.03140325
)0.595000:0.00528499)0.910000:0.01863813,(Pseudomonas_moraviensis_gi566142311:0.
00000028,Pseudomonas_gi515143551:0.00608862)0.000000:0.00000062)0.995000:0.05692
699,(Pseudomonas_sp._GM80_gi495355574:0.01630726,(Pseudomonas_fluorescens_gi7556
91502:0.07262628,(Pseudomonas_sp._RIT-PI-
r_gi1033078951:0.01440757,Pseudomonas_fluorescens_gi734952039:0.01028513)0.76000
0:0.01191436)0.756000:0.00851396)0.773000:0.01488989)0.839000:0.02152055)0.22800
0:0.01080841)0.953000:0.08547418,(Pseudomonas_sp._A3_2016__gi1036108341:0.326448
05,((Pseudomonas_fluorescens_gi639433323:0.00000001,Pseudomonas_sp._CFII68_gi517
435696:0.00000001)0.994000:0.09417498,(Pseudomonas_brassicacearum_gi643458054:0.
04503956,Pseudomonas_sp._SHC52_gi751316590:0.05677380)0.612000:0.01996699)0.9860
00:0.13918683)0.168000:0.03573807)0.988000:0.15545901)0.316000:0.03426289)0.8200
00:0.04051007,(Pseudomonas_gi764693149:0.13296813,(Pseudomonas_fluorescens_gi829
067488:0.07920958,(Pseudomonas_sp._CMAA1215_gi550107232:0.15888394,(Pseudomonas_
fluorescens_group_gi499370968:0.00000001,(Pseudomonas_fluorescens_gi829611363:0.
00000001,Pseudomonas_protegens_gi751648199:0.00585904)0.000000:0.00000001)0.8860
00:0.02676139)0.144000:0.01058112)0.938000:0.07014243)0.974000:0.11485604)0.9640
00:0.29128875)0.998000:0.51748852)0.769000:0.13473752,((Alcanivorax_gi552528757:
0.50514536,((Trabulsiella_odontotermitis_gi908733472:0.08590081,(Kosakonia_oryza
e_gi1034668588:0.00000001,Kosakonia_oryzae_gi1035732114:0.00000001)0.987000:0.17
068370)0.982000:0.23380610,((Escherichia_hermannii_gi488365880:0.00000001,Entero
bacter_gi760379958:0.00609623)1.000000:0.31073947,((Citrobacter_gi851904785:0.01
303551,Citrobacter_pasteurii_gi491265229:0.00547640)0.633000:0.00880464,((Citrob
acter_braakii_gi896303566:0.00000001,(Citrobacter_gi507083556:0.00000001,(Entero
bacteriaceae_gi808791665:0.00000020,(Citrobacter_sp._KTE32_gi507086656:0.0061957
4,(Citrobacter_braakii_gi896286137:0.00619431,Citrobacter_freundii_complex_gi895
958854:0.00000001)0.000000:0.00000036)0.000000:0.00000029)0.000000:0.00000020)0.
000000:0.00000001)0.969000:0.02539037,(Citrobacter_freundii_gi696375470:0.000000
32,(Citrobacter_freundii_complex_gi489937688:0.00000021,(Citrobacter_gi489124060
:0.00000001,(Citrobacter_sp._AATXQ_gi1004662917:0.00636663,(Citrobacter_freundii
_gi560879375:0.00618329,((Citrobacter_sp._MGH105_gi851912059:0.01238383,Enteroba
cteriaceae_gi489922192:0.00000001)0.875000:0.01164944,(((Citrobacter_youngae_gi4
93737737:0.00614918,(Salmonella_enterica_gi1002470780:0.00000001,Enterobacteriac
eae_gi754954695:0.00000027)0.000000:0.00000001)0.779000:0.00621436,(Enterobacter
_cloacae_complex_gi742935143:0.00624166,(Enterobacteriaceae_gi735677192:0.000000
01,(Enterobacter_cloacae_complex_gi949716453:0.00618260,(Citrobacter_gi780038509
:0.02558094,(Plesiomonas_sp._ZOR0011_gi835946988:0.02099682,Plesiomonas_shigello
ides_gi743527888:0.06684360)1.000000:0.41144470)0.841000:0.06516656)0.000000:0.0
0000029)0.000000:0.00000001)0.889000:0.01242515)0.874000:0.01250922,(Citrobacter
_freundii_gi757800259:0.01871166,Citrobacter_freundii_gi835232286:0.00621711)0.7
71000:0.00609617)0.793000:0.00803677)0.760000:0.00512793)0.000000:0.00000030)0.0
00000:0.00000033)0.000000:0.00000041)0.920000:0.00618058)0.000000:0.00000032)0.8
38000:0.02969920)0.996000:0.26303901)0.795000:0.08188504)0.787000:0.06818584)0.9
09000:0.18294579,((Erwinia_sp._Leaf53_gi947571652:0.53762935,(Xenorhabdus_cabani
llasii_gi740429284:0.18312566,((Xenorhabdus_szentirmaii_DSM_16338_gi575845541:0.

00000001,Xenorhabdus_szentirmaii_gi740407660:0.00000001)1.000000:0.26632745,(Xen
orhabdus_khoisanae_gi847177187:0.14853799,(Xenorhabdus_doucetiae_gi800943138:0.2
1072307,(Xenorhabdus_nematophila_gi754590960:0.00000036,(Xenorhabdus_nematophila
_gi498911576:0.01311308,Xenorhabdus_nematophila_gi502949675:0.00000001)0.807000:
0.00662244)0.980000:0.11957039)0.474000:0.02089606)0.122000:0.01703129)0.792000:
0.09048108)0.991000:0.39175038)0.749000:0.13419447,((Yersinia_entomophaga_gi1035
666203:0.02339109,Yersinia_nurmii_gi902506983:0.01785641)0.990000:0.31068203,((Y
ersinia_gi499505727:0.00000032,(Yersinia_pestis_gi514842357:0.00000001,((((Yersi
nia_pseudotuberculosis_gi910821874:0.00581249,Yersinia_pseudotuberculosis_comple
x_gi727729110:0.00000029)0.950000:0.01760779,(((Yersinia_pestis_gi1012086256:0.0
0000001,Yersinia_pestis_gi914241203:0.00640745)0.000000:0.00011325,(Yersinia_pse
udotuberculosis_complex_gi488140833:0.00000001,Yersinia_pestis_S3_gi550622586:0.
00583818)0.000000:0.00000029)0.000000:0.00000032,(Yersinia_pestis_gi514869780:0.
00000001,(Yersinia_pestis_gi929988362:0.00583906,(Yersinia_pseudotuberculosis_gi
912958931:0.00583990,Yersinia_pestis_gi514985608:0.00000034)0.000000:0.00000040)
0.000000:0.00000030)0.000000:0.00000001)0.000000:0.00000018)0.000000:0.00000038,
(Yersinia_pestis_gi1016395499:0.00598362,Yersinia_pestis_24H_gi550623175:0.00000
025)0.000000:0.00000038)0.000000:0.00000039,(Yersinia_pestis_gi514985947:0.00000
001,Yersinia_pseudotuberculosis_gi742321036:0.00583483)0.000000:0.00000029)0.000
000:0.00000001)0.816000:0.00583046)0.856000:0.04944945,((Yersinia_intermedia_ATC
C_29909_gi238728145:0.00914598,(Yersinia_intermedia_gi696330984:0.00000017,(Yers
inia_gi910778589:0.00564860,Yersinia_intermedia_gi915759571:0.00000023)0.938000:
0.01132371)0.000000:0.00000017)0.859000:0.02836573,(((Yersinia_mollaretii_gi9025
36937:0.00000001,(Yersinia_enterocolitica_gi910939633:0.00000029,(Yersinia_molla
retii_gi902774849:0.00587860,(Yersinia_mollaretii_gi696235012:0.00000001,Yersini
a_mollaretii_ATCC_43969_gi238718316:0.00000085)0.798000:0.00583770)0.806000:0.00
578931)0.395000:0.00581499)0.861000:0.01550477,((Yersinia_gi696321441:0.00174305
,Yersinia_bercovieri_ATCC_43970_gi238715819:0.00439875)0.993000:0.04763034,(Yers
inia_gi873914047:0.02367022,(Yersinia_frederiksenii_gi910775309:0.01748430,(Yers
inia_gi518039521:0.00000026,Yersinia_massiliensis_gi902536492:0.00572027)0.00000
0:0.00000067)1.000000:0.06717816)0.000000:0.00000028)0.814000:0.01400007)0.91600
0:0.02916178,(Yersinia_pekkanenii_gi902545460:0.05735284,(((Yersinia_frederiksen
ii_gi912864704:0.00000033,(Yersinia_frederiksenii_ATCC_33641_gi238724779:0.00588
096,(Yersinia_frederiksenii_gi910807563:0.02945814,Yersinia_frederiksenii_gi6963
34819:0.00000030)0.000000:0.00000030)0.789000:0.00575818)0.758000:0.01165984,(Ye
rsinia_frederiksenii_gi910795409:0.01218211,(Yersinia_rohdei_gi914218853:0.00000
001,(Yersinia_rohdei_ATCC_43380_gi238710237:0.00689890,Yersinia_rohdei_gi6962373
70:0.00000001)0.808000:0.00571944)1.000000:0.12724179)0.633000:0.01963459)0.7690
00:0.01547768,(((Yersinia_aldovae_gi902760297:0.01203916,((Yersinia_aldovae_gi75
7587927:0.00000030,Yersinia_aldovae_ATCC_35236_gi238704724:0.00000059)0.000000:0
.00000031,(Yersinia_aldovae_gi902969381:0.00000001,Yersinia_aldovae_gi755640855:
0.00566818)0.858000:0.00567726)0.736000:0.00571782)0.997000:0.06591630,(Yersinia
_enterocolitica_LC20_gi595641327:0.02388600,((Yersinia_kristensenii_gi898207783:
0.00000029,(Yersinia_kristensenii_ATCC_33638_gi238698799:0.00606616,(Yersinia_kr
istensenii_gi696240338:0.00000037,Yersinia_kristensenii_gi951154643:0.00566965)0
.000000:0.00000037)0.776000:0.00567364)0.776000:0.01173417,(Yersinia_gi740848293
:0.01123952,Yersinia_kristensenii_gi910814893:0.02306745)0.000000:0.00000192)0.9
70000:0.02928740)0.000000:0.00000050)0.490000:0.01688517,(Yersinia_intermedia_gi
910768375:0.00000001,(Yersinia_enterocolitica_gi545670615:0.00000089,((Yersinia_
enterocolitica_gi491306391:0.00000035,(Yersinia_enterocolitica_gi503415315:0.005
86133,(Yersinia_enterocolitica_gi896647613:0.00586495,Yersinia_enterocolitica_gi
901654805:0.00000001)0.000000:0.00002959)0.000000:0.00000024)0.897000:0.01175999
,(Yersinia_enterocolitica_gi491311018:0.00580621,(Yersinia_enterocolitica_gi3544
64702:0.01165795,(Yersinia_enterocolitica_gi554498950:0.00582251,Yersinia_entero
colitica_gi517909000:0.00000001)0.000000:0.00000001)0.000000:0.00000001)0.000000
:0.00000026)0.836000:0.00581875)0.000000:0.00000001)0.755000:0.00678958)0.896000
:0.02113371)0.791000:0.02108703)0.150000:0.02401310)0.666000:0.02456138)0.915000
:0.09162812)0.592000:0.18441126)0.994000:0.43326409)0.537000:0.12272492)0.963000
:0.26861611)0.845000:0.18246738)0.454000:0.22203395)0.815000:0.31890617)0.999000
:1.26522296);

pHoH

(Hafnia_alvei_gi748798962:0.00762614,Enterobacteriaceae_gi647478235:0.0000
0012,(Hafnia_gi490192880:0.00762452,(Obesumbacterium_proteus_ATCC_12841_gi103398
8530:0.00000015,(((Serratia_gi739538377:0.01544208,Serratia_gi544754079:0.015565
36)0.757000:0.00783059,(Chania_multitudinisentens_gi640471457:0.03154805,(((Yers
inia_pekkanenii_gi902545454:0.04702372,((Yersinia_enterocolitica_gi896647611:0.0
0767640,(Yersinia_gi491311026:0.00000001,((Yersinia_enterocolitica_gi951139468:0
.00737503,((Yersinia_gi518039523:0.00739795,(Yersinia_kristensenii_gi910814895:0
.00737484,Yersinia_gi491414106:0.00000013)0.000000:0.00000013)0.000000:0.0000005
6,(Yersinia_gi696330985:0.00743146,(((Yersinia_rohdei_ATCC_43380_gi238710239:0.0
0743734,Yersinia_rohdei_gi696237374:0.00000001)0.755000:0.00739326,(Yersinia_ald
ovae_gi755640851:0.00000001,Yersinia_aldovae_ATCC_35236_gi238704726:0.00733056)0
.937000:0.02294210)0.769000:0.00739395,(Yersinia_gi696235015:0.00000057,(Yersini
a_frederiksenii_gi696334822:0.00000022,Yersinia_frederiksenii_gi912948507:0.0074
0465)0.850000:0.00740168)0.850000:0.00739142)0.000000:0.00000031)0.000000:0.0000
0059)0.842000:0.00737586)0.000000:0.00000025,(Yersinia_kristensenii_gi912845647:
0.00737091,(Yersinia_kristensenii_ATCC_33638_gi238698796:0.00743825,(Yersinia_en
terocolitica_gi554499276:0.00738536,(Yersinia_enterocolitica_gi491306378:0.00000
001,Yersinia_enterocolitica_gi504421800:0.00738346)0.000000:0.00000052)0.853000:
0.00738350)0.000000:0.00000074)0.000000:0.00000035)0.000000:0.00000001)0.000000:
0.00000028)0.928000:0.01554604,(Yersinia_enterocolitica_LC20_gi595641330:0.01566
615,((Yersinia_entomophaga_gi1035666210:0.00755906,Yersinia_nurmii_gi902506986:0
.00000026)0.722000:0.00701394,(Yersinia_ruckeri_gi490858559:0.00000089,Yersinia_
ruckeri_gi755595948:0.00729827)0.933000:0.03177476)0.936000:0.04116590)0.718000:
0.00737344)0.000000:0.00000071)0.771000:0.00843842,(Serratia_sp._DD3_gi558560732
:0.11800910,(Yersinia_gi488140829:0.00000001,(Yersinia_pestis_PY-
25_gi391511972:0.00736348,(Yersinia_pseudotuberculosis_complex_gi644962944:0.014
93718,(Yersinia_pseudotuberculosis_gi950887733:0.00733249,(Yersinia_pseudotuberc
ulosis_complex_gi910788082:0.00000001,Yersinia_wautersii_gi757561251:0.00734244)
0.000000:0.00000051)0.839000:0.00733516)0.000000:0.00000051)0.000000:0.00000065)
0.691000:0.00586108)0.506000:0.01629047)0.903000:0.02978238,((Sodalis_praecaptiv
us_gi645050568:0.20015144,((Pectobacterium_carotovorum_gi497991968:0.00000093,(P
ectobacterium_carotovorum_gi746431185:0.00000019,(Pectobacterium_carotovorum_gi7
46313006:0.00771430,(Pectobacterium_carotovorum_gi506320989:0.00776618,((Pectoba
cterium_atrosepticum_gi746261596:0.01587045,(Pectobacterium_gi492808258:0.000000
16,(Pectobacterium_betavasculorum_gi746250417:0.01559437,(Pectobacterium_carotov
orum_gi852028282:0.00776777,(Pectobacterium_atrosepticum_gi499405851:0.00778784,
Pectobacterium_atrosepticum_gi746244111:0.00000085)0.905000:0.01580909)0.000000:
0.00000085)0.000000:0.00000052)0.960000:0.03169952)0.762000:0.00782948,(Pectobac
terium_carotovorum_gi746482301:0.00766184,(Pectobacterium_carotovorum_gi74622446
1:0.01542284,((Pectobacterium_carotovorum_gi746517738:0.00000078,Pectobacterium_
carotovorum_gi746445707:0.03940666)0.822000:0.00768163,(Pectobacterium_carotovor
um_gi746511973:0.00767671,Pectobacterium_carotovorum_gi497965507:0.0000087)0.00
0000:0.00000029)0.000000:0.00000051)0.000000:0.00000068)0.817000:0.00770700)0.00
0000:0.00000095)0.000000:0.00000091)0.000000:0.00000019)0.794000:0.00772317)0.97
8000:0.07288999,(Brenneria_sp._EniD312_gi496404315:0.02087441,(Brenneria_goodwin
ii_gi873961242:0.01053198,((Serratia_sp._ATCC_39006_gi542100241:0.10261961,(Lons
dalea_quercina_gi652343921:0.00426523,Lonsdalea_quercina_gi652345829:0.01901881)
0.992000:0.14871929)0.325000:0.02640605,(Dickeya_paradisiaca_gi502328981:0.15999
596,(Dickeya_sp._2B12_gi723589180:0.00000053,(Dickeya_dianthicola_gi568602367:0.
01592475,(Dickeya_dadantii_gi503083602:0.00000031,((Dickeya_dadantii_gi742466386
:0.01581930,((Enterobacteriaceae_gi740882713:0.00000001,Dickeya_solani_gi7467389
40:0.00764428)0.793000:0.00780276,(Dickeya_gi742462706:0.00261921,(Dickeya_solan
i_gi550651097:0.00000095,Dickeya_solani_gi949753066:0.00764648)0.963000:0.031493
42)0.646000:0.00585532)0.801000:0.00793050)0.000000:0.00000038,(Dickeya_sp._NCPP
B_3274_gi757615833:0.00777179,((Dickeya_sp._NCPPB_569_gi757631428:0.03639595,((D
ickeya_zeae_gi515507620:0.02329944,(Dickeya_zeae_gi742425805:0.00757670,Dickeya_
zeae_gi742468895:0.00000079)0.780000:0.00752708)0.776000:0.00798818,(Dickeya_zea
e_gi518682114:0.00000058,Dickeya_zeae_gi502647936:0.00779879)0.699000:0.00775915
)0.000000:0.00000098)0.756000:0.00801425,(Dickeya_chrysanthemi_gi502350036:0.015

63031,(Dickeya_chrysanthemi_gi748743344:0.02328241,Dickeya_chrysanthemi_gi654080
960:0.00000077)0.660000:0.01596332)0.972000:0.04218186)0.836000:0.01576183)0.771
000:0.00782725)0.000000:0.00000030)0.780000:0.00776097)0.811000:0.00784550)0.846
000:0.03652716)0.905000:0.05268577)0.955000:0.08554170)0.923000:0.05249204)0.684
000:0.00662997)0.964000:0.09011322)0.971000:0.09509653,(((Rahnella_aquatilis_gi5
05735850:0.00000052,Serratia_sp._Leaf51_gi948111730:0.01558418)0.889000:0.023137
04,((Ewingella_americana_gi736787812:0.05127932,Rahnella_gi503341103:0.02880047)
0.913000:0.03215931,(Rouxiella_chamberiensis_gi769913352:0.02166295,(Serratia_sp
._M24T3_gi497322043:0.00000059,(gamma_proteobacterium_WG36_gi516060700:0.0601294
0,Serratia_sp._Leaf50_gi946856913:0.00000110)0.847000:0.02323145)0.941000:0.0316
2651)0.952000:0.03590883)0.261000:0.00613764)0.922000:0.04124792,((Cedecea_gi514
233130:0.00000093,(Cedecea_neteri_gi746243309:0.00790623,Enterobacteriaceae_gi49
5731062:0.00000057)0.346000:0.00789711)0.862000:0.03482064,(((Buttiauxella_agres
tis_gi917273851:0.00000001,(Buttiauxella_gaviniae_gi1035670784:0.01620446,(Butti
auxella_noackiae_gi1035713226:0.00000001,Buttiauxella_noackiae_ATCC_51607_gi1033
947132:0.00000028)0.000000:0.00000002)0.000000:0.00000062)0.871000:0.02400255,(B
uttiauxella_ferragutiae_gi1035706606:0.01571375,(Buttiauxella_agrestis_gi7364727
36:0.00782080,Buttiauxella_brennerae_gi1035718225:0.00772945)0.775000:0.00818577
)0.000000:0.00000053)0.908000:0.03601538,(((Cronobacter_muytjensii_gi742384552:0
.00000058,(Cronobacter_dublinensis_gi495021614:0.00785579,((Cronobacter_malonati
cus_gi696395369:0.00000001,(Cronobacter_sakazakii_gi742380776:0.00772472,((Crono
bacter_universalis_NCTC_9529_gi426510107:0.00000053,(Cronobacter_sakazakii_CMCC_
45402_gi564118599:0.00000061,Cronobacter_sakazakii_gi752821961:0.00774868)0.2740
00:0.00770111)0.856000:0.00775093,(Cronobacter_gi490522141:0.00000001,Cronobacte
r_malonaticus_gi696413147:0.00770938)0.000000:0.00000040)0.000000:0.00000033)0.0
00000:0.00000085)0.000000:0.00000079,(Cronobacter_turicensis_gi754512751:0.00000
030,(Cronobacter_turicensis_gi696225944:0.04174592,(Cronobacter_condimenti_gi494
948489:0.00789546,Cronobacter_turicensis_z3032_gi260216574:0.00000041)0.000000:0
.00000052)0.000000:0.00000074)0.725000:0.00775536)0.428000:0.00832441)0.879000:0
.01577101)0.990000:0.06820646,(Franconibacter_helveticus_gi639216874:0.00000001,
Enterobacteriaceae_gi639222054:0.00759427)0.924000:0.03762407)0.759000:0.0086684
3,(((Enterobacteriaceae_bacterium_LSJC7_gi515941374:0.19811217,Erwinia_sp._SCU-
B244_gi971843784:0.07057374)0.131000:0.02408936,Shimwellia_blattae_gi488370264:0
.26597373)0.865000:0.05381150,((Pluralibacter_gergoviae_gi759354029:0.03913524,(
Escherichia_gi446455676:0.00000059,(Escherichia_sp._B1147_gi1035684356:0.0155910
7,((((Escherichia_coli_gi446180898:0.00000001,(Shigella_dysenteriae_CDC_74-
1112_gi320176600:0.00000001,Shigella_dysenteriae_gi491184536:0.00000001)0.919000
:0.00798328)0.000000:0.00000001,((Enterobacteriaceae_gi446455678:0.00000001,(Ent
erobacteriaceae_gi446455667:0.00000030,(Proteobacteria_gi446180910:0.00000001,(P
hoH:0.00000001,(Escherichia_coli_SE11_gi209911535:0.00000001,((((Shigella_sonnei
_gi920314873:0.00000001,Shigella_sonnei_gi904579011:0.00000001)0.917000:0.008042
76,((Shigella_sonnei_gi920200385:0.00000001,Shigella_sonnei_gi904010240:0.000000
01)0.839000:0.00803458,(Shigella_sonnei_gi920316803:0.00000001,Shigella_sonnei_g
i904261211:0.00000001)0.846000:0.00803165)0.000000:0.00000053)0.000000:0.0000004
8,(Shigella_sonnei_Ss046_gi73855060:0.00000001,(Shigella_sonnei_gi904422313:0.00
000001,(Shigella_sonnei_gi491281808:0.00000001,((Shigella_sonnei_gi920542804:0.0
0000001,Shigella_sonnei_gi903713451:0.00000001)0.000000:0.00803078,Shigella_sonn
ei_gi903799502:0.03010136)0.000000:0.00000090)0.000000:0.00000090)0.000000:0.000
00090)0.000000:0.00000092)0.000000:0.00000091,(Shigella_sonnei_gi903532614:0.011
74827,(Shigella_sonnei_gi920564126:0.00000001,Shigella_sonnei_gi904952331:0.0000
0001)0.850000:0.00803495)0.000000:0.00000097)0.914000:0.00804458)0.000000:0.0000
0040)0.000000:0.00000083)0.000000:0.00000084)0.915000:0.00801768)0.000000:0.0000
0088,(Shigella_boydii_Sb227_gi81245913:0.00000001,(Shigella_flexneri_K-
315_gi391266261:0.02421426,(Shigella_flexneri_gi491243403:0.00000078,(Shigella_f
lexneri_gi499381753:0.00000001,((Shigella_flexneri_gi491189043:0.00000001,Shigel
la_flexneri_gi684117996:0.00802414)0.000000:0.00000084,(Shigella_flexneri_gi7631
44621:0.00804153,Shigella_flexneri_gi491236989:0.00795341)0.000000:0.00000090)0.
000000:0.00000077)0.842000:0.00797835)0.883000:0.01611177)0.000000:0.00000069)0.
000000:0.00000001)0.000000:0.00000090)0.856000:0.00806410,((Escherichia_ferguson
ii_gi803575875:0.00795709,(Escherichia_fergusonii_ATCC_35469_gi218356789:0.00000

033,(Escherichia_fergusonii_gi446455683:0.00000001,Escherichia_fergusonii_B253_g
i324114235:0.00000001)0.921000:0.00801707)0.000000:0.00000033)0.828000:0.0079812
7,(Escherichia_coli_B354_gi291469870:0.00000040,(Escherichia_gi507087304:0.00795
858,Escherichia_gi446455681:0.00000001)0.000000:0.00000071)0.000000:0.00000091)0
.000000:0.00000070)0.756000:0.00795740,((Escherichia_fergusonii_ECD227_gi3254976
66:0.00000001,Escherichia_fergusonii_gi446455682:0.00000001)0.837000:0.00809342,
(Escherichia_albertii_gi974656669:0.00802982,((Escherichia_albertii_gi974627883:
0.01635155,(Escherichia_albertii_gi974684970:0.00000062,(Escherichia_albertii_gi
974693069:0.00801952,Escherichia_albertii_gi949426411:0.00000001)0.000000:0.0000
0097)0.835000:0.00802941)0.000000:0.00000092,(Escherichia_albertii_gi446455684:0
.00802041,(Escherichia_albertii_gi949421844:0.00000001,(Escherichia_albertii_gi9
53765017:0.00000001,(Escherichia_albertii_KF1_gi569536004:0.00000001,(Escherichi
a_albertii_gi446455677:0.00000001,Escherichia_albertii_NBRC_107761_gi689833295:0
.00000001)0.921000:0.00802198)0.000000:0.00000072)0.000000:0.00000094)0.000000:0
.00000090)0.000000:0.00000090)0.000000:0.00000080)0.833000:0.00808511)0.000000:0
.00000059)0.760000:0.00787364)0.743000:0.00912727)0.962000:0.04700694)0.608000:0
.00828066,((((Citrobacter_koseri_gi501082330:0.00000083,Citrobacter_koseri_gi835
329865:0.00766056)0.871000:0.02337823,((Citrobacter_gi780025207:0.00779207,(Salm
onella_enterica_gi1002399062:0.00763888,(((Citrobacter_freundii_gi817709222:0.00
765868,Citrobacter_gi489126255:0.00000001)0.000000:0.00000068,(Citrobacter_freun
dii_NBRC_12681_gi689842487:0.00000032,(Citrobacter_freundii_gi835237189:0.007634
94,(Citrobacter_gi491263968:0.01529057,Enterobacteriaceae_gi507081842:0.00762744
)0.000000:0.00000046)0.000000:0.00000081)0.000000:0.00000068)0.912000:0.00762651
,(Citrobacter_freundii_complex_gi493735975:0.00000045,(Citrobacter_sp._30_2_gi22
6906503:0.00000087,(Enterobacteriaceae_gi489928751:0.00000001,(Citrobacter_sp._M
GH110_gi851967518:0.00758829,Citrobacter_freundii_complex_gi696367930:0.00000001
)0.851000:0.00771419)0.000000:0.00000081)0.910000:0.00762293)0.000000:0.00000076
)0.000000:0.00000055)0.855000:0.01522215)0.947000:0.03624873,((Gammaproteobacter
ia_gi764284429:0.00747001,(Citrobacter_gi757782452:0.00000024,Citrobacter_sp._Ct
B7.12_gi930176530:0.00745375)0.000000:0.00000060)0.802000:0.01466128,((Citrobact
er_gi754931065:0.03910990,Citrobacter_rodentium_gi582046576:0.00000147)0.953000:
0.03975915,(Citrobacter_farmeri_gi754963816:0.00000001,(Citrobacter_farmeri_GTC_
1319_gi693468831:0.00000001,(Citrobacter_amalonaticus_gi992390147:0.00592905,Cit
robacter_amalonaticus_gi817105597:0.01274603)0.605000:0.00333114)0.000000:0.0000
0001)0.779000:0.00733743)0.729000:0.01039318)0.776000:0.01604706)0.799000:0.0219
2881)0.849000:0.02304480,(((Erwinia_gerundensis_gi972720984:0.09900403,((((Panto
ea_gi495168915:0.00786365,Enterobacteriaceae_gi495380608:0.00000095)0.899000:0.0
3014878,(Enterobacteriaceae_gi746284367:0.01588790,Enterobacteriaceae_gi73682270
5:0.03348296)0.725000:0.01041041)0.922000:0.03530655,((Pantoea_eucrina_gi1046477
717:0.00789751,Pantoea_sp._PSNIH1_gi746339768:0.00000078)0.987000:0.06016291,(Pa
ntoea_dispersa_gi970479762:0.00000060,Pantoea_dispersa_gi545154621:0.00788703)0.
729000:0.00667314)0.813000:0.01567742)0.917000:0.03892513,(Pantoea_sp._A4_gi5159
15989:0.07290685,((Pantoea_stewartii_gi493070478:0.00000099,(Pantoea_gi502791149
:0.00803552,(Pantoea_sp._3.5.1_gi666663961:0.00701505,(Pantoea_gi496380655:0.000
00103,(Pantoea_agglomerans_gi992367698:0.01669425,(Pantoea_gi727274785:0.0000006
1,(Pantoea_gi489948977:0.00000062,(Pantoea_gi497944349:0.00000046,Pantoea_agglom
erans_gi746346231:0.00813830)0.933000:0.01682606)0.821000:0.00810067)0.755000:0.
00799774)0.957000:0.03354544)0.928000:0.02451371)0.786000:0.00930698)0.716000:0.
00812409)0.868000:0.02804744,((Pantoea_vagans_gi881068207:0.00846759,(Pantoea_sp
._FF5_gi727334914:0.00795357,Pantoea_sp._NGS-ED-
1003_gi727293315:0.00000088)0.751000:0.00791744)0.924000:0.04904680,((Tatumella_
saanichensis_gi658473568:0.10472077,(Tatumella_morbirosei_gi740178169:0.06250725
,Tatumella_gi647629044:0.06477022)0.598000:0.02776451)0.998000:0.18688528,(Panto
ea_sp._PSNIH2_gi740840259:0.09956924,Pantoea_gi503274855:0.04054047)0.249000:0.0
1923726)0.183000:0.01162697)0.074000:0.00444154)0.861000:0.02370646)0.732000:0.0
0967255)0.838000:0.02808226)0.889000:0.03366090,((Erwinia_persicina_gi1011934045
:0.05674082,((Erwinia_oleae_gi736954214:0.00715029,Erwinia_sp._9145_gi736917820:
0.00829536)0.952000:0.04904318,(((Erwinia_pyrifoliae_gi501912348:0.00768110,(Erw
inia_amylovora_gi490260865:0.00777331,(Erwinia_sp._Ejp617_gi504357405:0.00767154
,Erwinia_amylovora_gi490274030:0.00767719)0.000000:0.00000078)0.000000:0.0000004

9)0.898000:0.03349046,(Erwinia_pirifloriniigrans_gi562748239:0.01375600,Erwinia_t
asmaniensis_gi501409609:0.00469504)0.866000:0.03218515)0.933000:0.04497806,(Pant
oea_sp._IMH_gi640531082:0.05208866,((Erwinia_tracheiphila_gi520900516:0.02511670
,Erwinia_mallotivora_gi736938688:0.02350912)0.871000:0.02594171,(Erwinia_billing
iae_gi502967663:0.02367085,(Erwinia_typographi_gi736888163:0.02410668,bacteria_s
ymbiont_BFo1_of_Frankliniella_occidentalis_gi887509529:0.04025843)0.731000:0.008
33965)0.884000:0.03120535)0.826000:0.02718353)0.757000:0.01195736)0.555000:0.021
35435)0.759000:0.01911310)0.911000:0.04403564,(Erwinia_toletana_gi516410630:0.00
771875,(Erwinia_sp._Leaf53_gi947570644:0.05039858,Erwinia_iniecta_gi919886884:0.
02253739)0.823000:0.01749120)0.753000:0.01643787)0.776000:0.01392977)0.986000:0.
07650095,((((Salmonella_enterica_gi446455674:0.00000001,Salmonella_enterica_subs
p._enterica_serovar_Paratyphi_C_str._RKS4594_gi224468894:0.00000001)0.926000:0.0
0775788,(Salmonella_enterica_gi912572710:0.00775939,Salmonella_enterica_gi959102
345:0.00775900)0.000000:0.00000091)0.000000:0.00000035,(((Salmonella_enterica_gi
446455675:0.00000055,(Salmonella_enterica_gi487404397:0.00779388,(Salmonella_ent
erica_gi555251366:0.00000001,Salmonella_enterica_subsp._enterica_serovar_Cholera
esuis_str._SC-
B67_gi62127280:0.00000001)0.860000:0.00776108)0.000000:0.00000084)0.000000:0.000
00047,((Salmonella_enterica_gi446455671:0.00000001,(Salmonella_enterica_subsp._e
nterica_serovar_Infantis_str._SARB27_gi353075471:0.00000001,Salmonella_enterica_
gi582112438:0.00000001)0.832000:0.00793495)0.964000:0.02405212,((Salmonella_ente
rica_gi1002388757:0.00776609,((Salmonella_enterica_gi810443241:0.00776134,(Salmo
nella_enterica_gi446455673:0.00000001,(Salmonella_enterica_subsp._enterica_serov
ar_Enteritidis_str._P125109_gi206708270:0.00000001,(Salmonella_enterica_gi582971
799:0.00000001,Salmonella_enterica_subsp._enterica_serovar_Pullorum_gi661552077:
0.00000001)0.920000:0.00775398)0.000000:0.00000001)0.000000:0.00000001)0.000000:
0.00000046,Salmonella_enterica_gi972328382:0.00776035)0.000000:0.00777764)0.0000
00:0.00000056,(Salmonella_enterica_gi555262293:0.00000031,Salmonella_enterica_su
bsp._enterica_serovar_Montevideo_str._USDA-ARS-USMARC-
1903_gi764054862:0.00782677)0.000000:0.00000039)0.000000:0.00000044)0.000000:0.0
0000076)0.000000:0.00000070,(Salmonella_enterica_gi446455672:0.00000001,(Salmone
lla_enterica_gi446455670:0.00776099,((Salmonella_enterica_gi554956647:0.00775063
,(Salmonella_enterica_subsp._enterica_serovar_Typhimurium_gi902850923:0.00000041
,(Salmonella_enterica_gi554960917:0.00000141,Salmonella_enterica_gi916060633:0.0
0783735)0.712000:0.00775643)0.000000:0.00000047)0.000000:0.00000041,(Salmonella_
enterica_subsp._enterica_serovar_Montevideo_str._S5-
403_gi353634195:0.00000001,(Salmonella_enterica_gi486434906:0.00000001,(Salmonel
la_enterica_gi916179505:0.00775990,Salmonella_enterica_gi950832650:0.00777060)0.
000000:0.00000059)0.000000:0.00000001)0.000000:0.00000001)0.000000:0.00000067)0.
000000:0.00000079)0.000000:0.00000001)0.000000:0.00000052)0.979000:0.05036478,((
Salmonella_enterica_gi554683544:0.00000083,(Salmonella_bongori_NCTC_12419_gi3395
12333:0.00000001,Salmonella_bongori_gi446455664:0.00000001)0.999000:0.06395026)0
.799000:0.00767890,(Salmonella_enterica_gi960873356:0.00751755,(Salmonella_enter
ica_gi446455665:0.00000010,(Salmonella_enterica_gi489040178:0.00752666,Salmonell
a_enterica_gi446455668:0.00000061)0.859000:0.00752640)0.000000:0.00000053)0.0000
00:0.00000062)0.730000:0.00787095)0.945000:0.04526123)0.766000:0.01438847)0.7280
00:0.00921260,((Enterobacteriaceae_gi516025706:0.00000094,((Enterobacter_cloacae
_gi558089346:0.03219787,(Kosakonia_radicincitans_gi494616107:0.00775758,(Kosakon
ia_radicincitans_YD4_gi757864285:0.00000039,(Kosakonia_oryzae_gi1035730162:0.007
66322,Kosakonia_radicincitans_gi763072455:0.00000001)0.000000:0.00000085)0.00000
0:0.00000089)0.918000:0.01605736)0.000000:0.00000092,(Enterobacter_sp._Bisph1_gi
745756995:0.02366501,(Enterobacter_sp._FY-
07_gi1003370161:0.00000001,Enterobacter_sp._FY-
07_gi1004039482:0.00000001)0.772000:0.00815158)0.759000:0.00733108)0.393000:0.00
767508)0.927000:0.03160839,((Trabulsiella_guamensis_gi740317338:0.00758144,(Trab
ulsiella_odontotermitis_gi930177983:0.01545281,Trabulsiella_odontotermitis_gi908
718699:0.00000021)0.000000:0.00000021)0.994000:0.05788990,((Enterobacter_sp._MT2
0_gi1005724462:0.03199342,(Leclercia_adecarboxylata_gi743514789:0.02103502,(Lecl
ercia_adecarboxylata_gi695645306:0.02563537,(Enterobacter_cloacae_gi822027032:0.
00000001,Enterobacteriaceae_bacterium_ATCC_29904_gi685438728:0.00000001)0.882000

:0.02261769)0.911000:0.03066667)0.958000:0.04762322)0.794000:0.02016643,(((Kluyv
era_gi1035707571:0.02697331,((Kluyvera_cryocrescens_gi1001728049:0.01558827,Kluy
vera_cryocrescens_gi917771597:0.00000055)0.806000:0.03217225,(Kluyvera_ascorbata
_gi737931350:0.03067220,Kluyvera_intermedia_gi1011973833:0.05781148)0.039000:0.0
1787019)0.803000:0.02018515)0.421000:0.00823739,((Enterobacter_lignolyticus_gi50
3132076:0.00000076,Klebsiella_sp._G5_gi1011931743:0.00770331)0.956000:0.03781596
,(Enterobacteriaceae_bacterium_strain_FGI_57_gi440047613:0.00000001,Enterobacter
iaceae_bacterium_strain_FGI_57_gi754029843:0.00000001)0.977000:0.06429818)0.5880
00:0.00606466)0.891000:0.02441385,((Escherichia_vulneris_gi755040092:0.00754663,
((Enterobacter_cancerogenus_gi493172396:0.01372271,(((Mangrovibacter_sp._MFB070_
gi738150620:0.00000102,Mangrovibacter_sp._MP23_gi1035796725:0.00779450)0.999000:
0.15202596,(Enterobacteriaceae_gi488393597:0.12462558,(Siccibacter_colletis_gi73
7925726:0.01028792,Enterobacteriaceae_gi639212997:0.03789486)0.967000:0.06666288
)0.829000:0.02738293)0.000000:0.00348310,(Yokenella_regensburgei_gi740425781:0.0
0000053,Yokenella_regensburgei_gi750621595:0.00794382)0.952000:0.03319217)0.8970
00:0.03414769)0.000000:0.00305268,((Lelliottia_amnigena_gi1032819500:0.01642721,
(Enterobacter_sp._638_gi145318080:0.00000001,(Enterobacter_sp._638_gi754036586:0
.00000001,Lelliottia_amnigena_gi974588064:0.00000002)0.000000:0.00000029)0.89800
0:0.02273531)0.946000:0.03886117,((Enterobacter_asburiae_gi752454217:0.00000001,
Enterobacter_soli_gi503835733:0.00000029)0.000000:0.00000001,((Enterobacter_asbu
riae_gi765031015:0.00000001,Enterobacter_cloacae_complex_sp._35618_gi1043348544:
0.00000001)0.855000:0.00748136,((Enterobacter_cloacae_complex_gi495778930:0.0000
0001,(Enterobacter_cloacae_complex_sp._GN05526_gi1022674080:0.00000001,(Enteroba
cter_cloacae_gi1016942390:0.00000001,(Enterobacter_cloacae_complex_gi976152867:0
.00000001,(Enterobacter_cloacae_complex_gi556301345:0.00000001,(Enterobacter_clo
acae_complex_'Hoffmann_cluster_IV'_gi1022697867:0.00000001,(Enterobacter_cloacae
_complex_sp._GN03164_gi1043322400:0.00000001,Enterobacter_cloacae_complex_sp._CI
DEIMsCOL1_gi1043336184:0.00000001)0.000000:0.00000001)0.000000:0.00000001)0.0000
00:0.00000001)0.000000:0.00000001)0.000000:0.00000001)0.000000:0.00000001)0.0000
00:0.00000052,((Enterobacter_asburiae_gi1040476462:0.00769775,Enterobacter_cloac
ae_complex_gi1043336112:0.00747113)0.000000:0.00000085,((Enterobacter_cloacae_co
mplex_'Hoffmann_cluster_IV'_gi695748250:0.00749721,Enterobacter_mori_gi498115394
:0.00747902)0.000000:0.00000036,(((Enterobacter_cloacae_gi1027682480:0.00767408,
Enterobacter_cloacae_complex_sp._SMART_763_gi1043336248:0.00751803)0.000000:0.00
000086,(Enterobacter_ludwigii_gi748764957:0.00749008,(((Enterobacter_cloacae_gi1
028108832:0.00000002,(Enterobacter_cloacae_complex_gi823297144:0.00000033,Entero
bacteriaceae_gi979836662:0.00000001)0.000000:0.00000042)0.829000:0.00744197,((En
terobacter_sp._MGH119_gi851892708:0.00743799,(Enterobacter_hormaechei_subsp._oha
rae_gi770979665:0.00000001,Enterobacter_hormaechei_gi1040990523:0.00000001)0.854
000:0.00743932)0.000000:0.00000041,(Enterobacteriaceae_gi975891345:0.00000001,(E
nterobacter_cloacae_complex_gi550795278:0.00000001,(((Enterobacter_hormaechei_gi
975887058:0.00743704,(Enterobacter_hormaechei_subsp._oharae_gi771370542:0.000000
01,Enterobacter_hormaechei_gi1040990554:0.00000001)0.841000:0.00752222)0.000000:
0.00000041,(Enterobacter_hormaechei_subsp._oharae_gi728965830:0.00750244,(Entero
bacter_hormaechei_gi779819138:0.00000001,Enterobacter_hormaechei_gi1040988915:0.
00000001)0.843000:0.00774481)0.000000:0.00000086)0.000000:0.00000082,(Enterobact
er_cloacae_gi1016955547:0.00000001,(Enterobacter_cloacae_gi838623390:0.00000001,
(Enterobacter_cloacae_gi1007441952:0.00000001,Enterobacter_cloacae_complex_gi489
954763:0.00000001)0.000000:0.00000030)0.000000:0.00000055)0.000000:0.00000074)0.
000000:0.00000065)0.000000:0.00000071)0.000000:0.00000035)0.000000:0.00000087)0.
917000:0.01622824,(Enterobacter_cloacae_gi1014934348:0.00000001,Enterobacter_clo
acae_gi1016954155:0.00000001)0.816000:0.00747984)0.000000:0.00000033)0.000000:0.
00000032)0.000000:0.00000061,(Enterobacter_cloacae_complex_gi1028088342:0.000000
01,(Enterobacter_cloacae_complex_gi980965227:0.00000001,(Enterobacter_cloacae_co
mplex_gi1005936231:0.00000001,(Enterobacter_gi556426169:0.00000001,(Enterobacter
iaceae_gi640463989:0.00000001,(Enterobacter_cloacae_gi1028176220:0.00000001,(Ent
erobacter_cloacae_complex_gi981300785:0.00000001,(Enterobacter_cloacae_complex_s
p._CIDEIMsCOL9_gi1043336125:0.00000001,(Enterobacter_cloacae_complex_gi980271814
:0.00000001,(Enterobacter_cloacae_gi1044864171:0.00000001,(Enterobacter_cloacae_
complex_gi503935517:0.00000001,(Enterobacter_cloacae_gi1028102863:0.00000001,(En

terobacter_gi979845903:0.00000001,(Enterobacter_cloacae_gi1044777006:0.00000001,
(Enterobacter_cloacae_P101_gi570288821:0.00754570,((Enterobacter_asburiae_gi1039
425996:0.00000001,((Enterobacter_sp._MGH_24_gi555170073:0.00000001,(Enterobacter
_gi658547695:0.00000001,(Enterobacter_cloacae_complex_gi980945276:0.00000001,(En
terobacter_asburiae_gi979835436:0.00000001,(Enterobacter_cloacae_complex_gi97589
0959:0.00000001,(Enterobacter_cloacae_gi1028181223:0.00000001,((Enterobacter_clo
acae_gi1044710616:0.00736950,(Enterobacter_gi556481084:0.00000001,Enterobacter_g
i504696220:0.00000035)0.000000:0.00000001)0.000000:0.00000033,(Enterobacter_asbu
riae_gi949704834:0.00737162,Enterobacter_cloacae_complex_gi980951070:0.00000002)
0.000000:0.00000033)0.936000:0.00738535)0.000000:0.00000001)0.000000:0.00000001)
0.000000:0.00000001)0.000000:0.00000001)0.000000:0.00000039)0.000000:0.00000039,
((Enterobacter_cloacae_gi998245016:0.00000001,(((Enterobacter_cloacae_gi10160882
23:0.01520365,((Enterobacter_hormaechei_gi493862551:0.00000001,Enterobacter_cloa
cae_complex_gi695747282:0.00000001)0.922000:0.00759919,((Enterobacter_cloacae_co
mplex_sp._GN06232_gi1022698409:0.00000001,Enterobacter_cloacae_gi1028112161:0.00
000001)0.926000:0.00742159,((Enterobacter_hormaechei_subsp._steigerwaltii_gi7714
34629:0.00000001,Enterobacter_hormaechei_gi1040791966:0.00000001)0.854000:0.0073
8176,(Enterobacter_hormaechei_gi1040987627:0.00000034,(Enterobacteriaceae_gi1016
090293:0.00000032,(Enterobacter_cloacae_complex_gi695743850:0.00000040,(Enteroba
cter_cloacae_gi1016936594:0.00000001,(Enterobacter_cloacae_gi1028201692:0.000000
01,((Enterobacter_hormaechei_subsp._steigerwaltii_gi829410088:0.00000001,(Entero
bacter_cloacae_gi1028202160:0.00000001,Enterobacter_hormaechei_gi1040991629:0.00
000002)0.000000:0.00000034)0.840000:0.00739247,(Enterobacter_cloacae_complex_gi6
92191118:0.00000001,Enterobacter_cloacae_complex_gi1016933117:0.00000001)0.85800
0:0.00741545)0.000000:0.00000042)0.000000:0.00000089)0.000000:0.00000034)0.00000
0:0.00000027)0.000000:0.00000041)0.839000:0.00738654)0.000000:0.00000059)0.00000
0:0.00000034)0.000000:0.00000034)0.000000:0.00000036,((Enterobacter_cloacae_gi10
34517612:0.00000001,((Enterobacter_hormaechei_gi1041907717:0.00000001,Enterobact
er_hormaechei_subsp._hormaechei_gi829428636:0.00000001)0.864000:0.00738362,(Ente
robacter_cloacae_complex_gi704510475:0.00000001,Enterobacter_cloacae_complex_gi9
81222544:0.00000001)0.829000:0.00738757)0.000000:0.00000050)0.000000:0.00000001,
(Enterobacter_hormaechei_gi1044819376:0.00000001,(Enterobacteriaceae_gi981232855
:0.00000001,(Enterobacter_cloacae_complex_gi556390431:0.00000001,(Enterobacter_c
loacae_gi1016932813:0.00000001,(Enterobacter_hormaechei_gi981222056:0.00000001,(
Enterobacteriaceae_gi550714803:0.00000001,(Enterobacter_cloacae_gi1016937651:0.0
0000001,(Enterobacter_cloacae_gi1016951091:0.00000001,(Enterobacteriaceae_gi5159
54179:0.00000035,(Enterobacter_cloacae_gi1028092123:0.00000001,(Enterobacter_clo
acae_gi1016947744:0.00738583,(Enterobacter_sp._BIDMC93_gi851926337:0.00739197,(E
nterobacter_hormaechei_gi771114657:0.00000001,Enterobacter_hormaechei_gi10433145
49:0.00000001)0.844000:0.00742090)0.000000:0.00000054)0.000000:0.00000029)0.0000
00:0.00000001)0.000000:0.00000074)0.000000:0.00000053)0.000000:0.00000080)0.0000
00:0.00000041)0.000000:0.00000033)0.000000:0.00000027)0.000000:0.00000014)0.0000
00:0.00000007)0.000000:0.00000004)0.000000:0.00000037)0.921000:0.01610019,(Enter
obacter_cloacae_gi1014983497:0.00000001,(Enterobacter_cloacae_gi1016090484:0.000
00001,((Proteobacteria_gi502862207:0.00000001,(Enterobacter_cloacae_gi1006478746
:0.00000001,(Enterobacter_cloacae_gi1011917781:0.00000026,Enterobacter_gi9834125
10:0.00000001)0.000000:0.00000087)0.000000:0.00000071)0.000000:0.00000033,(Enter
obacter_cloacae_gi1040987830:0.00000040,(Enterobacter_cloacae_gi949730040:0.0150
5205,(Enterobacter_cloacae_subsp._cloacae_gi967183324:0.00000001,Enterobacter_cl
oacae_gi1011858439:0.00000001)0.824000:0.00760632)0.000000:0.00000057)0.000000:0
.00000033)0.913000:0.00742847)0.000000:0.00000002)0.000000:0.00000050)0.000000:0
.00000031)0.853000:0.00740667,(Enterobacter_cloacae_gi1022653826:0.00000001,Ente
robacter_cloacae_gi504644295:0.00000001)0.853000:0.00735665)0.000000:0.00000054)
0.000000:0.00000034)0.922000:0.01517529,(Enterobacter_cloacae_gi1020067039:0.007
48321,Enterobacter_cloacae_gi1028189566:0.00000087)0.843000:0.00748081)0.000000:
0.00000054)0.000000:0.00000089)0.000000:0.00000053)0.000000:0.00000029)0.000000:
0.00000040)0.000000:0.00000022)0.000000:0.00000012)0.000000:0.00000007)0.000000:
0.00000004)0.000000:0.00000002)0.000000:0.00000001)0.000000:0.00000001)0.000000:
0.00000001)0.000000:0.00000001)0.000000:0.00000001)0.000000:0.00000050)0.926000:
0.00748454)0.000000:0.00000031)0.000000:0.00000041)0.000000:0.00000036)0.858000:

0.00748075)0.000000:0.00000081)0.803000:0.00752902)0.932000:0.02316846)0.740000:
0.00780622,((Enterobacter_massiliensis_gi763319299:0.02354989,(Enterobacter_sp._
Bisph2_gi917734968:0.00000001,(Kluyvera_intermedia_gi920153392:0.00000002,Kluyve
ra_intermedia_gi828939082:0.00000050)0.000000:0.00000046)0.965000:0.03988980)0.6
61000:0.00843553,(Klebsiella_sp._RIT-PI-
d_gi908708632:0.07391838,(Klebsiella_pneumoniae_gi641639603:0.00000042,((Klebsie
lla_quasipneumoniae_gi896036885:0.00000001,(Enterobacteriaceae_gi488988147:0.000
00001,(Klebsiella_variicola_gi918630814:0.00000055,((Klebsiella_oxytoca_gi103270
2282:0.00774578,Klebsiella_pneumoniae_gi983450617:0.00773908)0.000000:0.00000065
,(Klebsiella_quasipneumoniae_subsp._quasipneumoniae_gi591288023:0.00000001,(Kleb
siella_pneumoniae_gi759827373:0.00784840,Klebsiella_pneumoniae_342_gi206564943:0
.00000001)0.000000:0.00000033)0.000000:0.00000001)0.000000:0.00000030)0.000000:0
.00000087)0.000000:0.00000087)0.000000:0.00000093,(Klebsiella_pneumoniae_gi89588
3282:0.00774080,((Klebsiella_pneumoniae_IS39_gi571164652:0.00795058,(Klebsiella_
pneumoniae_subsp._pneumoniae_NTUH-
K2044_gi238546403:0.00000001,Klebsiella_pneumoniae_gi1031831971:0.00774908)0.000
000:0.00000034)0.000000:0.00000051,((Klebsiella_michiganensis_gi695828560:0.0000
0078,(((Klebsiella_oxytoca_10-
5245_gi376387778:0.00000001,Klebsiella_oxytoca_gi515196144:0.00785942)0.000000:0
.00000054,(((Klebsiella_oxytoca_gi695790419:0.00000001,Klebsiella_oxytoca_10-
5243_gi376386493:0.00000001)0.914000:0.00799558,((Klebsiella_oxytoca_gi920858114
:0.00000001,Klebsiella_oxytoca_gi838846478:0.00000001)0.919000:0.00782181,((Kleb
siella_oxytoca_gi1033036175:0.00000001,Klebsiella_oxytoca_gi1031455839:0.0000000
1)0.913000:0.00784806,((Raoultella_ornithinolytica_gi751930221:0.00000086,Raoult
ella_terrigena_gi797193061:0.01573896)0.967000:0.03168314,(Klebsiella_oxytoca_gi
1033059706:0.00000001,Klebsiella_oxytoca_gi1031595090:0.00000001)0.806000:0.0078
3007)0.000000:0.00000053)0.000000:0.00000038)0.000000:0.00000086)0.000000:0.0000
0057,(Klebsiella_oxytoca_gi515713545:0.00000001,(Klebsiella_oxytoca_MGH_42_gi555
130314:0.00000001,(Klebsiella_oxytoca_gi1031353960:0.00000001,(Klebsiella_oxytoc
a_MGH_28_gi555157385:0.00000001,Klebsiella_oxytoca_gi1031405494:0.00000069)0.000
000:0.00000091)0.000000:0.00000074)0.000000:0.00000080)0.000000:0.00000080)0.000
000:0.00000032)0.970000:0.02437947,((Klebsiella_gi503994813:0.00000032,(Klebsiel
la_oxytoca_gi1031444607:0.00000001,(Klebsiella_oxytoca_gi1031257337:0.00000001,(
Klebsiella_oxytoca_gi1031320848:0.00000001,(Klebsiella_gi695769853:0.00000001,Kl
ebsiella_oxytoca_gi1031492692:0.00000001)0.934000:0.00779792)0.000000:0.00000067
)0.000000:0.00000088)0.000000:0.00000089)0.843000:0.00779611,(Klebsiella_gi49100
9554:0.00000001,(Klebsiella_oxytoca_gi1031429598:0.00000001,Klebsiella_oxytoca_g
i1031304503:0.00000039)0.000000:0.00000080)0.000000:0.00000059)0.828000:0.007766
38)0.000000:0.00000083)0.986000:0.05772687,((Enterobacteriaceae_gi695772851:0.00
000001,(Klebsiella_oxytoca_gi1031329456:0.00000050,(Raoultella_ornithinolytica_1
0-
5246_gi376398347:0.00000001,Enterobacteriaceae_gi505397562:0.00000001)0.989000:0
.04737599)0.000000:0.00000050)0.944000:0.03668164,(Enterobacter_gi779949135:0.00
000056,(Enterobacter_aerogenes_UCI_46_gi578194439:0.00000001,(Enterobacter_gi695
800081:0.00774916,(Enterobacteriaceae_gi505180129:0.00000016,(Enterobacter_gi779
966467:0.00778455,(Enterobacter_gi823298988:0.00772313,Enterobacter_gi823285607:
0.00773691)0.000000:0.00000092)0.000000:0.00000029)0.000000:0.00000016)0.000000:
0.00000092)0.827000:0.00772474)0.856000:0.01727649)0.711000:0.00687645)0.739000:
0.00800467)0.000000:0.00000085)0.000000:0.00000089)0.000000:0.00000094)0.959000:
0.04021406)0.741000:0.00944686)0.780000:0.00821770)0.746000:0.01196299)0.675000:
0.01602014)0.000000:0.00000152)0.834000:0.01797096)0.821000:0.01614872)0.813000:
0.01425417)0.789000:0.01639515)0.802000:0.01498486)0.963000:0.06327708)0.561000:
0.02375627)0.998000:0.14622266)0.000000:0.00226690)0.806000:0.00813442)0.876000:
0.01595525)0.000000:0.00000056)0.822000:0.02372952,((Serratia_rubidaea_gi1001786
148:0.00791892,Serratia_gi505487223:0.00000090)0.992000:0.07616713,(Serratia_odo
rifera_gi491103049:0.01246408,((Serratia_marcescens_gi983124078:0.00795124,((Ser
ratia_gi491073104:0.00776110,Serratia_marcescens_gi1028643611:0.00776846)0.00000
0:0.00000082,(Serratia_marcescens_gi896196120:0.00772945,(Serratia_marcescens_gi
727191886:0.00775319,Enterobacteriaceae_gi505191124:0.00000034)0.000000:0.000000
52)0.000000:0.00000060)0.000000:0.00000060)0.772000:0.00772459,(Serratia_ficaria

```
_gi1006545375:0.00000051,(Serratia_gi525771777:0.01623724,Serratia_gi491084289:0
.00000090)0.973000:0.04396605)0.759000:0.00790778)0.846000:0.01954145)0.131000:0
.01331811)0.770000:0.01542289)1.000000:0.39812371)0.000000:0.00000057)0.000000:0
.00000054);
```
